# VDJ-REMIX: REpertoire Module Identification and eXploration

**DOI:** 10.1093/bioinformatics/btag326

**Published:** 2026-07-07

**Authors:** Sakina Amin, Lauren Overend, Felicia Tucci, Bo Sun, Justin Whalley, Michael L Dustin, Julian C Knight, Rachael Bashford-Rogers

**Affiliations:** Department of Biochemistry, University of Oxford, Oxford OX1 3QU, United Kingdom; Centre for Human Genetics, Nuffield Department of Medicine, University of Oxford, Oxford OX3 7BN, United Kingdom; Centre for Human Genetics, Nuffield Department of Medicine, University of Oxford, Oxford OX3 7BN, United Kingdom; Nuffield Department of Clinical Neuroscience, University of Oxford, Oxford OX3 9DU, United Kingdom; Centre for Human Genetics, Nuffield Department of Medicine, University of Oxford, Oxford OX3 7BN, United Kingdom; Chicago Medical School, Rosalind Franklin University of Medicine and Science, North Chicago, IL 60064, United States; Kennedy Institute of Rheumatology, University of Oxford, Oxford OX3 7FY, United Kingdom; Centre for Human Genetics, Nuffield Department of Medicine, University of Oxford, Oxford OX3 7BN, United Kingdom; Department of Biochemistry, University of Oxford, Oxford OX1 3QU, United Kingdom; Centre for Human Genetics, Nuffield Department of Medicine, University of Oxford, Oxford OX3 7BN, United Kingdom

## Abstract

**Motivation:**

High-throughput sequencing of B and T cell repertoires provides unprecedented insights into adaptive immunity but generates high-dimensional feature sets that are challenging to interpret. Standard dimensionality reduction techniques are often suboptimal for adaptive immune receptor repertoire (AIRR) data, which exhibits multi-collinearity, heterogeneous data types, and missingness.

**Results:**

Here, we present VDJ-REMIX, an R package implementing a robust, network-based framework to deconstruct complex repertoire feature matrices into biologically interpretable modules. By refactoring weighted correlation network analysis (WGCNA), VDJ-REMIX provides a tailored workflow for preprocessing, imputation, and modularization of immune repertoire data. We demonstrate its utility across diverse contexts, including autoimmunity, inflammation, and acute infection. In autoimmune patients, VDJ-REMIX identified distinct B cell signatures that stratified diseases and revealed opposing dynamic responses to B cell-depleting versus anti-proliferative therapies. In COVID-19 and non-COVID-19 sepsis patients, it distinguished disease-specific signatures from shared severe infection responses and identified modules correlated with severity. Analysis of flow-sorted B cell populations stratified by FCGR2B genotype recapitulated known tolerance defects and uncovered population-specific repertoire signatures linked to inhibitory receptor dysfunction, providing orthogonal validation of module biological coherence. Finally, applied to a single-cell multi-omics dataset of immune cells in pancreatic ductal adenocarcinoma (PDAC) combining gene expression with AIRR-seq, VDJ-REMIX recovered modules linking BCR isotype usage and clonality to cytotoxic, interferon-responsive, and regulatory immune programmes. VDJ-REMIX is a versatile tool enabling systematic exploration of immunological variation and biomarker discovery from complex immune repertoire data.

**Availability and implementation:**

VDJ-REMIX is freely available at https://github.com/Bashford-Rogers-lab/vdjremix.

## 1 Introduction

Adaptive immunity arises from diverse cell populations such as B, T and natural killer cells whose antigen receptors collectively shape host defence. B and T cell clones selectively expand following antigen recognition by B cell receptors (BCRs) and T cell receptors (TCRs). Somatic VDJ recombination processes generate extensive receptor diversity, enabling lymphocyte populations to sample a vast antigenic space. This process creates a unique receptor (BCR or TCR) for each cell. Further diversity in B cells is achieved through somatic hypermutation (SHM) and class switching (CSR). B cells and T cells have the potential to recognize a vast array of pathogens (Chi *et al.* 2020). Sequencing of the B cell receptor (BCR) and T cell receptor (TCR) repertoire is performed to investigate the dynamics of adaptive immune responses in various contexts, such as immune-mediated diseases (Bashford-Rogers *et al.* 2019), ageing (Wang *et al.* 2025), and cancer (Sivakumar *et al.* 2025). BCR and TCR repertoire features represent biomarkers of predictive and prognostic significance (Mayer-Blackwell *et al.* 2021) that can be implemented in patient risk stratification models, understanding of disease mechanisms and for the development of therapeutics (Leem *et al.* 2022). High-throughput adaptive immune repertoire sequencing generates large, complex datasets, which require specialist analysis pipelines, distinct from those used for conventional RNA-seq analysis. This is because of the multi-collinearity of features, heterogeneous data types, and feature missingness across samples.

In recent years, AIRR-seq methods span sequence-level processing (Lefranc *et al.* 2009, Christley *et al.* 2017, Marcou *et al.* 2018), summary feature extraction (Bashford-Rogers *et al.* 2013, Wang *et al.* 2017, Bashford-Rogers *et al.* 2019, Xu-Monette *et al.* 2019, Sivakumar *et al.* 2025), and higher-order modelling approaches (Wu *et al.* 2021, Leem *et al.* 2022, Kenlay *et al.* 2024). While powerful, this multi-stage process often yields hundreds of metrics per sample each describing different aspects of the repertoire. Subsequent data mining to identify disease-specific pathophysiology from this high-dimensional feature space can be complex, difficult to interpret, and incurs a significant multiple-testing burden.

Therefore, there is a clear need for computational methods optimized for the dimensional reduction of immune repertoire feature matrices. An effective approach would distill numerous features into a smaller set of informative components that capture the main axes of biological variation. Dimensional reduction techniques, such as Principal Component Analysis (PCA), have been widely used across multi-omics data types to achieve this (Cantini *et al.* 2021), enabling the identification of features that distinguish sample groups and reveal novel patient subgroups in an unsupervised manner (Meng *et al.* 2016). Weighted Gene Correlation Network Analysis (WGCNA) is a particularly powerful dimensional reduction approach developed to resolve large gene expression matrices into a smaller number of ‘modules’ representing correlated gene networks (Langfelder and Horvath 2008). These modules are often interrogated using gene ontology and pathway enrichment analyses to link co-expression networks to biological processes implicated in a trait of interest (Liang *et al.* 2018, Yao *et al.* 2019, Farhadian *et al.* 2021). However, a typical BCR repertoire feature matrix, comprised of over 500 summary metrics across isotypes, differs substantially from a gene expression matrix in ways that can impact the performance of standard WGCNA. A repertoire matrix is orders of magnitude smaller than a typical transcriptome, is composed of heterogeneous data types (e.g. proportions, ratios, diversity indices), and often contains missing data due to variations in isotype capture or sub-sampling strategies.

Considering these fundamental differences, we developed VDJ-REMIX, a computational framework that refactors and adapts the principles of WGCNA for robust analysis of AIRR-seq data. This work details the development of our custom pipeline, evaluates its performance, and demonstrates its utility in resolving complex immunological signatures from high-dimensional repertoire feature matrices.

## 2 Materials and methods

### 2.1 Dataset acquisition and pre-processing

BCR repertoire sequencing datasets were analysed across multiple clinical contexts. The COvid-19 Multi-omics Blood ATlas (COMBAT) cohort (*n* = 78) was used to assess COVID-19 versus healthy and sepsis patients (COMBAT Consortium 2022). Flow-sorted B cell data (*n* = 29) generated by Espeli *et al.* was used to validate repertoire module structure across defined B cell compartments (Espéli *et al.* 2019). Immune-mediated disease repertoires (*n* = 237) were analysed using the cohort reported by Bashford-Rogers *et al.* (Bashford-Rogers *et al.* 2019). Single cell PDAC gene expression and BCR/TCR sequencing data (*n* = 12) was used to apply VDJ-REMIX on single cell multi-omics data from Sivakumar *et al.* (Sivakumar *et al.* 2025). Raw sequencing reads were processed to generate a high-dimensional repertoire feature matrix (typically >400 metrics per sample), capturing isotype usage, V/J gene segment usage, CDR3 physicochemical properties, clonality/expansion statistics, and somatic hypermutation (SHM) features using https://github.com/rbr1/Immune_receptor_NETWORK-GENERATION (Bashford-Rogers *et al.* 2013). Details on feature calculations are provided in the Supplementary Methods, available as supplementary data at *Bioinformatics* online.

### 2.2 Feature filtering

To improve statistical stability and reduce artefactual correlation structure driven by sparse or low-information metrics, we filtered the repertoire feature matrix prior to network construction. Features were excluded if they had (i) high missingness (>40% NA values across samples) or (ii) low information content (<8 unique observed values across the cohort). Per-sample missingness was summarized and used to guide downstream imputation robustness checks (details in Supplementary Methods, available as supplementary data at *Bioinformatics* online).

### 2.3 VDJ-REMIX network construction

VDJ-REMIX is a repertoire module reduction framework inspired by WGCNA principles, designed to summarize high-dimensional repertoire features into interpretable modules.

### 2.4 Missing-data handling and imputation

Missing repertoire values can reflect both biological absence (e.g. missing isotypes or undetected gene usage) and sampling depth limitations. Because eigengene construction requires a complete matrix, we used a two-stage imputation strategy: (i) method selection and robustness filtering, followed by (ii) final imputation for downstream module eigengene computation. To select an imputation approach, we performed controlled missingness seeding on a complete-case subset and compared multiple strategies using reconstruction accuracy on masked entries (Supplementary Methods, available as supplementary data at *Bioinformatics* online). Random Forest imputation (missForest; maxiter =20) performed best across missingness levels and was used for final imputation. After robustness filtering, the full dataset was imputed with missForest and then z-scored feature-wise prior to PCA-based eigengene calculation. Full benchmarking, error metrics, diagnostic checks, number of features retained after filtering are provided in the Supplementary Materials, available as supplementary data at *Bioinformatics* online.

### 2.5 Robust correlation estimation by subsampling

To minimize dependence of network topology on imputed values, correlations were estimated on the filtered *non-imputed* matrix using repeated subsampling over pairwise complete overlaps. For each feature pair (i,j), let $S_{ij}$ denote samples where both features are observed. A global subsampling depth was set as


$$k=0.8\times \underset{i<j}{\min}|S_{ij}|,$$

and the pairwise correlation was estimated as the mean Pearson correlation over *B* repeated subsamples (sampling without replacement within $S_{ij}$):


$${\hat{r}}_{ij}=\frac{1}{B}\sum_{b=1}^{B}\text{cor}\left(X_{i}[T_{ij}^{\left(b\right)}],X_{j}[T_{ij}^{\left(b\right)}]\right).$$

Unless otherwise stated, we used B=10,000 repeats. This produces a stable correlation matrix under heterogeneous missingness (implementation details in Supplementary Methods, available as supplementary data at *Bioinformatics* online).

### 2.6 Distance definition and hierarchical clustering

To preserve correlation sign, we defined a signed distance


$$D_{ij}=\frac{1-{\hat{r}}_{ij}}{2}.$$

Feature modules were identified via hierarchical clustering on *D* and dendrogram cutting as described below (full parameterization in Supplementary Methods, available as supplementary data at *Bioinformatics* online).

### 2.7 Module detection and eigengene computation

We obtained modules using complementary dendrogram partitioning approaches (fixed-height cutting to yield *K* clusters, and dynamic tree cutting with a minimum module size of 10). If dynamic cutting produced a more stable and parsimonious partition, it was used for downstream analyses; features unassigned by dynamic cutting were removed from eigengene computation and retained for diagnostics.

Modules were summarized by eigengenes computed on the *scaled, missForest-imputed* matrix. For each module *k* with $p_{k}\geq 2$ features, we performed PCA on the module submatrix and defined the eigengene as the first principal component score vector, $ME_{k}=\text{PC}1(X_{k})$. Eigengene signs were oriented to align with the module-wise mean feature profile. Independent uncorrelated features were used as independent modules with the scaled feature value.

### 2.8 Driver features and module membership

For multi-feature modules, we retained PC1 loadings for interpretation and defined driver features as those exceeding a uniform-chance loading threshold,$|l_{jk}|\geq 1/\sqrt{p_{k}}$. We also computed module membership (kME) as the correlation between each feature and its module eigengene, $kME_{jk}=\text{cor}(X_{j},ME_{k})$. Detailed reporting is provided in the Supplementary Methods, available as supplementary data at *Bioinformatics* online.

### 2.9 Therapy dynamics analysis

To assess the impact of immunosuppressive therapies on repertoire modules, we integrated clinical metadata with Module Eigengene scores. We analysed two specific cohorts: AAV and SLE.

- **Longitudinal trajectories:** Scatter plots were generated to visualize module dynamics over time (0–500 days) following RTX or MMF therapy for AAV only. Linear regression lines and Pearson correlation coefficients were calculated to assess temporal trends (ggpubr::ggscatter).
- **Treatment effect:** We compared module scores at baseline (‘At diagnosis’ or ‘At flare’) against early post-treatment timepoints (1–90 days post-therapy). Significant differences were determined using two-sided Wilcoxon rank-sum tests.

### 2.10 Differential module abundance across diseases

To compare repertoire features across multiple autoimmune conditions at diagnosis (AAV, SLE, Behçet’s, Crohn’s Disease, EGPA, IgAV) versus Healthy Controls (HC):

- **Global comparisons:** Differences in module distributions across all disease groups were assessed using one-way Analysis of Variance (ANOVA) or Kruskal-Wallis tests, followed by pairwise comparisons where appropriate.
- **Heatmap generation:** A summary heatmap was constructed to visualize disease-specific module signatures.
  - **Tile color (disease versus health):** For each module, we compared scores of each disease group against Healthy Controls using Wilcoxon rank-sum tests. *P*-values were adjusted for multiple testing using the Benjamini-Hochberg (BH) False Discovery Rate (FDR) method. Tiles were colored based on the direction of change (Higher/Lower) and significance level (FDR < 0.05 or FDR < 0.005).
  - **Significance markers (inter-disease heterogeneity):** To determine if a module varied significantly *across* the different disease states (excluding HCs), we performed a Kruskal-Wallis test. Modules exhibiting significant heterogeneity across diseases (FDR < 0.05) were marked with an asterisk (*).

### 2.11 Genotype-phenotype associations

To investigate the genetic basis of repertoire alterations, we tested for associations between specific host genotypes (Major versus Minor Homozygous) and module scores within defined populations. We used one-way ANOVA for each module-genotype pair. *P*-values were adjusted for multiple testing using the BH method. Significant associations (FDR < 0.05) were visualized in a heatmap indicating whether the Minor (TT) or Major (II) homozygous genotype was associated with higher module scores.

### 2.12 Trend analysis (disease severity)

To test for ordered progression of module scores across ordinal groups (e.g. Healthy < Mild COVID < Severe COVID), we utilized the Jonckheere-Terpstra test (clinfun::jonckheere.test) (Seshan and Whiting 2023). This non-parametric test evaluates the null hypothesis that the distribution of the response variable does not change across ordered groups against the alternative of a monotonic trend (increasing or decreasing). Results were FDR-corrected, and significant trends were visualized using boxplots with linear trend lines.

### 2.13 Supervised classification (sPLS-DA)

To discriminate disease states using repertoire modules, we applied sparse Partial Least Squares Discriminant Analysis (sPLS-DA) from mixOmics (Rohart *et al.* 2017). The input matrix *X* comprised module eigengenes (and where specified, a small set of engineered summary features). Model tuning selected the number of components (ncomp) and sparsity level via repeated M-fold cross-validation (5-fold CV, 100 repeats). Model performance was summarized using classification error and balanced error rate. Samples were visualized in the latent component space (PLS1 versus PLS2) with 95% confidence ellipses, and feature weights were used to identify discriminatory modules.

### 2.14 Dimensionality reduction of flow-sorted populations

To relate repertoire module structure to flow-sorted B cell subsets in the validation cohort, we performed PCA on the scaled eigengene matrix and visualized samples coloured by cell subset identity. This enabled assessment of whether module scores recapitulated expected biological differences between sorted populations.

### 2.15 Benchmarking against alternative dimensionality reduction methods

To contextualize the performance of VDJ-REMIX, we benchmarked it against two widely used approaches for dimensionality reduction and latent factor discovery: WGCNA and MOFA2 (Argelaguet *et al.* 2020). All methods were applied to the same imputed repertoire feature matrix using identical preprocessing, and the number of latent dimensions was matched across methods for comparability. We assessed three complementary criteria: (i) interpretability, quantified by the entropy of absolute feature loadings per latent dimension; (ii) representation efficiency, measured as the proportion of variance explained within each dimension; and (iii) predictive utility, evaluated via cross-validated classification performance on the autoimmune cohort.

### 2.16 Statistical analysis and software

All analyses were performed in R (v4.0.0) using tidyverse (Wickham *et al.* 2019) packages for data manipulation and plotting. Where appropriate, *P*-values were adjusted for multiple testing using the Benjamini–Hochberg false discovery rate (FDR).

## 3 Results

### 3.1 Overview of BCR repertoire matrix generation and VDJ-REMIX framework

**Figure 1:**
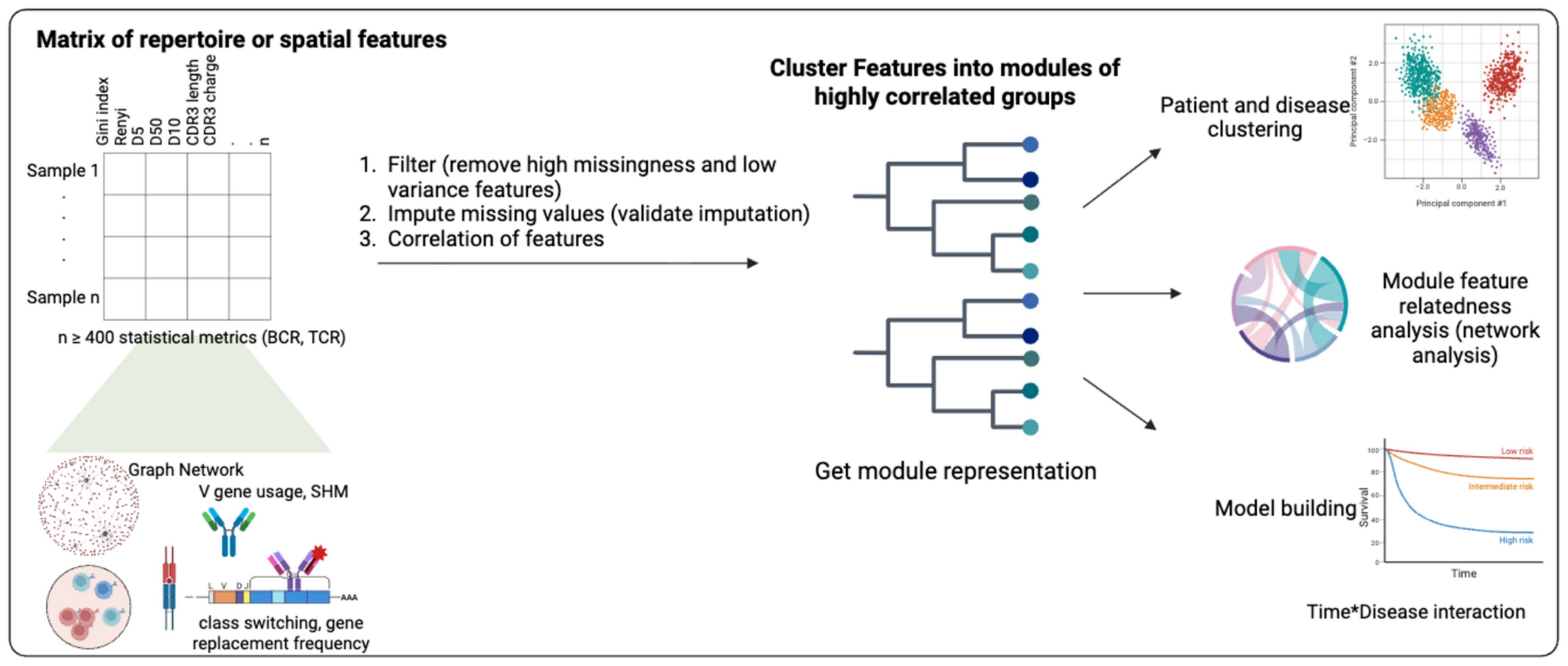
Schematic overview of the VDJ-REMIX framework. A matrix of quantitative repertoire features is processed through filtering, imputation, and weighted network clustering to identify modules of co-regulated features. Each module is summarized by its module eigengene. These enable downstream applications such as patient clustering, inter-module network analysis, and predictive model building.

High-throughput immune repertoire sequencing generates high-dimensional datasets that pose significant analytical challenges. To address this, we developed VDJ-REMIX, an R package that implements a comprehensive framework for the unsupervised dimensional reduction and modular analysis of repertoire features (Fig. 1, Fig. 1, available as supplementary data at *Bioinformatics* online). The central objective of VDJ-REMIX is to resolve the complexity of hundreds of correlated repertoire metrics into a small number of robust, biologically interpretable components, each representing a distinct axis of immunological variation and reducing the burden of multiple testing.

The analytical workflow begins with a matrix of quantitative repertoire features (rows = samples, columns = features), derived from metrics such as V-gene usage, somatic hypermutation (SHM) levels and ratios, clonal expansion indices (Gini, Renyi, D5), J gene usage, relative isotype switching and CDR3 length and physiochemical properties (Fig. 1A, available as supplementary data at *Bioinformatics* online). This matrix undergoes a rigorous preprocessing pipeline, including the filtering of features with high rates of missingness (>40%) and/or low variance (e.g. fewer than eight unique values) across a cohort to enhance the signal-to-noise ratio. Subsequently, any incomplete feature measurements must be addressed prior to correlation-based analysis (Fig. 1B, available as supplementary data at *Bioinformatics* online). The pipeline automatically evaluates eight different imputation strategies [e.g. k-nearest neighbours, missForest (Stekhoven and Bühlmann 2012), mean, median], enabling the user to select the optimal method for their dataset.

Following preprocessing, VDJ-REMIX identifies groups of repertoire features that change together across samples. We first estimated a robust pairwise Pearson correlation structure between features by repeatedly subsampling the overlapping non-missing samples for each feature pair and averaging correlations across many repeats. This produced a stable feature–feature correlation matrix despite missingness and heterogeneity in feature coverage. We then converted correlations into a signed dissimilarity $D_{ij}=(1-r_{ij})/2$ and performed hierarchical clustering to group strongly co-varying features into distinct modules (Fig. 1C, available as supplementary data at *Bioinformatics* online). Module boundaries were defined using DynamicTreeCut (Langfelder *et al.* 2008) and features not assigned to any stable cluster were retained as ‘unassigned’ rather than being forced into modules.

To summarize each module at the sample level, we calculated a module score (eigengene) as the first principal component (PC1) of the scaled features within the module. Eigengene direction was aligned so that higher scores correspond to higher average feature values within the module. Single-feature clusters were carried forward as scaled single-feature scores. This step reduced the repertoire feature space from hundreds of individual metrics to a concise set of module scores per sample, enabling downstream association testing and cross-cohort comparisons.

The resulting module-level data structure enables a suite of downstream quantitative analyses. These include, but are not limited to, clustering patients or disease states based on module scores, performing network analysis of inter-module correlations to understand the relationships between biological processes, and building predictive statistical models, such as survival or treatment-response models, using modules as predictors (Fig. 1, right). To demonstrate the versatility and power of the VDJ-REMIX framework, we applied it to analyse bulk B cell receptor sequencing data from three distinct biological contexts: autoimmunity, inflammatory diseases, and acute COVID-19 infection.

### 3.2 Stratification of autoimmune diseases and dynamics of differential treatment response

**Figure 2:**
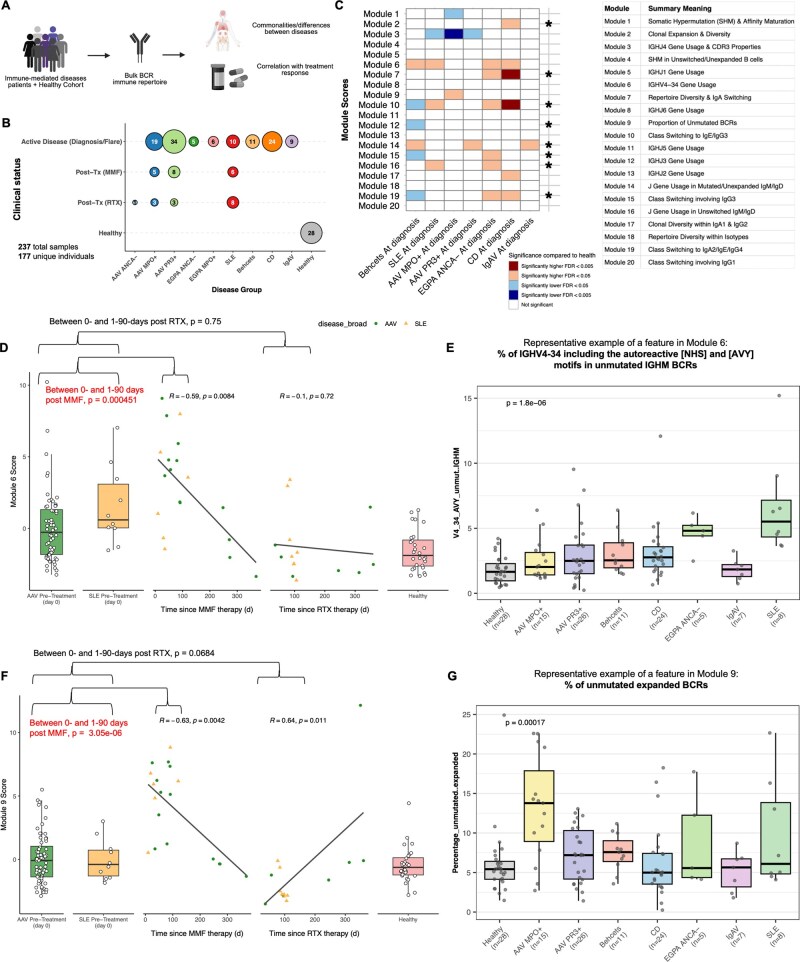
Stratification of autoimmune diseases and dynamics of differential treatment response. (A) Schematic of the study design. Bulk B cell receptor (BCR) immune repertoire data from a cohort of autoimmune patients and healthy donors was analysed to identify immunological signatures to compare them across diseases and correlate them with therapeutic responses. (B) Cohort composition showing the number of samples stratified by disease group and clinical status. (C) Heatmap showing the significance of module score differences between autoimmune diseases at diagnosis compared to healthy controls. Red and blue indicate significantly higher or lower module scores, respectively, after Benjamini–Hochberg (FDR) correction. (D) Left plots: Boxplots comparing module 6 scores between AAV and SLE patients at diagnosis. Centre: Scatter plots showing the correlation between module 6 scores and time (in days) since starting MMF or RTX therapy for AAV. Right: Module 6 scores in healthy controls for reference. *P*-value for at diagnosis versus early treatment were calculated using Wilcoxon test for AAV/SLE. (E) Boxplot of a representative module 6 feature, percentage of IGHV4-34 including the autoreactive [NHS] and [AVY] motifs within the unmutated IGHM BCRs. (F) Left plots: Boxplots comparing module 9 scores between AAV and SLE patients at diagnosis. Centre: Scatter plots showing the correlation between module 9 scores and time (in days) since starting MMF or RTX therapy for AAV. Right: Module 9 scores in healthy controls for reference. *P*-value for at diagnosis versus early treatment were calculated using Wilcoxon test for AAV/SLE. (G) Boxplot of a representative module 9 feature, percentage unmutated expanded BCRs at diagnosis across diseases.

To demonstrate the power of the method to resolve complex immunological heterogeneity, we applied it to a BCR repertoire from a cross-sectional cohort of patients with active disease, including ANCA-associated vasculitis (AAV), systemic lupus erythematosus (SLE), and Crohn’s disease, Behçet’s disease, eosinophilic granulomatosis with polyangiitis, and immunoglobulin A (IgA) vasculitis, compared to healthy controls (Bashford-Rogers *et al.* 2019) (*n* = 237 total samples; Fig. 2A and B). We used VDJ-REMIX to deconstruct the high-dimensional BCR feature space into 20 distinct modules of co-regulated features.

This modular analysis revealed disease-specific immunological fingerprints at diagnosis (Fig. 2C). The biological validity of these modules was evident, as they recapitulated known pathophysiology. For example, Module 7, encompassing features of CDR3 lengths is a critical determinant of antibody polyreactivity and autoimmunity (Bashford-Rogers *et al.* 2018, Lecerf *et al.* 2019, Boughter *et al.* 2020). Module 7 score was significantly elevated in EGPA ANCA- and Crohn’s disease, consistent with prior reports of altered CDR3 characteristics in these conditions further validating our method (Bashford-Rogers *et al.* 2019). Module 6 scores, which are driven by proportions of unmutated IGHV4-34 germline genes and implicated in autoreactivity (Chi *et al.* 2020), were significantly elevated across multiple autoimmune states, particularly SLE, Behçet’s, EGPA ANCA- and Crohn’s disease compared to healthy controls (FDR < 0.05). These initial findings validated our approach and highlighted modules representing distinct axes of B cell dysregulation observed in previous studies (Bashford-Rogers *et al.* 2019). Module 10 (comprising features of relative class switching between all isotypes to IGHE or IGHG3) was significantly lower (FDR < 0.05) compared to health in Behçet’s disease, although significantly higher in SLE (FDR < 0.05), EGPA ANCA- (FDR < 0.05) and Crohn’s disease (FDR < 0.005), consistent with previous research (Bashford-Rogers *et al.* 2019). Notably, the analysis also identified a previously unreported feature: IGHJ4 usage across isotypes (Module 3) was significantly lower in SLE and AAV MPO+/PR3+ than in healthy controls. This IGHJ-specific signal indicates that dysregulated B cell selection in these diseases is shaped not only by IGHV gene usage, but also by IGHJ composition. This aligns with structural studies showing that IGHJ4 and IGHJ6 contribute to antibody stability by shaping the CDR-H3 loop and partially influencing the paratope (Wang *et al.* 2021). Consistent with our findings, skewed IGHJ4 usage has been reported in multiple infectious and immunological conditions, including COVID-19 (Ma *et al.* 2022), Wiskott–Aldrich syndrome (O’Connell *et al.* 2014), and immune thrombocytopenia (Hirokawa *et al.* 2019).

Beyond cross-sectional stratification, VDJ-REMIX provides a quantitative framework for dissecting longitudinal responses to therapeutic intervention. We focused on the divergent clinical paradigms of AAV and SLE by assessing the impact of two immunomodulators with distinct mechanisms of action, namely following initiation of mycophenolate mofetil (MMF), which inhibits lymphocyte proliferation (Allison 2005), and after B cell depletion with rituximab (RTX) (Cerny *et al.* 2002) (Fig. 2D–G). Module 6 score at diagnosis was significantly elevated compared to health (Fig. 2D and E), which, upon early treatment with MMF (1–90 days), resulted in a significant increase in both SLE and AAV patients (*P* = .000451), then a significant linear reduction with time on MMF therapy in AAV patients (*R* = −0.87, *P* = 9.2e−05). Taken together, this trajectory supports complex B cell dynamics with MMF, consistent with MMF’s anti-proliferative effects effectively diminishing this potentially pathogenic, polyreactive B cell population after 1 year of treatment. In stark contrast, early treatment with RTX (1–90 days) resulted in no significant temporal correlation, indicating that this signature is not simply dependent on the overall CD20+ B cell count.

Module 9 also showed clear temporal associations with treatment, a signature defined by percentage of unmutated BCRs across all B cell isotypes. Comparing at diagnosis and early treatment with MMF (1–90 days), Module 9 substantially increased in both AAV and SLE patients (*P* = 1.05e−10, Fig. 2F). Over a year, Module 9 score linearly declined with time on MMF therapy (*R* = −0.74, *P* = .0036) in AAV patients. However, it displayed an opposing dynamic following RTX therapy, showing a strong positive correlation with time since therapy (*R* = 0.6, *P* = .15) in AAV patients. This observation is consistent with the known biology of B cell-depleting therapy, reflecting the homeostatic repopulation of the peripheral B cell pool primarily by new, unmutated naïve B cells emerging from the bone marrow (Cabatingan *et al.* 2002). Interestingly, the elevated percentage of unmutated BCRs in expanded clones was strongly associated at diagnosis in AAV MPO+ patients (*P* = .00017) and not AAV PR3+ patients, suggesting disease subtype-specific differences (Fig. 2G).

Thus, VDJ-REMIX not only uncovers static, disease-defining signatures but also provides a robust tool for longitudinally monitoring the mechanistic impact of therapies by capturing distinct B cell compartment dynamics, revealing how MMF broadly suppresses B cell activity while RTX initiates a selective reconstitution of the naive compartment.

### 3.3 VDJ-REMIX dissects shared and divergent BCR signatures between COVID-19 and non-COVID-19 sepsis

**Figure 3:**
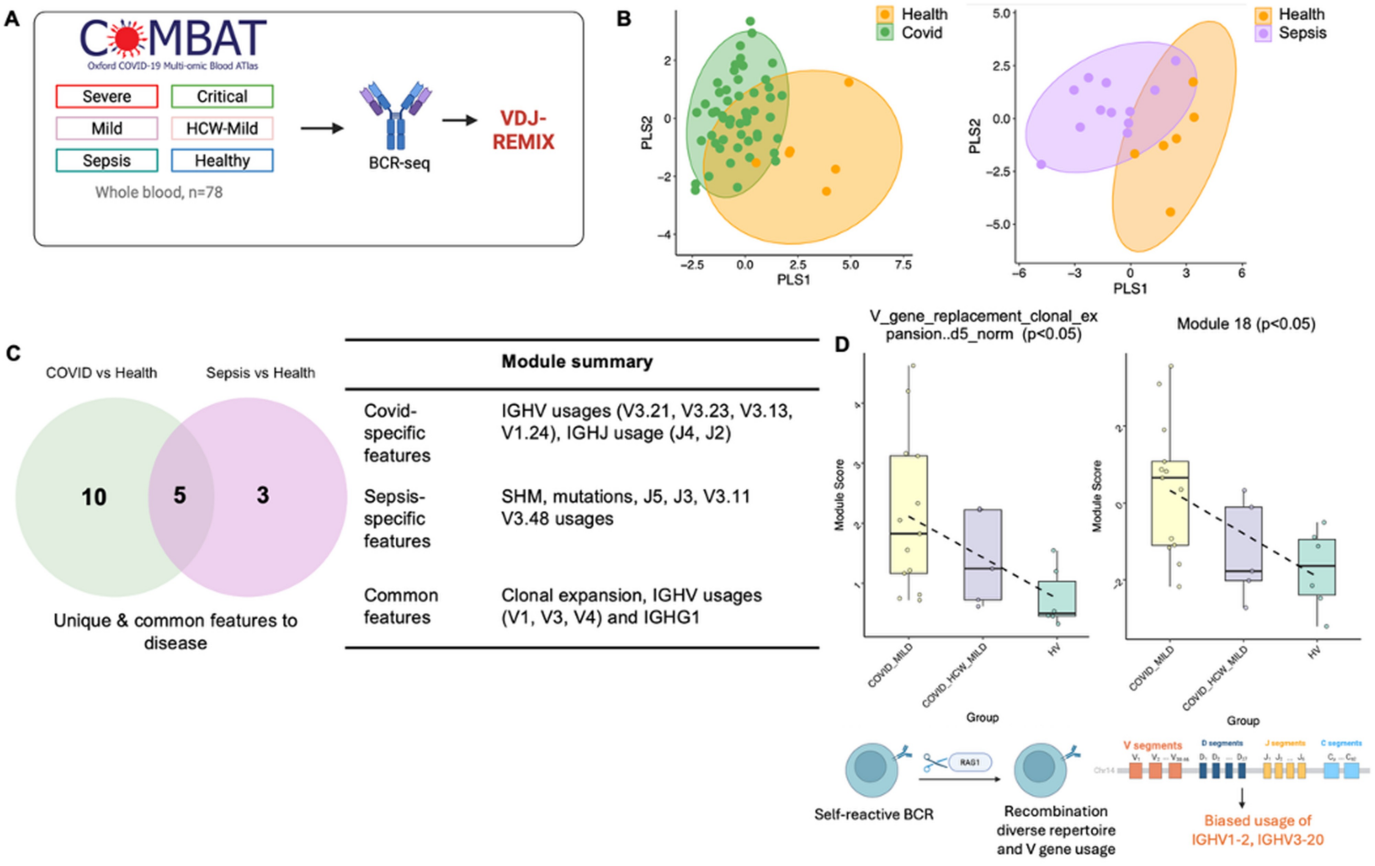
Dissecting shared and divergent BCR signatures between COVID-19 and non-COVID-19 sepsis. (A) Schematic of study design with cohort of COVID-19 patients of varying severity (mild, recovering, severe, critical), non-COVID-19 sepsis (severe and critical disease recruited prior to the pandemic) and healthy individuals. (B) Partial Least Squares Discriminant Analysis (PLS-DA) representation of sepsis versus health (right) and COVID-19 versus health (left) based on module scores and uncorrelated features. (C) Venn diagram of number of shared and unique modules classifying COVID-19 and sepsis from health, with their module meanings summarized in the table (right). (D) Boxplots and Jonckheere–Terpstra test across disease groups for (left) V-gene replacement and (right) Module 18 score, representing IGHV1-2 and IGHV3-20 across expanded and non-expanded clones (FDR <0.05).

Next, we sought to demonstrate the utility of VDJ-REMIX in resolving complex immune responses to acute infection. We analysed bulk BCR repertoire data from a cohort of 78 individuals, including patients with varying severities of COVID-19, patients with non-COVID-19 sepsis, and healthy controls (COMBAT Consortium 2022) (Fig. 3A). Applying VDJ-REMIX, we identified 29 modules of BCR features. An initial Partial Least Squares Discriminant Analysis (PLS-DA) of all modules showed that while both COVID-19 and sepsis patients could be distinguished from healthy controls, their own repertoire signatures exhibited overlap, underscoring the challenge of identifying disease-specific features (Fig. 3B).

PLS-DA stability scores were then used to distinguish disease states from healthy controls and to stratify modules into COVID-specific, sepsis-specific, and shared severe-infection signatures (Fig. 3C). Ten modules were uniquely dysregulated in COVID-19 versus health, characterized primarily by the biased usage of specific IGHV genes (including IGHV3-21, IGHV3-23) previously associated with SARS-CoV-2 responses (Wen *et al.* 2020, Zhang *et al.* 2020). Three modules were uniquely altered in sepsis versus health, distinguished by features of somatic hypermutation (SHM) and IGHJ gene. Critically, we identified five modules that were dysregulated in both conditions, representing a common ‘host response to severe infection’ signature. This shared signature was dominated by features of clonal expansion and the usage of IGHV1, IGHV4, and IGHG1 genes.

Furthermore, our analysis revealed modules that correlated with disease severity within the COVID-19 cohort. For instance, the module describing V-gene replacement and clonal expansion showed a significant decreasing monotonic function across mild COVID-19, recovering mild health care workers (HCW-Mild), and healthy volunteers (Jonckheere-Terpstra test for trend, FDR = 0.0487; Fig. 3D, left). V-gene replacement is a secondary genetic rearrangement in B and T cells where a pre-existing, rearranged V gene segment is replaced by an upstream germline V gene segment. This process can alter the antigen specificity of a BCR or TCR, and is often associated with autoreactivity (Zhang *et al.* 2003, Meng *et al.* 2014). Similarly, Module 18 representing V gene frequency for IGHV1-2 and IGHV3-20 across expanded and non-expanded clones also exhibited a significant trend across these groups (FDR = 0.0487; Fig. 3D, right). These results highlight the ability of VDJ-REMIX to not only distinguish between different acute infectious syndromes but also to identify quantitative immunological signatures that stratify patients by disease severity, providing a powerful tool for dissecting the complex B cell response to infection.

### 3.4 Shared and unique responses across cell populations in flow-sorted B cells

**Figure 4:**
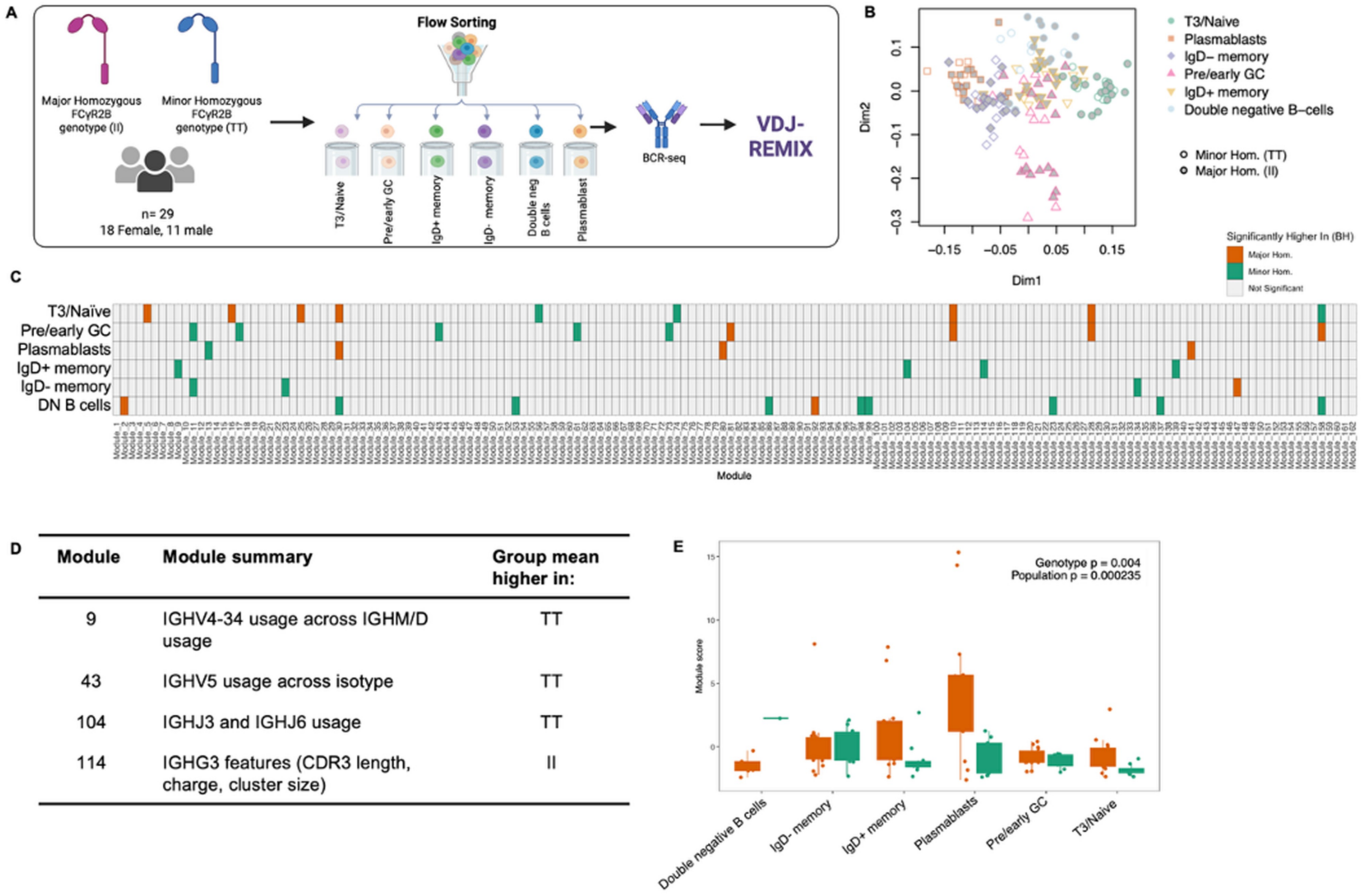
VDJ-REMIX identifies genotype-associated repertoire modules across B cell populations. (A) Schematic of experimental design. B cells from donors homozygous for the major (II) or minor (TT) FCGR2B allele (*n* = 29) were flow-sorted into six populations, subjected to bulk BCR sequencing, and analysed using VDJ-REMIX. (B) Low-dimensional projection of module eigengene profiles across samples, coloured by B cell population and shaped by genotype. (C) Heatmap of repertoire modules showing genotype-associated differences across B cell populations. Modules significantly higher in II (orange) or TT (green) individuals after multiple-testing correction are highlighted; non-significant modules are shown in white. (D) Representative genotype-associated modules with biological annotation. The genotype with higher mean module score is indicated. (E) Example module (Module 30: IGHV4-4 gene usage across isotypes) showing genotype- and population-dependent variation in module score. Boxplots show eigengene values across B cell populations stratified by genotype. *P* values for genotype and population effects were obtained using two-way ANOVA.

Next, we analysed flow-sorted B-cell populations stratified by FCGR2B genotype (Espéli *et al.* 2019). FCGR2B expression has been associated with autoimmune disease with the mutation involving replacement of isoleucine by a threonine at position 232, resulting in decreased inhibitory function and susceptibility to SLE (Siriboonrit *et al.* 2003). FCGR2B is also implicated in germinal center tolerance by preventing emergence of bystander autoreactive B cells (Giltiay *et al.* 2012). We applied VDJ-REMIX to BCR sequencing data generated from flow-sorted B cell populations from blood samples from 19 volunteers homozygous for isoleucine at position 232 in FCGR2B (hereafter referred to as I232 individuals), the common variant associated with normal FcγRIIB function, and 10 volunteers homozygous for the SLE-associated single-nucleotide polymorphism in FCGR2B (hereafter referred to T232 individuals) encoding a receptor that has markedly reduced inhibitory function (Fig. 4A). As a proof-of-principle, Principal Component Analysis (PCA) of module scores revealed significant variation in repertoire features across cell subtypes (Fig. 4B), e.g. distinguishing clearly T3/naïve and GC B cells occupying distinct regions of the embedding compared with plasmablasts and IgD${}^{-}$ memory B cells. In contrast, partial overlap between IgD${}^{+}$ memory, double-negative, and pre/early GC populations suggests shared repertoire characteristics consistent with transitional or developmentally related states. Together, these results indicate that module-level repertoire summaries capture both discrete cell-type specific signatures and continuous trajectories along B cell differentiation.

By correlating module scores with cell population, genotype, age group, and gender, we sought to determine if our unbiased approach could capture known and unknown biological roles of FCGR2B in regulating B cell tolerance. The previous study demonstrated that individuals with the autoimmunity-associated TT genotype exhibit increased frequencies of autoreactive IGHV4-34-expressing B cells, particularly within the naïve and pre-germinal centre (GC) compartments. Our analysis independently confirmed this key finding showing that Module 9, defined by features of unmutated, germline IGHV4-34 usage primarily within IgD+ B cells (a proxy for autoreactivity potential), was significantly enriched with the minor homozygous (TT) genotype (Fig. 4C). This data-driven result provides strong validation for our method, demonstrating its ability to rediscover subtle, biologically important signatures from complex repertoire data. A previous study reported increased IGHV5-family usage in SLE, consistent with our finding that Module 43 was elevated in TT individuals within pre/early GC B cells (Fraser *et al.* 2003).

Beyond this validation, our analysis uncovered novel, population-specific signatures associated with the FCGR2B genotype. For example, there is a preferential usage of IGHJ3 across isotypes (Module 98) in double-negative B cells and IGHJ5 (Module 104) in IgD+ memory B cells in individuals with the TT phenotype. Bias in gene usage based on FCGR2B mutation has not been reported before and again emphasizes the importance of capturing IGHJ usages across studies. Further, Module 114, which associated with features of IGHG3 and increased SHM, was enriched in the IgD+ memory B cells of individuals with the major allele II phenotype. This finding further confirms the role of FCGR2B in regulating germinal center activity and affinity maturation, demonstrating a more mature repertoire in the protective phenotype. The ability of VDJ-REMIX to resolve feature-level differences across specific cell subsets and genotypes underscores its utility in dissecting the granular mechanisms of immune regulation.

### 3.5 Application of VDJ-REMIX to single-cell multi-omics data reveals hidden immune programmes associated with survival-linked tumour microenvironments

**Figure 5:**
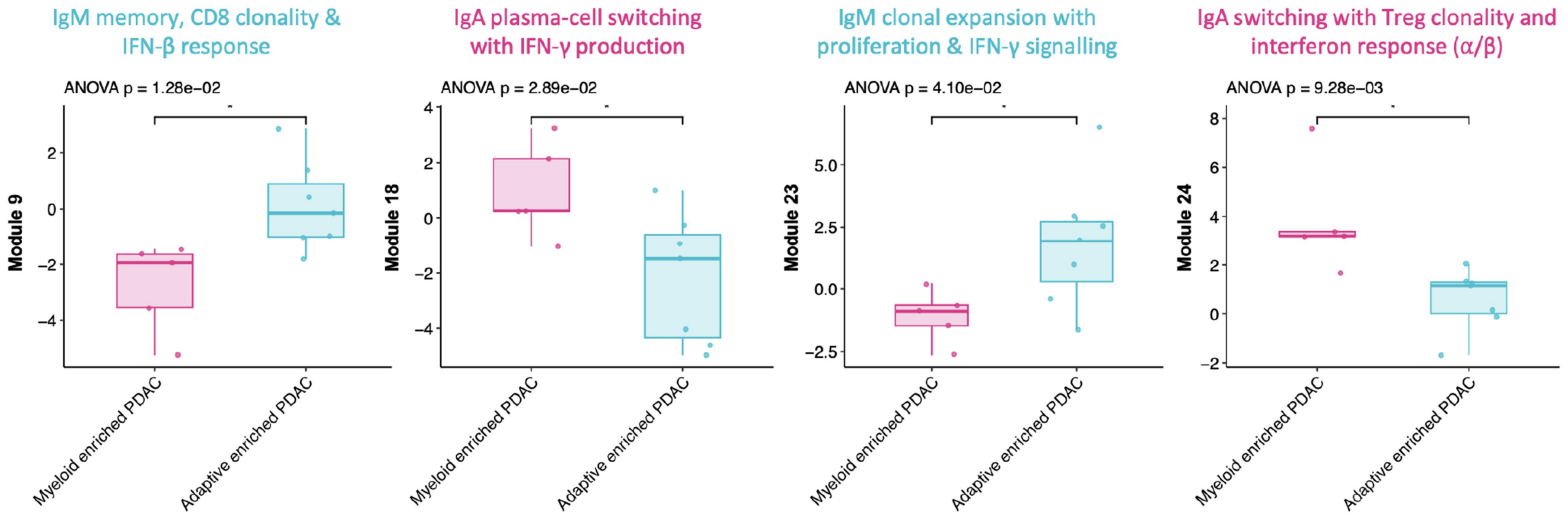
VDJ-REMIX applied to single-cell multi-omics PDAC data identifies modules associated with myeloid-enriched (*n* = 5) and adaptive-enriched (*n* = 7) tumour microenvironments linked to survival.

We next applied VDJ-REMIX to single-cell data to determine whether integrating repertoire and gene expression features across cell types could uncover previously hidden immune programmes associated with clinical outcomes. We analysed a single-cell multi-omics dataset of pancreatic ductal adenocarcinoma (PDAC), comprising tumour-infiltrating CD45^+^ immune cells from treatment-naïve patients (Sivakumar *et al.* 2025). This dataset integrates gene expression and paired BCR/TCR sequencing, previously revealing myeloid-enriched or adaptive-enriched tumour microenvironments associated with poorer or improved patient survival, respectively. By constructing an integrated pseudobulk matrix of 500 repertoire- and pathway-derived features across immune cell states, VDJ-REMIX identified patient-associated modules that recapitulated known differences in BCR isotype usage and clonality, while uncovering higher-order immune programmes that were not detected using conventional single-feature or univariate analyses. In Fig. 5, we show Module 18 captured an IgA-skewed plasma-cell program, whereas Module 9 linked IGHM-associated memory B cell features with expanded CD8 effector-memory clonality and interferon-β-response programs. Modules 23 and 24 extended this pattern by coupling clonality and isotype usage with proliferative, interferon-responsive and Treg-associated states. These findings suggest that the biological value of VDJ-REMIX lies not simply in detecting IgA/IgM or clonality differences *per se*, but in identifying coordinated immune architectures in which humoral remodeling is linked to cytotoxic, innate and regulatory programs. This suggests that repertoire features with similar univariate behaviour may reflect distinct underlying immune states, and that integrated module-level analysis may therefore provide a more informative readout of patient-group-specific immunobiology than any single repertoire metric alone.

### 3.6 Benchmarking: VDJ-REMIX improves interpretability over WGCNA and MOFA2

**Figure 6:**
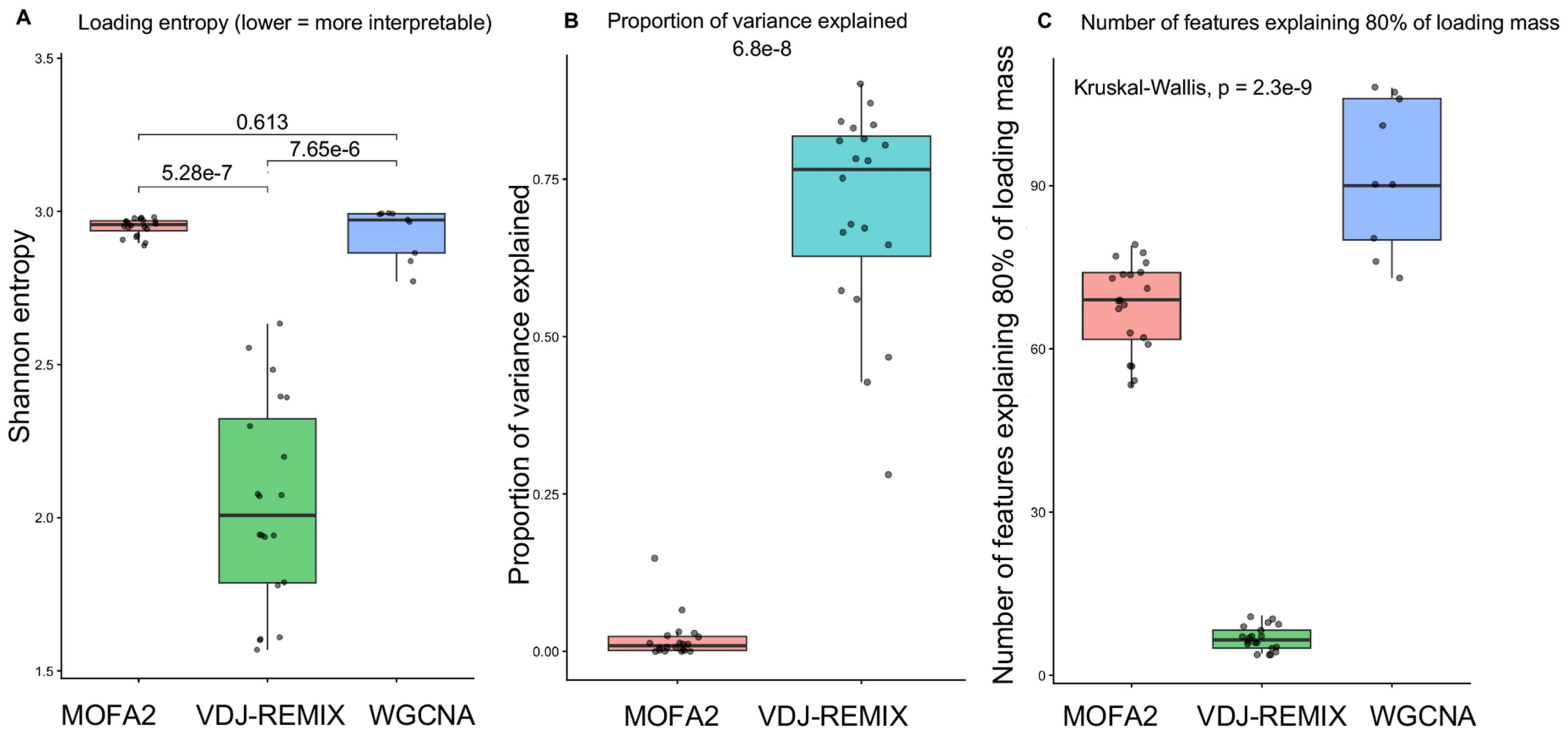
Benchmarking VDJ-REMIX compared to WGCNA and MOFA2. (A) Entropy of absolute feature loadings per latent dimension. (B) Proportion of within-dimension variance explained. (C) Number of features required to explain 80% of cumulative loading mass per latent dimension. Each point represents one latent dimension; boxplots indicate median and interquartile range.

We benchmarked VDJ-REMIX against WGCNA and MOFA2 using the autoimmune dataset (Fig. 6). Interpretability was assessed using three metrics: entropy of feature loadings (signal concentration), proportion of variance explained, and the number of features required to capture 80% of loading mass. Together, these quantify whether signal is captured by a small, coherent set of features or distributed across many. Predictive performance for disease and therapy was also evaluated using identical preprocessing.

Across all interpretability metrics, VDJ-REMIX outperformed both MOFA2 (Argelaguet *et al.* 2020) and WGCNA. It generated lower entropy modules, explained a greater proportion of variance per dimension, and required fewer features to capture the majority of signal, indicating that its modules represent more compact and biologically coherent units.

Critically, these gains in interpretability did not compromise predictive performance. In a binary classification task distinguishing SLE from AAV, evaluated using 50 repeated cross-validation folds, all methods achieved comparable accuracy (VDJ-REMIX: 0.8007 ± 0.0650; WGCNA: 0.7986 ± 0.0702; MOFA2: 0.8007 ± 0.0673). The small differences relative to fold-to-fold variability indicate that VDJ-REMIX achieves improved interpretability without loss of predictive power.

## 4 Discussion

VDJ-REMIX provides a scalable and unified framework for the unsupervized analysis of immune repertoire data, addressing a key limitation in AIRR-seq by resolving high-dimensional feature spaces into biologically coherent modules. This modular structure reveals axes of immunological variation that are not accessible through conventional feature-level analyses, overcoming both statistical and interpretive barriers posed by high-dimensional datasets.

Across diverse cohorts, VDJ-REMIX disentangles both shared and disease-specific immune signatures. While modules recapitulate known biology, such as IGHV4-34-associated autoreactivity in SLE and CDR3 length shifts in Crohn’s disease, they also uncover previously unrecognized features, including isotype-specific IGHJ4 dynamics in SLE and AAV. These findings demonstrate that modular repertoire analysis functions not only as a dimensionality reduction strategy, but as a discovery framework for identifying new immunological axes.

Importantly, VDJ-REMIX captures dynamic and context-specific immune processes. Module trajectories reflect distinct therapeutic mechanisms, with MMF suppressing proliferative, autoimmunity-associated programmes and RTX driving naïve B cell repopulation. In acute infection, the method resolves both disease-specific and shared host-response signatures, identifying convergent clonal expansion programmes across COVID-19 and non-COVID-19 sepsis.

A key advance is the extension of VDJ-REMIX to single-cell multi-omics data. By integrating repertoire and transcriptomic features into pseudobulk representations, we show that sparse single-cell inputs can be organized into coherent immune modules. In PDAC, this revealed that canonical repertoire signals, such as IgA/IgM usage and clonality, are embedded within coordinated cytotoxic, interferon-driven, and regulatory programmes. Notably, features with similar univariate behaviour segregated into distinct modules with different cellular and pathway associations, indicating that they arise from fundamentally different immune states. This demonstrates that VDJ-REMIX uncovers higher-order immune organization linking humoral and cellular compartments, which is not apparent from repertoire or transcriptomic analyses alone.

Unlike generic factorization approaches that optimize variance or reconstruction error, VDJ-REMIX is explicitly designed to identify biologically coherent modules. This results in compact, interpretable latent dimensions that concentrate signal within a small set of meaningful features, in contrast to the more diffuse representations produced by MOFA2 (Argelaguet *et al.* 2020) and WGCNA. This enhanced interpretability enables direct biological insight and facilitates hypothesis generation in complex datasets.

Despite these advances, module composition remains dependent on input features and cohort structure, and the inferred relationships are correlative. Experimental validation will be required to establish mechanistic and translational relevance. Future development should also focus on improving accessibility and cross-platform compatibility.

In summary, VDJ-REMIX enables a systems-level view of immune repertoires, identifying coordinated immune programmes that underlie disease heterogeneity. By moving beyond single-feature analyses, it provides a framework for discovering clinically relevant and mechanistically informative immune states across bulk and single-cell datasets.

## Supplementary Material

btag326_Supplementary_Data

## Data Availability

The VDJ-REMIX software is implemented as an R package and is publicly available on GitHub (https://github.com/Bashford-Rogers-lab/vdjremix) under the GNU General Public License v3.0 (GPL-3.0). Processed feature matrices and the R code used to reproduce the figures in this study are also available on GitHub.
