# Supplementary material for: VDJ-REMIX: REpertoire Module Identification and eXploration
Source: Bioinformatics. 2026 Jul 7;42(Suppl 1):btag326. doi: 10.1093/bioinformatics/btag326 (PMC13340219; doi:10.1093/bioinformatics/btag326)
Supplement: btag326_Supplementary_Data [file btag326_supplementary_data.zip › Amin.276.suppl.document.pdf]

VDJ-REMIX: REpertoire Module Identification and eXploration

Sakina Amin, Lauren Overend, Felicia Tucci, Bo Sun, Justin Whalley, Michael Dustin, Julian Knight, Rachael Bashford-Rogers

Supplementary Methods

The VDJ-REMIX pipeline

We developed VDJ-REMIX, a repertoire module reduction framework to summarise high-dimensional repertoire metrics into interpretable modules.VDJ-REMIX refactors the core ideas of weighted correlation network analysis (WGCNA) to make module discovery robust for adaptive immune receptor repertoire (AIRR) feature matrices, which differ fundamentally from transcriptomic matrices in size (hundreds rather than tens of thousands of variables), data type (mixtures of proportions, ratios, diversity indices and gene-usage metrics), and structured missingness driven by biology and sequencing depth. In contrast to default WGCNA pipelines that assume dense, approximately continuous expression measurements, VDJ-REMIX introduces AIRR-specific preprocessing and inference steps: (i) feature filtering to remove sparse or low-information metrics that generate unstable correlations, (ii) imputation benchmarking and robustness filtering to ensure retained features remain imputable under the missingness regimes observed in the cohort, and (iii) a subsampling-based correlation estimator that stabilizes feature–feature correlations when overlap varies across feature pairs. Modules are then summarized by oriented PC1 "eigengenes" to produce compact, biologically interpretable module scores for downstream association and prediction. Compared with factorization-based approaches such as PCA and MOFA2, which optimize variance explained or reconstruction error and can distribute signal across many features, VDJ-REMIX explicitly constructs coherent groups of co-varying repertoire metrics, improving interpretability while retaining comparable predictive performance in benchmarking.

Repertoire feature definition and biological interpretation: Immune repertoire summary features were derived from annotated BCR sequencing data using a custom preprocessing pipeline, capturing complementary aspects of clonal architecture, gene usage, and sequence diversification. Clonality-related metrics were computed by clustering sequences into clonal groups based on sequence similarity, reflecting expansion from a common ancestor; in B cell repertoires, clonal members may differ at the nucleotide level due to somatic hypermutation, enabling quantification of intra-clonal diversification. Diversity was quantified using multiple complementary indices adapted from ecological theory, including richness- and inequality-based measures, to capture both the breadth and dominance structure of the repertoire. Gene usage features summarized the frequency distributions of V, (D), J, and constant region genes, which are known to reflect antigen selection, autoreactivity, and effector function. Sequence-level properties such as CDR3 length distributions and physicochemical characteristics were computed to capture variation in antigen recognition potential. For BCRs, somatic hypermutation frequency was quantified by comparison to germline references, with mutation burden serving as a proxy for antigen-driven selection and affinity maturation. Gene usage and isotype features are percentages. Relative class switching are normalized values (instances/ threshold subsample depth) which is dataset dependent. Module structure was broadly consistent as the matrices are scaled prior to eigengene calculation providing a practical balance between interpretability and stability. Collectively, these features provide a biologically interpretable, high-dimensional representation of adaptive immune repertoires that integrates clonal structure, diversity, and functional specialization.

Single-cell feature matrix generation

Pathway activity analysis was performed in Seurat on the integrated PDAC single-cell dataset. Scaled gene-expression matrices were used to calculate module scores for selected biological pathways, including apoptosis, proliferation, hypoxia, and interferon signalling, using curated MSigDB gene sets and AddModuleScore. Median pathway scores were computed per cell type per sample, requiring at least five cells per group, and were integrated with immune repertoire features, including BCR/TCR clonality metrics (clone-size distributions, intra- and inter-subset clonality, and clonal expansion measures), V/J gene usage, and isotype distributions. The combined matrix was filtered to retain features present in at least 19 samples with sufficient variability and was analysed using VDJ-REMIX to identify co-varying feature modules. Associations between modules and clinical groups were assessed using MANOVA to identify pathway–clonality relationships linked to disease phenotypes. Code used to generate these metrics is provided in the single-cell analysis scripts and in the linked repository (`https://github.com/rbr1/scIsoTyper/blob/main/scClonetoire.R`.)

Feature filtering

To improve statistical robustness and minimise spurious correlations arising from sparse or low-information features, the repertoire feature matrix was filtered prior to network construction and imputation. Features were excluded if they exhibited high missingness (>40% NA values across samples) or low variability (fewer than 8 unique observed values across the cohort). These criteria remove features with limited analytical value, thereby reducing noise and improving the stability of downstream correlation-based module detection. Importantly, the resulting reduction in feature number reflects quality control rather than loss of biologically meaningful information, and enhances the robustness and interpretability of the inferred modules. In VDJ-REMIX, missingness is summarised per sample to characterise the maximum missingness burden observed across individuals, which was subsequently used during imputation robustness filtering (below).

Table 1. Feature retention after preprocessing and filtering across datasets. Sample counts reflect analysed sample-level profiles; for the flow-sorted cohort, multiple samples were derived per donor across sorted B cell populations.

Dataset	Samples (n)	Retained features (n)
Autoimmune	237	225
Flow-sorted	155	1809
COVID/Sepsis	78	450

Missing value imputation in high-coverage features

Missing repertoire values can arise from biological absence (for example, absent isotypes or undetected V/J usage) and from sampling depth limitations. Because PCA-based eigengene construction requires a complete matrix, we used a two-stage strategy: (i) benchmarking and robustness filtering, followed by (ii) final imputation for downstream eigengene calculation and visualisation.

Imputation benchmarking by controlled missingness seeding

We first derived a complete-case "ground truth" matrix by restricting to samples with no missing values across the retained feature set (complete.cases). Controlled missingness was then seeded per feature by applying a missingness operator at frequency $f \in \{0.05, 0.10, \ldots, 0.50\}$, generating verification matrices $X^{(f)}$. Missingness patterns were visualised using missingness maps and missingness pattern summaries.

We benchmarked multiple imputation strategies (including mean/median imputation, random replacement, kNN-based imputation, missMDA PCA-based methods, additive regression-based imputation, and Random Forest imputation). For each method, we quantified reconstruction accuracy using:

1. Global NRMSE, computed over entries that were artificially masked, using a normalised error metric of the form

$$NRMSE(X, \widehat{X}) = \frac{\sqrt{\frac{1}{|\Omega|}\sum_{(i,j)\in\Omega}(X_{ij} - \widehat{X}_{ij})^2}}{\sigma(X_\Omega)},$$

where Ω is the set of artificially masked entries and $\sigma(\cdot)$ denotes the standard deviation over the masked ground-truth values.

2. Feature-wise reconstruction error, summarised for each feature $j$as

$$RMSE_j = \frac{\sqrt{\sum_{i=1}^{n}(X_{ij} - \widehat{X}_{ij})^2}}{n},$$

computed after reshaping imputed matrices into long format and aggregating errors per feature.

Across missingness levels, Random Forest imputation (missForest; maxiter = 20) consistently showed the most favourable performance and was therefore selected for final imputation.

Robustness filtering based on observed missingness

To avoid retaining features whose imputation becomes unreliable at missingness levels that are present in the dataset, we applied an additional robustness filter using missForest feature-wise error profiles. Let m_{sample} be the maximum fraction of missing values observed in any sample in the real (non-seeded) dataset. We identified features for which the missForest feature-wise error exceeded a threshold (rSME > 0.5) at seeded missingness frequencies $f < m_{\text{sample}}$. Such features were removed because their imputation accuracy deteriorated before reaching the missingness regime already observed in the cohort.

A complementary check was also performed by comparing each feature's observed missingness proportion m_{feature} against the earliest seeded missingness frequency at which the feature exceeded rSME > 0.5. Features were removed if the method became unreliable at or below that feature's missingness burden.

Final imputation for downstream eigengene computation

After robustness filtering, the full (non-complete case) dataset was imputed using missForest (maxiter = 20). The out-of-bag NRMSE reported by missForest was recorded as a summary measure of imputation quality. Distributions of observed versus imputed values were visually inspected per feature to ensure imputation did not introduce obvious distributional artefacts. The final imputed matrix was then scaled (z-scored) feature-wise prior to PCA-based eigengene construction.

Robust correlation estimation by subsampling on non-imputed data

Network construction was performed on the filtered, non-imputed matrix to minimise dependence of topology on imputed values. For each feature pair (i, j), let S_{ij} be the set of samples where both features are observed (pairwise complete overlap). We first determined a global subsampling depth

$$k = \left\lfloor 0.8 \times \min_{i<j} \mid S_{ij} \mid \right\rfloor,$$

so that each pairwise correlation was estimated using a common sample size while preserving stochastic variation across subsamples.

For each feature pair, we performed $B = 10,000$ subsampling iterations. At iteration b, we drew a random subset $T_{ij}^{(b)} \subseteq S_{ij}$with $\mid T_{ij}^{(b)} \mid= k$(sampling without replacement) and computed a Pearson correlation. The robust correlation estimate was then

$$\widehat{r}_{ij} = \frac{1}{B}\sum_{b=1}^{B} cor\left(X_i[T_{ij}^{(b)}], X_j[T_{ij}^{(b)}]\right).$$

The resulting robust correlation matrix $\widehat{R}$was stored for downstream clustering and visualisation.

Distance matrix definition

To preserve correlation sign (so that anticorrelated metrics are considered more distant than positively correlated metrics), we defined a signed distance matrix

$$D_{ij} = \frac{1 - \widehat{r}_{ij}}{2}.$$

For diagnostic purposes, we also evaluated an alternative absolute-correlation distance $D_{ij}^* = 1- \mid \widehat{r}_{ij} \mid$, but proceeded with the signed definition to retain information on anticorrelation structure.

Linkage selection by agglomerative coefficient

We compared multiple hierarchical clustering linkage criteria (average, single, complete, weighted, and Ward) by computing the agglomerative coefficient (AC) for each method. The linkage method achieving the maximal AC was selected for the final dendrogram construction. When Ward linkage was selected by AC, Ward.D2 was used for hclust compatibility.

Determining the number of clusters

To select an appropriate module resolution, we used internal cluster validation indices implemented via NbClust (Dunn, Silhouette, McClain, C-index, and Frey). The search range depended on receptor type; for BCR analyses, we typically evaluated a range spanning 10–120 clusters (bounded by the matrix dimension). The optimal number of clusters was chosen by a majority-rule consensus across indices, with ties resolved by selecting the larger cluster number to avoid overly coarse partitions.

Module detection and handling of unassigned features

We partitioned the dendrogram using two complementary strategies:

1. Fixed-height cutting using the selected number of clusters K.
2. Dynamic tree cutting using cutreeDynamic with hybrid mode, high sensitivity (deepSplit = TRUE), and a minimum module size of 10 features (minClusterSize = 10).

If dynamic cutting yielded a smaller and more stable partition than fixed-height cutting, we proceeded with the dynamic assignment. Features labelled as unassigned (cluster label 0) were removed from downstream eigengene PCA and stored separately for diagnostic review.

Module eigengenes and driver feature definition

Modules were summarised using module eigengenes computed on the scaled, missForest-imputed matrix.

Eigengene construction

For each module $k$containing $p_k \geq 2$features, we performed PCA (prcomp) on the module's scaled feature submatrix and defined the module eigengene as the first principal component score vector:

$$ME_k = PC1(X_k).$$

Because PCA component signs are arbitrary, we oriented each eigengene to align with the module's mean feature vector. Let $overlinex_k$be the per-sample mean across features in module k. If $cor(ME_k,$ $overlinex_k) < 0$, we multiplied ME_k and the corresponding PC1 loading vector by $-1$to ensure that higher eigengene values correspond to higher module feature abundance on average.

Modules containing a single feature ($p_k = 1$) were not subjected to PCA; instead, the scaled feature itself was carried forward as the module summary and labelled with a "Module_feature" prefix to distinguish it from PCA-derived eigengenes.

Driver feature definition

For each multi-feature module k, we extracted PC1 loadings $l_{jk}$for each feature $j$in the module. We defined driver features as those whose absolute loading exceeded a uniform-chance threshold:

$$| l_{jk} | \geq \sqrt{\frac{1}{p_k}}.$$

This threshold marks features contributing more strongly to PC1 than expected if loadings were evenly distributed across features.

We additionally quantified module membership (also referred to as kME) as the correlation between each feature and its module eigengene:

$$kME_{jk} = cor(X_j, ME_k).$$

Loadings and kME statistics were retained for interpretation and for highlighting key drivers within modules.

Visualisation and interpretability

VDJ-REMIX generates standardised visual outputs for module interpretation and quality control, including low-dimensional embeddings of module eigengenes, heatmaps of module–phenotype associations, and stratified distributions of individual module scores. These plots are generated directly from pipeline outputs and are intended to facilitate interpretation of module behaviour across samples and conditions.

Supplementary Figures

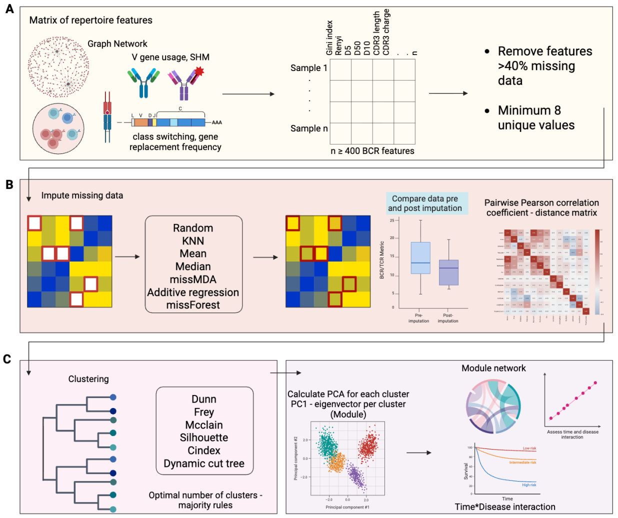

Supplementary Figure 1. Detailed preprocessing and module construction pipeline in VDJ-REMIX.

Table 1 – Module and features associated with immune-mediated diseases

"Feature ""Cluster"""
"mean_CDR_FWR_ratio..IGHA1 ""Unassigned"""
"mean_CDR_FWR_ratio..IGHD_IGHM_mutated ""Unassigned"""
"mean_CDR_FWR_ratio..IGHG2 ""Unassigned"""
"mean_CDR_FWR_ratio..IGHM ""Unassigned"""
"Mean_SHM_per_BCR..Class_switched ""1"""
"Mean_SHM_per_BCR..IGHA1 ""1"""
"Mean_SHM_per_BCR..IGHG2 ""1"""
"Mean_SHM_per_BCR..IGHG3 ""1"""
"Mean_SHM_per_BCR..expanded ""1"""
"FWR3_mm..IGHA1 ""1"""
"FWR3_mm..IGHG2 ""1"""
"mean_CDR_mm_per_BCR..IGHA1 ""1"""
"mean_CDR_mm_per_BCR..IGHG2 ""1"""
"mean_FWR_mm_per_BCR..IGHA1 ""1"""
"mean_FWR_mm_per_BCR..IGHG2 ""1"""
"Mean_mutations_per_BCR..IGHA1 ""1"""
"Mean_mutations_per_BCR..IGHG2 ""1"""
"Mean_mutations_per_BCR..IGHG3 ""1"""
"D5..IGHG3.18 ""2"""
"D5..IGHM.03 ""2"""
"D5..all ""2"""
"D10..IGHM.03 ""2"""
"D10..all ""2"""
"D50..IGHM.03 ""2"""
"D50..all ""2"""
"V_gene_replacement_clonal_expansion..d5_secondary ""2"""
"V_gene_replacement_clonal_expansion..mean_clone_size_norm ""2"""
"V_gene_replacement_clonal_expansion..mean_clone_size_secondary ""2"""
"Relative_class_switching_normalised..IGHA1.IGHD.M ""2"""
"Relative_class_switching_normalised..IGHA1.IGHG2 ""2"""
"Relative_class_switching_normalised..IGHD.M.IGHG2 ""2"""
"mean_CDR3_charge..IGHA1 ""3"""
"mean_CDR3_charge..IGHG2 ""3"""
"mean_CDR3_charge..IGHM ""3"""
"J_gene_freq_by_uniq_VDJ_Class_switched..IGHJ4 ""3"""
"J_gene_freq_by_uniq_VDJ_IGHA1..IGHJ4 ""3"""
"J_gene_freq_by_uniq_VDJ_IGHD..IGHJ4 ""3"""
"J_gene_freq_by_uniq_VDJ_IGHD.IGHM_mutated..IGHJ4 ""3"""
"J_gene_freq_by_uniq_VDJ_IGHG2..IGHJ4 ""3"""
"J_gene_freq_by_uniq_VDJ_IGHG3..IGHJ4 ""3"""
"J_gene_freq_by_uniq_VDJ_IGHM..IGHJ4 ""3"""
"J_gene_freq_by_uniq_VDJ_expanded..IGHJ4 ""3"""
"J_gene_freq_by_uniq_VDJ_unexpanded..IGHJ4 ""3"""
"Mean_SHM_per_BCR..IGHD ""4"""
"Mean_SHM_per_BCR..IGHM ""4"""
"Mean_SHM_per_BCR..unexpanded ""4"""
"FWR3_mm..IGHD_IGHM_mutated ""4"""

"FWR3_mm..IGHM ""4"""
"mean_CDR_mm_per_BCR..IGHD_IGHM_mutated ""4"""
"mean_CDR_mm_per_BCR..IGHM ""4"""
"mean_FWR_mm_per_BCR..IGHD_IGHM_mutated ""4"""
"mean_FWR_mm_per_BCR..IGHM ""4"""
"Mean_mutations_per_BCR..IGHD ""4"""
"Mean_mutations_per_BCR..IGHD_IGHM_mutated ""4"""
"Mean_mutations_per_BCR..IGHM ""4"""
"V_gene_replacement_clonal_expansion..d5_norm ""5"""
"J_gene_freq_by_uniq_VDJ_Class_switched..IGHJ1 ""5"""
"J_gene_freq_by_uniq_VDJ_IGHA1..IGHJ1 ""5"""
"J_gene_freq_by_uniq_VDJ_IGHD..IGHJ1 ""5"""
"J_gene_freq_by_uniq_VDJ_IGHD.IGHM_mutated..IGHJ1 ""5"""
"J_gene_freq_by_uniq_VDJ_IGHE..IGHJ1 ""5"""
"J_gene_freq_by_uniq_VDJ_IGHG2..IGHJ1 ""5"""
"J_gene_freq_by_uniq_VDJ_IGHG3..IGHJ1 ""5"""
"J_gene_freq_by_uniq_VDJ_IGHM..IGHJ1 ""5"""
"J_gene_freq_by_uniq_VDJ_expanded..IGHJ1 ""5"""
"J_gene_freq_by_uniq_VDJ_unexpanded..IGHJ1 ""5"""
"V4_34_AVY_unmut..IGHA1 ""6"""
"V4_34_AVY_unmut..IGHG2 ""6"""
"V4_34_AVY_unmut..IGHM ""6"""
"V4_34_NHS_unmut..IGHA1 ""6"""
"V4_34_NHS_unmut..IGHG2 ""6"""
"V4_34_NHS_unmut..IGHM ""6"""
"V4_34_AVY_NHS_unmut..IGHA1 ""6"""
"V4_34_AVY_NHS_unmut..IGHG2 ""6"""
"V4_34_AVY_NHS_unmut..IGHM ""6"""
"V4_34_AVY_NHS_unmut..IGHDM ""6"""
"V4_34_AVY_NHS_unmut..class_switched ""6"""
"Percentage_unique_BCRs_per_isotype..IGHD.IGHM_mutated ""7"""
"Percentage_unique_BCRs_per_isotype..IGHD.IGHM_unmutated ""7"""
"Percentage_unique_BCRs_per_isotype_group..IGHD ""7"""
"Percentage_unique_BCRs_per_isotype_group..IGHM ""7"""
"Mean_CDR3_lengths..IGHA1 ""7"""
"Mean_CDR3_lengths..IGHG2 ""7"""
"Mean_CDR3_lengths..IGHM ""7"""
"Relative_class_switching_normalised..IGHA1.IGHA2 ""7"""
"Relative_class_switching_normalised..IGHA2.IGHD.M ""7"""
"Relative_class_switching_normalised..IGHA2.IGHG2 ""7"""
"J_gene_freq_by_uniq_VDJ_Class_switched..IGHJ6 ""8"""
"J_gene_freq_by_uniq_VDJ_IGHA1..IGHJ6 ""8"""
"J_gene_freq_by_uniq_VDJ_IGHD..IGHJ6 ""8"""
"J_gene_freq_by_uniq_VDJ_IGHE..IGHJ6 ""8"""
"J_gene_freq_by_uniq_VDJ_IGHG2..IGHJ6 ""8"""
"J_gene_freq_by_uniq_VDJ_IGHG3..IGHJ6 ""8"""
"J_gene_freq_by_uniq_VDJ_IGHM..IGHJ6 ""8"""
"J_gene_freq_by_uniq_VDJ_expanded..IGHJ6 ""8"""
"Percentage_unmutated..Class_switched ""9"""

"Percentage_unmutated..IGHA1 ""9"""
"Percentage_unmutated..IGHD ""9"""
"Percentage_unmutated..IGHG2 ""9"""
"Percentage_unmutated..IGHG3 ""9"""
"Percentage_unmutated..IGHM ""9"""
"Percentage_unmutated..expanded ""9"""
"Percentage_unmutated..unexpanded ""9"""
"Relative_class_switching_absolute..IGHA1.01.IGHG2.03 ""10"""
"Relative_class_switching_normalised..IGHA1.IGHE ""10"""
"Relative_class_switching_normalised..IGHA1.IGHG3 ""10"""
"Relative_class_switching_normalised..IGHD.M.IGHE ""10"""
"Relative_class_switching_normalised..IGHD.M.IGHG3 ""10"""
"Relative_class_switching_normalised..IGHE.IGHG2 ""10"""
"Relative_class_switching_normalised..IGHE.IGHG3 ""10"""
"Relative_class_switching_normalised..IGHG2.IGHG3 ""10"""
"J_gene_freq_by_uniq_VDJ_Class_switched..IGHJ5 ""11"""
"J_gene_freq_by_uniq_VDJ_IGHA1..IGHJ5 ""11"""
"J_gene_freq_by_uniq_VDJ_IGHD..IGHJ5 ""11"""
"J_gene_freq_by_uniq_VDJ_IGHG2..IGHJ5 ""11"""
"J_gene_freq_by_uniq_VDJ_IGHG3..IGHJ5 ""11"""
"J_gene_freq_by_uniq_VDJ_IGHM..IGHJ5 ""11"""
"J_gene_freq_by_uniq_VDJ_expanded..IGHJ5 ""11"""
"J_gene_freq_by_uniq_VDJ_Class_switched..IGHJ3 ""12"""
"J_gene_freq_by_uniq_VDJ_IGHA1..IGHJ3 ""12"""
"J_gene_freq_by_uniq_VDJ_IGHD..IGHJ3 ""12"""
"J_gene_freq_by_uniq_VDJ_IGHG2..IGHJ3 ""12"""
"J_gene_freq_by_uniq_VDJ_IGHG3..IGHJ3 ""12"""
"J_gene_freq_by_uniq_VDJ_IGHM..IGHJ3 ""12"""
"J_gene_freq_by_uniq_VDJ_expanded..IGHJ3 ""12"""
"J_gene_freq_by_uniq_VDJ_Class_switched..IGHJ2 ""13"""
"J_gene_freq_by_uniq_VDJ_IGHA1..IGHJ2 ""13"""
"J_gene_freq_by_uniq_VDJ_IGHD..IGHJ2 ""13"""
"J_gene_freq_by_uniq_VDJ_IGHG2..IGHJ2 ""13"""
"J_gene_freq_by_uniq_VDJ_IGHG3..IGHJ2 ""13"""
"J_gene_freq_by_uniq_VDJ_IGHM..IGHJ2 ""13"""
"J_gene_freq_by_uniq_VDJ_expanded..IGHJ2 ""13"""
"J_gene_freq_by_uniq_VDJ_IGHD.IGHM_mutated..IGHJ5 ""14"""
"J_gene_freq_by_uniq_VDJ_IGHD.IGHM_mutated..IGHJ6 ""14"""
"J_gene_freq_by_uniq_VDJ_IGHD.IGHM_unmutated..IGHJ4 ""14"""
"J_gene_freq_by_uniq_VDJ_IGHD.IGHM_unmutated..IGHJ5 ""14"""
"J_gene_freq_by_uniq_VDJ_IGHD.IGHM_unmutated..IGHJ6 ""14"""
"J_gene_freq_by_uniq_VDJ_unexpanded..IGHJ5 ""14"""
"J_gene_freq_by_uniq_VDJ_unexpanded..IGHJ6 ""14"""
"Relative_class_switching_absolute..IGHA1.01.IGHG3.18 ""15"""
"Relative_class_switching_absolute..IGHD.01.IGHG3.18 ""15"""
"Relative_class_switching_absolute..IGHG2.03.IGHG3.17 ""15"""
"Relative_class_switching_absolute..IGHG2.03.IGHG3.18 ""15"""
"Relative_class_switching_absolute..IGHG3.17.IGHG3.18 ""15"""
"Relative_class_switching_absolute..IGHG3.18.IGHG4.01 ""15"""

"J_gene_freq_by_uniq_VDJ_IGHD.IGHM_mutated..IGHJ2 ""16"""
"J_gene_freq_by_uniq_VDJ_IGHD.IGHM_mutated..IGHJ3 ""16"""
"J_gene_freq_by_uniq_VDJ_IGHD.IGHM_unmutated..IGHJ2 ""16"""
"J_gene_freq_by_uniq_VDJ_IGHD.IGHM_unmutated..IGHJ3 ""16"""
"J_gene_freq_by_uniq_VDJ_unexpanded..IGHJ2 ""16"""
"J_gene_freq_by_uniq_VDJ_unexpanded..IGHJ3 ""16"""
"D5..IGHA1.01 ""17"""
"D5..IGHG2.03 ""17"""
"D10..IGHA1.01 ""17"""
"D10..IGHG2.03 ""17"""
"D50..IGHA1.01 ""17"""
"Percentage_unique_BCRs_per_isotype..Class_switched ""18"""
"Percentage_unique_BCRs_per_isotype_group..IGHA1 ""18"""
"Percentage_unique_BCRs_per_isotype_group..IGHG2 ""18"""
"Relative_class_switching_absolute..IGHA1.01.IGHA2.01 ""19"""
"Relative_class_switching_absolute..IGHA1.01.IGHE.02 ""19"""
"Relative_class_switching_absolute..IGHA1.01.IGHG4.01 ""19"""
"Relative_class_switching_absolute..IGHE.02.IGHG2.03 ""19"""
"Relative_class_switching_absolute..IGHG2.03.IGHG4.01 ""19"""
"Relative_class_switching_absolute..IGHA1.01.IGHG1.03 ""20"""
"Relative_class_switching_absolute..IGHG1.03.IGHG2.03 ""20"""
"Relative_class_switching_normalised..IGHA1.IGHG1 ""20"""
"Relative_class_switching_normalised..IGHD.M.IGHG1 ""20"""
"Relative_class_switching_normalised..IGHG1.IGHG2 ""20"""

Table 2 – Module and features associated with COMBAT data

Feature "Cluster"
Cluster_Gini_Index..all "1"
mean_vertex_size..all "1"
Percentage_max_cluster_size..IGHA1 "1"
Percentage_max_cluster_size..IGHA2 "1"
Percentage_max_cluster_size..IGHD "1"
Percentage_max_cluster_size..IGHG3 "1"
Percentage_max_cluster_size..IGHM "1"
Percentage_max_cluster_size..all "1"
Percentage_max_vertex_size..IGHA1 "1"
Percentage_max_vertex_size..IGHA2 "1"
Percentage_max_vertex_size..IGHD "1"
Percentage_max_vertex_size..IGHG1 "1"
Percentage_max_vertex_size..IGHG3 "1"
Percentage_max_vertex_size..IGHM "1"
Percentage_max_vertex_size..all "1"
Percentage_unique_BCRs_per_isotype..Class_switched "1"
D5..all "1"
D10..all "1"
D50..all "1"

V_gene_replacement_clonal_expansion..d5_secondary "1"
J_gene_freq_by_uniq_VDJ_Class_switched..IGHJ6 "1"
J_gene_freq_by_uniq_VDJ_IGHA1..IGHJ6 "1"
J_gene_freq_by_uniq_VDJ_IGHA2..IGHJ6 "1"
J_gene_freq_by_uniq_VDJ_IGHD..IGHJ6 "1"
J_gene_freq_by_uniq_VDJ_IGHG1..IGHJ6 "1"
J_gene_freq_by_uniq_VDJ_IGHG2..IGHJ6 "1"
J_gene_freq_by_uniq_VDJ_IGHG3..IGHJ6 "1"
J_gene_freq_by_uniq_VDJ_IGHM..IGHJ6 "1"
J_gene_freq_by_uniq_VDJ_expanded..IGHJ6 "1"
V_gene_freq_by_uniq_VDJ_Class_switched..IGHV1.18 "2"
V_gene_freq_by_uniq_VDJ_Class_switched..IGHV1.46 "2"
V_gene_freq_by_uniq_VDJ_Class_switched..IGHV1.58 "2"
V_gene_freq_by_uniq_VDJ_Class_switched..IGHV1.69 "2"
V_gene_freq_by_uniq_VDJ_expanded..IGHV1.18 "2"
V_gene_freq_by_uniq_VDJ_expanded..IGHV1.46 "2"
V_gene_freq_by_uniq_VDJ_expanded..IGHV1.58 "2"
V_gene_freq_by_uniq_VDJ_expanded..IGHV1.69 "2"
V_gene_freq_by_uniq_VDJ_unexpanded..IGHV1.18 "2"
V_gene_freq_by_uniq_VDJ_unexpanded..IGHV1.46 "2"
V_gene_freq_by_uniq_VDJ_unexpanded..IGHV1.58 "2"
V_gene_freq_by_uniq_VDJ_unexpanded..IGHV1.69 "2"
V_gene_freq_by_cluster_Class_switched..IGHV1.18 "2"
V_gene_freq_by_cluster_Class_switched..IGHV1.46 "2"
V_gene_freq_by_cluster_Class_switched..IGHV1.58 "2"
V_gene_freq_by_cluster_Class_switched..IGHV1.69 "2"
V_gene_freq_by_cluster_expanded..IGHV1.18 "2"
V_gene_freq_by_cluster_expanded..IGHV1.46 "2"
V_gene_freq_by_cluster_expanded..IGHV1.58 "2"
V_gene_freq_by_cluster_expanded..IGHV1.69 "2"
V_gene_freq_by_cluster_unexpanded..IGHV1.18 "2"
V_gene_freq_by_cluster_unexpanded..IGHV1.46 "2"
V_gene_freq_by_cluster_unexpanded..IGHV1.58 "2"
V_gene_freq_by_cluster_unexpanded..IGHV1.69 "2"
Percentage_unique_BCRs_per_isotype..IGHD.IGHM_unmutated "3"
Percentage_unmutated..Class_switched "3"
Percentage_unmutated..expanded "3"
Percentage_unmutated..unexpanded "3"
V_gene_freq_by_uniq_VDJ_Class_switched..IGHV1.24 "3"
V_gene_freq_by_uniq_VDJ_Class_switched..IGHV1.45 "3"
V_gene_freq_by_uniq_VDJ_Class_switched..IGHV7.4.1 "3"
V_gene_freq_by_uniq_VDJ_expanded..IGHV1.24 "3"
V_gene_freq_by_uniq_VDJ_expanded..IGHV1.45 "3"
V_gene_freq_by_uniq_VDJ_expanded..IGHV7.4.1 "3"
V_gene_freq_by_uniq_VDJ_unexpanded..IGHV1.24 "3"

V_gene_freq_by_uniq_VDJ_unexpanded..IGHV1.45 "3"
V_gene_freq_by_uniq_VDJ_unexpanded..IGHV1.NL1 "3"
V_gene_freq_by_uniq_VDJ_unexpanded..IGHV7.4.1 "3"
V_gene_freq_by_cluster_Class_switched..IGHV1.24 "3"
V_gene_freq_by_cluster_Class_switched..IGHV1.45 "3"
V_gene_freq_by_cluster_Class_switched..IGHV7.4.1 "3"
V_gene_freq_by_cluster_expanded..IGHV1.24 "3"
V_gene_freq_by_cluster_expanded..IGHV1.45 "3"
V_gene_freq_by_cluster_expanded..IGHV7.4.1 "3"
V_gene_freq_by_cluster_unexpanded..IGHV1.24 "3"
V_gene_freq_by_cluster_unexpanded..IGHV1.45 "3"
V_gene_freq_by_cluster_unexpanded..IGHV1.NL1 "3"
V_gene_freq_by_cluster_unexpanded..IGHV7.4.1 "3"
V_gene_freq_by_uniq_VDJ_Class_switched..IGHV4.31 "4"
V_gene_freq_by_uniq_VDJ_Class_switched..IGHV4.4 "4"
V_gene_freq_by_uniq_VDJ_Class_switched..IGHV4.61 "4"
V_gene_freq_by_uniq_VDJ_Class_switched..IGHV6.1 "4"
V_gene_freq_by_uniq_VDJ_expanded..IGHV4.31 "4"
V_gene_freq_by_uniq_VDJ_expanded..IGHV4.4 "4"
V_gene_freq_by_uniq_VDJ_expanded..IGHV4.61 "4"
V_gene_freq_by_uniq_VDJ_expanded..IGHV6.1 "4"
V_gene_freq_by_uniq_VDJ_unexpanded..IGHV4.31 "4"
V_gene_freq_by_uniq_VDJ_unexpanded..IGHV4.4 "4"
V_gene_freq_by_uniq_VDJ_unexpanded..IGHV4.61 "4"
V_gene_freq_by_uniq_VDJ_unexpanded..IGHV6.1 "4"
V_gene_freq_by_cluster_Class_switched..IGHV4.31 "4"
V_gene_freq_by_cluster_Class_switched..IGHV4.4 "4"
V_gene_freq_by_cluster_Class_switched..IGHV4.61 "4"
V_gene_freq_by_cluster_Class_switched..IGHV6.1 "4"
V_gene_freq_by_cluster_expanded..IGHV4.31 "4"
V_gene_freq_by_cluster_expanded..IGHV4.4 "4"
V_gene_freq_by_cluster_expanded..IGHV4.61 "4"
V_gene_freq_by_cluster_expanded..IGHV6.1 "4"
V_gene_freq_by_cluster_unexpanded..IGHV4.31 "4"
V_gene_freq_by_cluster_unexpanded..IGHV4.4 "4"
V_gene_freq_by_cluster_unexpanded..IGHV4.61 "4"
V_gene_freq_by_cluster_unexpanded..IGHV6.1 "4"
Mean_SHM_per_BCR..Class_switched "5"
Mean_SHM_per_BCR..expanded "5"
Mean_SHM_per_BCR..unexpanded "5"
Mean_mutations_per_BCR..IGHD_IGHM_mutated "5"
Mean_mutations_per_BCR..IGHG1 "5"
Mean_mutations_per_BCR..IGHG3 "5"
Mean_mutations_per_BCR..IGHM "5"
V_gene_freq_by_uniq_VDJ_Class_switched..IGHV3.11 "5"

V_gene_freq_by_uniq_VDJ_Class_switched..IGHV3.48 "5"
V_gene_freq_by_uniq_VDJ_expanded..IGHV3.11 "5"
V_gene_freq_by_uniq_VDJ_expanded..IGHV3.48 "5"
V_gene_freq_by_uniq_VDJ_unexpanded..IGHV3.11 "5"
V_gene_freq_by_uniq_VDJ_unexpanded..IGHV3.48 "5"
V_gene_freq_by_cluster_Class_switched..IGHV3.11 "5"
V_gene_freq_by_cluster_Class_switched..IGHV3.48 "5"
V_gene_freq_by_cluster_expanded..IGHV3.11 "5"
V_gene_freq_by_cluster_expanded..IGHV3.48 "5"
V_gene_freq_by_cluster_unexpanded..IGHV3.11 "5"
V_gene_freq_by_cluster_unexpanded..IGHV3.48 "5"
V_gene_freq_by_uniq_VDJ_Class_switched..IGHV2.26 "6"
V_gene_freq_by_uniq_VDJ_Class_switched..IGHV4.34 "6"
V_gene_freq_by_uniq_VDJ_Class_switched..IGHV5.51 "6"
V_gene_freq_by_uniq_VDJ_expanded..IGHV2.26 "6"
V_gene_freq_by_uniq_VDJ_expanded..IGHV4.34 "6"
V_gene_freq_by_uniq_VDJ_expanded..IGHV5.51 "6"
V_gene_freq_by_uniq_VDJ_unexpanded..IGHV2.26 "6"
V_gene_freq_by_uniq_VDJ_unexpanded..IGHV4.34 "6"
V_gene_freq_by_uniq_VDJ_unexpanded..IGHV5.51 "6"
V_gene_freq_by_cluster_Class_switched..IGHV2.26 "6"
V_gene_freq_by_cluster_Class_switched..IGHV4.34 "6"
V_gene_freq_by_cluster_Class_switched..IGHV5.51 "6"
V_gene_freq_by_cluster_expanded..IGHV2.26 "6"
V_gene_freq_by_cluster_expanded..IGHV4.34 "6"
V_gene_freq_by_cluster_expanded..IGHV5.51 "6"
V_gene_freq_by_cluster_unexpanded..IGHV2.26 "6"
V_gene_freq_by_cluster_unexpanded..IGHV4.34 "6"
V_gene_freq_by_cluster_unexpanded..IGHV5.51 "6"
V_gene_freq_by_uniq_VDJ_Class_switched..IGHV3.23 "7"
V_gene_freq_by_uniq_VDJ_Class_switched..IGHV3.7 "7"
V_gene_freq_by_uniq_VDJ_Class_switched..IGHV3.72 "7"
V_gene_freq_by_uniq_VDJ_expanded..IGHV3.23 "7"
V_gene_freq_by_uniq_VDJ_expanded..IGHV3.7 "7"
V_gene_freq_by_uniq_VDJ_expanded..IGHV3.72 "7"
V_gene_freq_by_uniq_VDJ_unexpanded..IGHV3.23 "7"
V_gene_freq_by_uniq_VDJ_unexpanded..IGHV3.7 "7"
V_gene_freq_by_uniq_VDJ_unexpanded..IGHV3.72 "7"
V_gene_freq_by_cluster_Class_switched..IGHV3.23 "7"
V_gene_freq_by_cluster_Class_switched..IGHV3.7 "7"
V_gene_freq_by_cluster_Class_switched..IGHV3.72 "7"
V_gene_freq_by_cluster_expanded..IGHV3.23 "7"
V_gene_freq_by_cluster_expanded..IGHV3.7 "7"
V_gene_freq_by_cluster_expanded..IGHV3.72 "7"
V_gene_freq_by_cluster_unexpanded..IGHV3.23 "7"

V_gene_freq_by_cluster_unexpanded..IGHV3.7 "7"
V_gene_freq_by_cluster_unexpanded..IGHV3.72 "7"
V_gene_freq_by_uniq_VDJ_Class_switched..IGHV3.13 "8"
V_gene_freq_by_uniq_VDJ_Class_switched..IGHV3.64 "8"
V_gene_freq_by_uniq_VDJ_Class_switched..IGHV3.66 "8"
V_gene_freq_by_uniq_VDJ_expanded..IGHV3.13 "8"
V_gene_freq_by_uniq_VDJ_expanded..IGHV3.64 "8"
V_gene_freq_by_uniq_VDJ_expanded..IGHV3.66 "8"
V_gene_freq_by_uniq_VDJ_unexpanded..IGHV3.13 "8"
V_gene_freq_by_uniq_VDJ_unexpanded..IGHV3.64 "8"
V_gene_freq_by_uniq_VDJ_unexpanded..IGHV3.66 "8"
V_gene_freq_by_cluster_Class_switched..IGHV3.13 "8"
V_gene_freq_by_cluster_Class_switched..IGHV3.64 "8"
V_gene_freq_by_cluster_Class_switched..IGHV3.66 "8"
V_gene_freq_by_cluster_expanded..IGHV3.13 "8"
V_gene_freq_by_cluster_expanded..IGHV3.64 "8"
V_gene_freq_by_cluster_expanded..IGHV3.66 "8"
V_gene_freq_by_cluster_unexpanded..IGHV3.13 "8"
V_gene_freq_by_cluster_unexpanded..IGHV3.64 "8"
V_gene_freq_by_cluster_unexpanded..IGHV3.66 "8"
J_gene_freq_by_uniq_VDJ_IGHD.IGHM_unmutated..IGHJ2 "9"
J_gene_freq_by_uniq_VDJ_IGHD.IGHM_unmutated..IGHJ3 "9"
J_gene_freq_by_uniq_VDJ_IGHD.IGHM_unmutated..IGHJ4 "9"
J_gene_freq_by_uniq_VDJ_IGHD.IGHM_unmutated..IGHJ5 "9"
J_gene_freq_by_uniq_VDJ_IGHD.IGHM_unmutated..IGHJ6 "9"
J_gene_freq_by_uniq_VDJ_IGHE..IGHJ3 "9"
J_gene_freq_by_uniq_VDJ_IGHG4..IGHJ3 "9"
V_gene_freq_by_uniq_VDJ_Class_switched..IGHV1.69.2 "9"
V_gene_freq_by_uniq_VDJ_Class_switched..IGHV4.55 "9"
V_gene_freq_by_uniq_VDJ_unexpanded..IGHV1.68 "9"
V_gene_freq_by_uniq_VDJ_unexpanded..IGHV1.69.2 "9"
V_gene_freq_by_uniq_VDJ_unexpanded..IGHV4.55 "9"
V_gene_freq_by_cluster_Class_switched..IGHV1.69.2 "9"
V_gene_freq_by_cluster_Class_switched..IGHV4.55 "9"
V_gene_freq_by_cluster_expanded..IGHV1.69.2 "9"
V_gene_freq_by_cluster_unexpanded..IGHV1.68 "9"
V_gene_freq_by_cluster_unexpanded..IGHV1.69.2 "9"
V_gene_freq_by_cluster_unexpanded..IGHV4.55 "9"
V_gene_freq_by_uniq_VDJ_Class_switched..IGHV3.43 "10"
V_gene_freq_by_uniq_VDJ_Class_switched..IGHV3.53 "10"
V_gene_freq_by_uniq_VDJ_Class_switched..IGHV3.74 "10"
V_gene_freq_by_uniq_VDJ_expanded..IGHV3.43 "10"
V_gene_freq_by_uniq_VDJ_expanded..IGHV3.74 "10"
V_gene_freq_by_uniq_VDJ_unexpanded..IGHV3.43 "10"
V_gene_freq_by_uniq_VDJ_unexpanded..IGHV3.53 "10"

V_gene_freq_by_uniq_VDJ_unexpanded..IGHV3.74 "10"
V_gene_freq_by_cluster_Class_switched..IGHV3.43 "10"
V_gene_freq_by_cluster_Class_switched..IGHV3.53 "10"
V_gene_freq_by_cluster_Class_switched..IGHV3.74 "10"
V_gene_freq_by_cluster_expanded..IGHV3.43 "10"
V_gene_freq_by_cluster_expanded..IGHV3.53 "10"
V_gene_freq_by_cluster_expanded..IGHV3.74 "10"
V_gene_freq_by_cluster_unexpanded..IGHV3.43 "10"
V_gene_freq_by_cluster_unexpanded..IGHV3.53 "10"
V_gene_freq_by_cluster_unexpanded..IGHV3.74 "10"
J_gene_freq_by_uniq_VDJ_Class_switched..IGHJ1 "11"
J_gene_freq_by_uniq_VDJ_IGHA1..IGHJ1 "11"
J_gene_freq_by_uniq_VDJ_IGHA2..IGHJ1 "11"
J_gene_freq_by_uniq_VDJ_IGHD..IGHJ1 "11"
J_gene_freq_by_uniq_VDJ_IGHG1..IGHJ1 "11"
J_gene_freq_by_uniq_VDJ_IGHG2..IGHJ1 "11"
J_gene_freq_by_uniq_VDJ_IGHG3..IGHJ1 "11"
J_gene_freq_by_uniq_VDJ_IGHG4..IGHJ1 "11"
J_gene_freq_by_uniq_VDJ_IGHM..IGHJ1 "11"
J_gene_freq_by_uniq_VDJ_expanded..IGHJ1 "11"
V_gene_freq_by_uniq_VDJ_Class_switched..IGHV4.38.2 "11"
V_gene_freq_by_uniq_VDJ_expanded..IGHV4.38.2 "11"
V_gene_freq_by_uniq_VDJ_unexpanded..IGHV4.38.2 "11"
V_gene_freq_by_cluster_Class_switched..IGHV4.38.2 "11"
V_gene_freq_by_cluster_expanded..IGHV4.38.2 "11"
V_gene_freq_by_cluster_unexpanded..IGHV4.38.2 "11"
J_gene_freq_by_uniq_VDJ_Class_switched..IGHJ3 "12"
J_gene_freq_by_uniq_VDJ_IGHA1..IGHJ3 "12"
J_gene_freq_by_uniq_VDJ_IGHA2..IGHJ3 "12"
J_gene_freq_by_uniq_VDJ_IGHD..IGHJ3 "12"
J_gene_freq_by_uniq_VDJ_IGHG1..IGHJ3 "12"
J_gene_freq_by_uniq_VDJ_IGHG2..IGHJ3 "12"
J_gene_freq_by_uniq_VDJ_IGHG3..IGHJ3 "12"
J_gene_freq_by_uniq_VDJ_IGHM..IGHJ3 "12"
J_gene_freq_by_uniq_VDJ_expanded..IGHJ3 "12"
V_gene_freq_by_uniq_VDJ_Class_switched..IGHV3.73 "12"
V_gene_freq_by_uniq_VDJ_expanded..IGHV3.73 "12"
V_gene_freq_by_uniq_VDJ_unexpanded..IGHV3.73 "12"
V_gene_freq_by_cluster_Class_switched..IGHV3.73 "12"
V_gene_freq_by_cluster_expanded..IGHV3.73 "12"
V_gene_freq_by_cluster_unexpanded..IGHV3.73 "12"
V_gene_freq_by_uniq_VDJ_Class_switched..IGHV3.15 "13"
V_gene_freq_by_uniq_VDJ_Class_switched..IGHV3.21 "13"
V_gene_freq_by_uniq_VDJ_expanded..IGHV3.15 "13"
V_gene_freq_by_uniq_VDJ_expanded..IGHV3.21 "13"

V_gene_freq_by_uniq_VDJ_unexpanded..IGHV3.15 "13"
V_gene_freq_by_uniq_VDJ_unexpanded..IGHV3.21 "13"
V_gene_freq_by_uniq_VDJ_unexpanded..IGHV3.35 "13"
V_gene_freq_by_cluster_Class_switched..IGHV3.15 "13"
V_gene_freq_by_cluster_Class_switched..IGHV3.21 "13"
V_gene_freq_by_cluster_expanded..IGHV3.15 "13"
V_gene_freq_by_cluster_expanded..IGHV3.21 "13"
V_gene_freq_by_cluster_expanded..IGHV3.69.1 "13"
V_gene_freq_by_cluster_unexpanded..IGHV3.15 "13"
V_gene_freq_by_cluster_unexpanded..IGHV3.21 "13"
V_gene_freq_by_cluster_unexpanded..IGHV3.35 "13"
V_gene_freq_by_uniq_VDJ_Class_switched..IGHV3.30 "14"
V_gene_freq_by_uniq_VDJ_Class_switched..IGHV3.30.3 "14"
V_gene_freq_by_uniq_VDJ_expanded..IGHV3.30 "14"
V_gene_freq_by_uniq_VDJ_expanded..IGHV3.30.3 "14"
V_gene_freq_by_uniq_VDJ_expanded..IGHV3.NL1 "14"
V_gene_freq_by_uniq_VDJ_unexpanded..IGHV3.30 "14"
V_gene_freq_by_uniq_VDJ_unexpanded..IGHV3.30.3 "14"
V_gene_freq_by_cluster_Class_switched..IGHV3.30 "14"
V_gene_freq_by_cluster_Class_switched..IGHV3.30.3 "14"
V_gene_freq_by_cluster_expanded..IGHV3.30 "14"
V_gene_freq_by_cluster_expanded..IGHV3.30.3 "14"
V_gene_freq_by_cluster_expanded..IGHV3.NL1 "14"
V_gene_freq_by_cluster_unexpanded..IGHV3.30 "14"
V_gene_freq_by_cluster_unexpanded..IGHV3.30.3 "14"
Percentage_unique_BCRs_per_isotype..IGHD.IGHM_mutated "15"
J_gene_freq_by_uniq_VDJ_Class_switched..IGHJ4 "15"
J_gene_freq_by_uniq_VDJ_IGHA1..IGHJ4 "15"
J_gene_freq_by_uniq_VDJ_IGHA2..IGHJ4 "15"
J_gene_freq_by_uniq_VDJ_IGHD..IGHJ4 "15"
J_gene_freq_by_uniq_VDJ_IGHD.IGHM_mutated..IGHJ5 "15"
J_gene_freq_by_uniq_VDJ_IGHD.IGHM_mutated..IGHJ6 "15"
J_gene_freq_by_uniq_VDJ_IGHG1..IGHJ4 "15"
J_gene_freq_by_uniq_VDJ_IGHG2..IGHJ4 "15"
J_gene_freq_by_uniq_VDJ_IGHG3..IGHJ4 "15"
J_gene_freq_by_uniq_VDJ_IGHM..IGHJ4 "15"
J_gene_freq_by_uniq_VDJ_expanded..IGHJ4 "15"
J_gene_freq_by_uniq_VDJ_unexpanded..IGHJ5 "15"
J_gene_freq_by_uniq_VDJ_unexpanded..IGHJ6 "15"
Vertex_Reyni..all "16"
Cluster_Reyni..all "16"
J_gene_freq_by_uniq_VDJ_Class_switched..IGHJ2 "16"
J_gene_freq_by_uniq_VDJ_IGHA1..IGHJ2 "16"
J_gene_freq_by_uniq_VDJ_IGHA2..IGHJ2 "16"
J_gene_freq_by_uniq_VDJ_IGHD..IGHJ2 "16"

J_gene_freq_by_uniq_VDJ_IGHG1..IGHJ2 "16"
J_gene_freq_by_uniq_VDJ_IGHG2..IGHJ2 "16"
J_gene_freq_by_uniq_VDJ_IGHG3..IGHJ2 "16"
J_gene_freq_by_uniq_VDJ_IGHM..IGHJ2 "16"
J_gene_freq_by_uniq_VDJ_expanded..IGHJ2 "16"
V_gene_freq_by_uniq_VDJ_expanded..IGHV3.53 "16"
V_gene_freq_by_uniq_VDJ_expanded..IGHV3.69.1 "16"
V_gene_freq_by_uniq_VDJ_Class_switched..IGHV2.5 "17"
V_gene_freq_by_uniq_VDJ_Class_switched..IGHV2.70 "17"
V_gene_freq_by_uniq_VDJ_expanded..IGHV2.5 "17"
V_gene_freq_by_uniq_VDJ_expanded..IGHV2.70 "17"
V_gene_freq_by_uniq_VDJ_unexpanded..IGHV2.5 "17"
V_gene_freq_by_uniq_VDJ_unexpanded..IGHV2.70 "17"
V_gene_freq_by_cluster_Class_switched..IGHV2.5 "17"
V_gene_freq_by_cluster_Class_switched..IGHV2.70 "17"
V_gene_freq_by_cluster_expanded..IGHV2.5 "17"
V_gene_freq_by_cluster_expanded..IGHV2.70 "17"
V_gene_freq_by_cluster_unexpanded..IGHV2.5 "17"
V_gene_freq_by_cluster_unexpanded..IGHV2.70 "17"
V_gene_freq_by_uniq_VDJ_Class_switched..IGHV1.2 "18"
V_gene_freq_by_uniq_VDJ_Class_switched..IGHV3.20 "18"
V_gene_freq_by_uniq_VDJ_expanded..IGHV1.2 "18"
V_gene_freq_by_uniq_VDJ_expanded..IGHV3.20 "18"
V_gene_freq_by_uniq_VDJ_unexpanded..IGHV1.2 "18"
V_gene_freq_by_uniq_VDJ_unexpanded..IGHV3.20 "18"
V_gene_freq_by_cluster_Class_switched..IGHV1.2 "18"
V_gene_freq_by_cluster_Class_switched..IGHV3.20 "18"
V_gene_freq_by_cluster_expanded..IGHV1.2 "18"
V_gene_freq_by_cluster_expanded..IGHV3.20 "18"
V_gene_freq_by_cluster_unexpanded..IGHV1.2 "18"
V_gene_freq_by_cluster_unexpanded..IGHV3.20 "18"
Mean_mutations_per_BCR..IGHA1 "19"
Mean_mutations_per_BCR..IGHA2 "19"
Mean_mutations_per_BCR..IGHD "19"
Mean_mutations_per_BCR..IGHG2 "19"
J_gene_freq_by_uniq_VDJ_IGHD.IGHM_mutated..IGHJ1 "19"
J_gene_freq_by_uniq_VDJ_IGHD.IGHM_mutated..IGHJ2 "19"
J_gene_freq_by_uniq_VDJ_IGHD.IGHM_mutated..IGHJ3 "19"
J_gene_freq_by_uniq_VDJ_IGHD.IGHM_mutated..IGHJ4 "19"
J_gene_freq_by_uniq_VDJ_unexpanded..IGHJ1 "19"
J_gene_freq_by_uniq_VDJ_unexpanded..IGHJ2 "19"
J_gene_freq_by_uniq_VDJ_unexpanded..IGHJ3 "19"
J_gene_freq_by_uniq_VDJ_unexpanded..IGHJ4 "19"
V_gene_freq_by_uniq_VDJ_Class_switched..IGHV4.28 "20"
V_gene_freq_by_uniq_VDJ_Class_switched..IGHV4.39 "20"

V_gene_freq_by_uniq_VDJ_expanded..IGHV4.28 "20"
V_gene_freq_by_uniq_VDJ_expanded..IGHV4.39 "20"
V_gene_freq_by_uniq_VDJ_unexpanded..IGHV4.28 "20"
V_gene_freq_by_uniq_VDJ_unexpanded..IGHV4.39 "20"
V_gene_freq_by_cluster_Class_switched..IGHV4.28 "20"
V_gene_freq_by_cluster_Class_switched..IGHV4.39 "20"
V_gene_freq_by_cluster_expanded..IGHV4.28 "20"
V_gene_freq_by_cluster_expanded..IGHV4.39 "20"
V_gene_freq_by_cluster_unexpanded..IGHV4.28 "20"
V_gene_freq_by_cluster_unexpanded..IGHV4.39 "20"
Percentage_max_cluster_size..IGHG4 "21"
Percentage_max_vertex_size..IGHG4 "21"
Mean_mutations_per_BCR..IGHG4 "21"
J_gene_freq_by_uniq_VDJ_IGHG4..IGHJ6 "21"
V_gene_freq_by_uniq_VDJ_Class_switched..IGHV1.8 "21"
V_gene_freq_by_uniq_VDJ_expanded..IGHV1.8 "21"
V_gene_freq_by_uniq_VDJ_unexpanded..IGHV1.8 "21"
V_gene_freq_by_uniq_VDJ_unexpanded..IGHV3.52 "21"
V_gene_freq_by_cluster_Class_switched..IGHV1.8 "21"
V_gene_freq_by_cluster_expanded..IGHV1.8 "21"
V_gene_freq_by_cluster_unexpanded..IGHV1.8 "21"
V_gene_freq_by_cluster_unexpanded..IGHV3.52 "21"
V_gene_freq_by_uniq_VDJ_Class_switched..IGHV4.30.2 "22"
V_gene_freq_by_uniq_VDJ_Class_switched..IGHV4.30.4 "22"
V_gene_freq_by_uniq_VDJ_expanded..IGHV4.30.2 "22"
V_gene_freq_by_uniq_VDJ_expanded..IGHV4.30.4 "22"
V_gene_freq_by_uniq_VDJ_unexpanded..IGHV4.30.2 "22"
V_gene_freq_by_uniq_VDJ_unexpanded..IGHV4.30.4 "22"
V_gene_freq_by_cluster_Class_switched..IGHV4.30.2 "22"
V_gene_freq_by_cluster_Class_switched..IGHV4.30.4 "22"
V_gene_freq_by_cluster_expanded..IGHV4.30.2 "22"
V_gene_freq_by_cluster_expanded..IGHV4.30.4 "22"
V_gene_freq_by_cluster_unexpanded..IGHV4.30.2 "22"
V_gene_freq_by_cluster_unexpanded..IGHV4.30.4 "22"
V_gene_freq_by_uniq_VDJ_Class_switched..IGHV1.3 "23"
V_gene_freq_by_uniq_VDJ_Class_switched..IGHV3.49 "23"
V_gene_freq_by_uniq_VDJ_expanded..IGHV1.3 "23"
V_gene_freq_by_uniq_VDJ_expanded..IGHV3.49 "23"
V_gene_freq_by_uniq_VDJ_unexpanded..IGHV1.3 "23"
V_gene_freq_by_uniq_VDJ_unexpanded..IGHV3.49 "23"
V_gene_freq_by_cluster_Class_switched..IGHV1.3 "23"
V_gene_freq_by_cluster_Class_switched..IGHV3.49 "23"
V_gene_freq_by_cluster_expanded..IGHV1.3 "23"
V_gene_freq_by_cluster_expanded..IGHV3.49 "23"
V_gene_freq_by_cluster_unexpanded..IGHV1.3 "23"

V_gene_freq_by_cluster_unexpanded..IGHV3.49 "23"
V_gene_freq_by_uniq_VDJ_Class_switched..IGHV3.64D "24"
V_gene_freq_by_uniq_VDJ_Class_switched..IGHV5.10.1 "24"
V_gene_freq_by_uniq_VDJ_expanded..IGHV3.64D "24"
V_gene_freq_by_uniq_VDJ_expanded..IGHV5.10.1 "24"
V_gene_freq_by_uniq_VDJ_unexpanded..IGHV3.64D "24"
V_gene_freq_by_uniq_VDJ_unexpanded..IGHV5.10.1 "24"
V_gene_freq_by_cluster_Class_switched..IGHV3.64D "24"
V_gene_freq_by_cluster_Class_switched..IGHV5.10.1 "24"
V_gene_freq_by_cluster_expanded..IGHV3.64D "24"
V_gene_freq_by_cluster_expanded..IGHV5.10.1 "24"
V_gene_freq_by_cluster_unexpanded..IGHV3.64D "24"
V_gene_freq_by_cluster_unexpanded..IGHV5.10.1 "24"
J_gene_freq_by_uniq_VDJ_Class_switched..IGHJ5 "25"
J_gene_freq_by_uniq_VDJ_IGHA1..IGHJ5 "25"
J_gene_freq_by_uniq_VDJ_IGHA2..IGHJ5 "25"
J_gene_freq_by_uniq_VDJ_IGHD..IGHJ5 "25"
J_gene_freq_by_uniq_VDJ_IGHE..IGHJ5 "25"
J_gene_freq_by_uniq_VDJ_IGHG1..IGHJ5 "25"
J_gene_freq_by_uniq_VDJ_IGHG2..IGHJ5 "25"
J_gene_freq_by_uniq_VDJ_IGHG3..IGHJ5 "25"
J_gene_freq_by_uniq_VDJ_IGHG4..IGHJ5 "25"
J_gene_freq_by_uniq_VDJ_IGHM..IGHJ5 "25"
J_gene_freq_by_uniq_VDJ_expanded..IGHJ5 "25"
V_gene_freq_by_uniq_VDJ_Class_switched..IGHV3.71 "26"
V_gene_freq_by_uniq_VDJ_Class_switched..IGHV3.9 "26"
V_gene_freq_by_uniq_VDJ_expanded..IGHV3.9 "26"
V_gene_freq_by_uniq_VDJ_unexpanded..IGHV3.71 "26"
V_gene_freq_by_uniq_VDJ_unexpanded..IGHV3.9 "26"
V_gene_freq_by_cluster_Class_switched..IGHV3.71 "26"
V_gene_freq_by_cluster_Class_switched..IGHV3.9 "26"
V_gene_freq_by_cluster_expanded..IGHV3.9 "26"
V_gene_freq_by_cluster_unexpanded..IGHV3.71 "26"
V_gene_freq_by_cluster_unexpanded..IGHV3.9 "26"
V_gene_freq_by_uniq_VDJ_Class_switched..IGHV3.43D "27"
V_gene_freq_by_uniq_VDJ_Class_switched..IGHV3.69.1 "27"
V_gene_freq_by_uniq_VDJ_expanded..IGHV3.43D "27"
V_gene_freq_by_uniq_VDJ_unexpanded..IGHV3.43D "27"
V_gene_freq_by_uniq_VDJ_unexpanded..IGHV3.69.1 "27"
V_gene_freq_by_cluster_Class_switched..IGHV3.43D "27"
V_gene_freq_by_cluster_Class_switched..IGHV3.69.1 "27"
V_gene_freq_by_cluster_expanded..IGHV3.43D "27"
V_gene_freq_by_cluster_unexpanded..IGHV3.43D "27"
V_gene_freq_by_cluster_unexpanded..IGHV3.69.1 "27"
Percentage_max_cluster_size..IGHG2 "28"

Percentage_max_vertex_size..IGHG2 "28"
V_gene_replacement_clonal_expansion..mean_clone_size_norm "28"
V_gene_replacement_clonal_expansion..mean_clone_size_secondary "28"
V_gene_freq_by_uniq_VDJ_Class_switched..IGHV4.59 "28"
V_gene_freq_by_uniq_VDJ_expanded..IGHV4.59 "28"
V_gene_freq_by_uniq_VDJ_unexpanded..IGHV4.59 "28"
V_gene_freq_by_cluster_Class_switched..IGHV4.59 "28"
V_gene_freq_by_cluster_expanded..IGHV4.59 "28"
V_gene_freq_by_cluster_unexpanded..IGHV4.59 "28"
V_gene_freq_by_uniq_VDJ_Class_switched..IGHV3.33 "29"
V_gene_freq_by_uniq_VDJ_Class_switched..IGHV3.NL1 "29"
V_gene_freq_by_uniq_VDJ_expanded..IGHV3.33 "29"
V_gene_freq_by_uniq_VDJ_unexpanded..IGHV3.33 "29"
V_gene_freq_by_uniq_VDJ_unexpanded..IGHV3.NL1 "29"
V_gene_freq_by_cluster_Class_switched..IGHV3.33 "29"
V_gene_freq_by_cluster_Class_switched..IGHV3.NL1 "29"
V_gene_freq_by_cluster_expanded..IGHV3.33 "29"
V_gene_freq_by_cluster_unexpanded..IGHV3.33 "29"
V_gene_freq_by_cluster_unexpanded..IGHV3.NL1 "29"

Table 3 – Module and features associated with flow-sorted data

"Feature" "Cluster"
"Percentage_unique_BCRs_per_isotype_group..IGHE.1" "1"
"Percentage_unique_BCRs_in_switched..IGHE.1" "1"
"Relative_class_switching_absolute..IGHA1.01.IGHE.02.1" "1"
"D50..IGHE.02" "1"
"Relative_class_switching_normalised..IGHA1.IGHE.1" "1"
"Relative_class_switching_normalised..IGHD.M.IGHE.1" "1"
"V_gene_freq_by_uniq_VDJ_IGHE..IGHV2.70D" "1"
"V_gene_freq_by_uniq_VDJ_IGHE..IGHV3.13" "1"
"V_gene_freq_by_uniq_VDJ_IGHE..IGHV4.28" "1"
"V_gene_freq_by_uniq_VDJ_IGHE..IGHV4.38.2" "1"
"V_gene_freq_by_cluster_IGHE..IGHV2.70D" "1"
"V_gene_freq_by_cluster_IGHE..IGHV4.28" "1"
"V_gene_freq_by_cluster_IGHE..IGHV4.38.2" "1"
"V_gene_freq_by_cluster_IGHE..IGHV6.1" "1"
"Mean_cluster_size_IGHE..IGHV2.70D" "1"
"Mean_cluster_size_IGHE..IGHV3.13" "1"
"Mean_cluster_size_IGHE..IGHV3.15" "1"
"Mean_cluster_size_IGHE..IGHV3.43" "1"
"Mean_cluster_size_IGHE..IGHV3.69.1" "1"
"Mean_cluster_size_IGHE..IGHV3.73" "1"
"Mean_cluster_size_IGHE..IGHV4.30.2" "1"
"Mean_cluster_size_IGHE..IGHV4.30.4" "1"
"Mean_cluster_size_IGHE..IGHV4.31" "1"

"Mean_cluster_size_IGHE..IGHV4.38.2" "1"
"Vertex_Reyni..IGHG3.1" "2"
"Cluster_Reyni..IGHG3.1" "2"
"Percentage_unique_BCRs_per_isotype_group..IGHG3.1" "2"
"Mean_CDR3_lengths..IGHG3.1" "2"
"FWR3_mm..IGHG3.1" "2"
"mean_CDR_FWR_ratio..IGHG3.1" "2"
"mean_CDR_mm_per_BCR..IGHG3.1" "2"
"mean_FWR_mm_per_BCR..IGHG3.1" "2"
"Mean_mutations_per_BCR..IGHG3.1" "2"
"D10..IGHG3.18.1" "2"
"D50..IGHG3.18" "2"
"mean_CDR3_charge..IGHG3.1" "2"
"J_gene_freq_by_uniq_VDJ_IGHG3..IGHJ4.1" "2"
"V_gene_freq_by_cluster_IGHG3..IGHV3.11.1" "2"
"V_gene_freq_by_cluster_IGHG3..IGHV3.21.1" "2"
"V_gene_freq_by_cluster_IGHG3..IGHV3.23.1" "2"
"V_gene_freq_by_cluster_IGHG3..IGHV3.30.1" "2"
"V_gene_freq_by_cluster_IGHG3..IGHV3.48.1" "2"
"V_gene_freq_by_cluster_IGHG3..IGHV3.53.1" "2"
"V_gene_freq_by_cluster_IGHG3..IGHV3.7.1" "2"
"V_gene_freq_by_cluster_IGHG3..IGHV3.74.1" "2"
"V_gene_freq_by_uniq_VDJ_Class_switched..IGHV3.64" "3"
"V_gene_freq_by_uniq_VDJ_expanded..IGHV3.64" "3"
"V_gene_freq_by_uniq_VDJ_IGHA1..IGHV3.64" "3"
"V_gene_freq_by_uniq_VDJ_IGHD.IGHM_mutated..IGHV3.64" "3"
"V_gene_freq_by_uniq_VDJ_IGHG2..IGHV3.64" "3"
"V_gene_freq_by_uniq_VDJ_IGHG3..IGHV3.64" "3"
"V_gene_freq_by_uniq_VDJ_IGHM..IGHV3.64" "3"
"V_gene_freq_by_uniq_VDJ_unexpanded..IGHV3.64" "3"
"V_gene_freq_by_cluster_Class_switched..IGHV3.64" "3"
"V_gene_freq_by_cluster_expanded..IGHV3.64" "3"
"V_gene_freq_by_cluster_IGHA1..IGHV3.64" "3"
"V_gene_freq_by_cluster_IGHD..IGHV3.64" "3"
"V_gene_freq_by_cluster_IGHD.IGHM_mutated..IGHV3.64" "3"
"V_gene_freq_by_cluster_IGHG2..IGHV3.64" "3"
"V_gene_freq_by_cluster_IGHG3..IGHV3.64" "3"
"V_gene_freq_by_cluster_IGHM..IGHV3.64" "3"
"V_gene_freq_by_cluster_unexpanded..IGHV3.64" "3"
"V_gene_freq_by_uniq_VDJ_IGHG3..IGHV7.4.1" "3"
"V_gene_freq_by_cluster_IGHG3..IGHV7.4.1" "3"
"Mean_cluster_size_IGHG3..IGHV7.4.1" "3"
"V_gene_freq_by_uniq_VDJ_Class_switched..IGHV2.70D" "4"
"V_gene_freq_by_uniq_VDJ_expanded..IGHV2.70D" "4"
"V_gene_freq_by_uniq_VDJ_IGHA1..IGHV2.70D" "4"
"V_gene_freq_by_uniq_VDJ_IGHD..IGHV2.70D" "4"
"V_gene_freq_by_uniq_VDJ_IGHD.IGHM_mutated..IGHV2.70D" "4"
"V_gene_freq_by_uniq_VDJ_IGHG2..IGHV2.70D" "4"
"V_gene_freq_by_uniq_VDJ_IGHG3..IGHV2.70D" "4"

"V_gene_freq_by_uniq_VDJ_IGHM..IGHV2.70D" "4"
"V_gene_freq_by_uniq_VDJ_IGHM..IGHV3.35" "4"
"V_gene_freq_by_uniq_VDJ_unexpanded..IGHV2.70D" "4"
"V_gene_freq_by_cluster_Class_switched..IGHV2.70D" "4"
"V_gene_freq_by_cluster_expanded..IGHV2.70D" "4"
"V_gene_freq_by_cluster_IGHA1..IGHV2.70D" "4"
"V_gene_freq_by_cluster_IGHD..IGHV2.70D" "4"
"V_gene_freq_by_cluster_IGHD.IGHM_mutated..IGHV2.70D" "4"
"V_gene_freq_by_cluster_IGHG2..IGHV2.70D" "4"
"V_gene_freq_by_cluster_IGHG3..IGHV2.70D" "4"
"V_gene_freq_by_cluster_IGHM..IGHV2.70D" "4"
"V_gene_freq_by_cluster_IGHM..IGHV3.35" "4"
"V_gene_freq_by_cluster_unexpanded..IGHV2.70D" "4"
"V_gene_freq_by_uniq_VDJ_Class_switched..IGHV3.66" "5"
"V_gene_freq_by_uniq_VDJ_expanded..IGHV3.66" "5"
"V_gene_freq_by_uniq_VDJ_IGHA1..IGHV3.66" "5"
"V_gene_freq_by_uniq_VDJ_IGHD..IGHV3.66" "5"
"V_gene_freq_by_uniq_VDJ_IGHD.IGHM_mutated..IGHV3.66" "5"
"V_gene_freq_by_uniq_VDJ_IGHG2..IGHV3.66" "5"
"V_gene_freq_by_uniq_VDJ_IGHG3..IGHV3.66" "5"
"V_gene_freq_by_uniq_VDJ_IGHM..IGHV3.66" "5"
"V_gene_freq_by_uniq_VDJ_unexpanded..IGHV3.66" "5"
"V_gene_freq_by_cluster_Class_switched..IGHV3.66" "5"
"V_gene_freq_by_cluster_expanded..IGHV3.66" "5"
"V_gene_freq_by_cluster_IGHA1..IGHV3.66" "5"
"V_gene_freq_by_cluster_IGHD..IGHV3.66" "5"
"V_gene_freq_by_cluster_IGHD.IGHM_mutated..IGHV3.66" "5"
"V_gene_freq_by_cluster_IGHG2..IGHV3.66" "5"
"V_gene_freq_by_cluster_IGHG3..IGHV3.66" "5"
"V_gene_freq_by_cluster_IGHM..IGHV3.66" "5"
"V_gene_freq_by_cluster_unexpanded..IGHV3.66" "5"
"V4_34_AVY_unmut..IGHG3" "6"
"V_gene_freq_by_uniq_VDJ_expanded..IGHV3.69.1" "6"
"V_gene_freq_by_uniq_VDJ_IGHA1..IGHV3.69.1" "6"
"V_gene_freq_by_uniq_VDJ_IGHD.IGHM_mutated..IGHV3.69.1" "6"
"V_gene_freq_by_uniq_VDJ_IGHD.IGHM_mutated..IGHV3.NL1" "6"
"V_gene_freq_by_uniq_VDJ_IGHG2..IGHV3.69.1" "6"
"V_gene_freq_by_uniq_VDJ_IGHG3..IGHV3.69.1" "6"
"V_gene_freq_by_uniq_VDJ_IGHM..IGHV3.69.1" "6"
"V_gene_freq_by_uniq_VDJ_unexpanded..IGHV3.69.1" "6"
"V_gene_freq_by_cluster_expanded..IGHV3.69.1" "6"
"V_gene_freq_by_cluster_IGHA1..IGHV3.69.1" "6"
"V_gene_freq_by_cluster_IGHD.IGHM_mutated..IGHV3.69.1" "6"
"V_gene_freq_by_cluster_IGHD.IGHM_mutated..IGHV3.NL1" "6"
"V_gene_freq_by_cluster_IGHG2..IGHV3.69.1" "6"
"V_gene_freq_by_cluster_IGHG3..IGHV3.69.1" "6"
"V_gene_freq_by_cluster_IGHM..IGHV3.69.1" "6"
"V_gene_freq_by_cluster_unexpanded..IGHV3.69.1" "6"
"V_gene_freq_by_uniq_VDJ_IGHG3..IGHV3.47" "6"

"V_gene_freq_by_uniq_VDJ_Class_switched..IGHV3.53" "7"
"V_gene_freq_by_uniq_VDJ_expanded..IGHV3.53" "7"
"V_gene_freq_by_uniq_VDJ_IGHA1..IGHV3.53" "7"
"V_gene_freq_by_uniq_VDJ_IGHD..IGHV3.53" "7"
"V_gene_freq_by_uniq_VDJ_IGHD.IGHM_mutated..IGHV3.53" "7"
"V_gene_freq_by_uniq_VDJ_IGHG2..IGHV3.53" "7"
"V_gene_freq_by_uniq_VDJ_IGHG3..IGHV3.53" "7"
"V_gene_freq_by_uniq_VDJ_IGHM..IGHV3.53" "7"
"V_gene_freq_by_uniq_VDJ_unexpanded..IGHV3.53" "7"
"V_gene_freq_by_cluster_Class_switched..IGHV3.53" "7"
"V_gene_freq_by_cluster_expanded..IGHV3.53" "7"
"V_gene_freq_by_cluster_IGHA1..IGHV3.53" "7"
"V_gene_freq_by_cluster_IGHD..IGHV3.53" "7"
"V_gene_freq_by_cluster_IGHD.IGHM_mutated..IGHV3.53" "7"
"V_gene_freq_by_cluster_IGHG2..IGHV3.53" "7"
"V_gene_freq_by_cluster_IGHG3..IGHV3.53" "7"
"V_gene_freq_by_cluster_IGHM..IGHV3.53" "7"
"V_gene_freq_by_cluster_unexpanded..IGHV3.53" "7"
"V_gene_freq_by_uniq_VDJ_Class_switched..IGHV4.61" "8"
"V_gene_freq_by_uniq_VDJ_expanded..IGHV4.61" "8"
"V_gene_freq_by_uniq_VDJ_IGHA1..IGHV4.61" "8"
"V_gene_freq_by_uniq_VDJ_IGHG2..IGHV4.61" "8"
"V_gene_freq_by_uniq_VDJ_IGHG3..IGHV4.61" "8"
"V_gene_freq_by_uniq_VDJ_IGHM..IGHV4.61" "8"
"V_gene_freq_by_uniq_VDJ_unexpanded..IGHV4.61" "8"
"V_gene_freq_by_cluster_Class_switched..IGHV4.61" "8"
"V_gene_freq_by_cluster_expanded..IGHV4.61" "8"
"V_gene_freq_by_cluster_IGHA1..IGHV4.61" "8"
"V_gene_freq_by_cluster_IGHG2..IGHV4.61" "8"
"V_gene_freq_by_cluster_IGHG3..IGHV4.39" "8"
"V_gene_freq_by_cluster_IGHG3..IGHV4.59" "8"
"V_gene_freq_by_cluster_IGHG3..IGHV4.61" "8"
"V_gene_freq_by_cluster_IGHM..IGHV4.61" "8"
"V_gene_freq_by_cluster_unexpanded..IGHV4.61" "8"
"V_gene_freq_by_uniq_VDJ_IGHG3..IGHV3.9.1" "8"
"Mean_cluster_size_IGHG3..IGHV3.9" "8"
"V4_34_AVY_unmut..IGHD" "9"
"V4_34_AVY_unmut..IGHM" "9"
"V4_34_NHS_unmut..IGHD" "9"
"V4_34_NHS_unmut..IGHM" "9"
"V4_34_AVY_NHS_unmut..IGHD" "9"
"V4_34_AVY_NHS_unmut..IGHM" "9"
"V4_34_AVY_NHS_unmut..IGHDM" "9"
"V_gene_freq_by_uniq_VDJ_expanded..IGHV4.34" "9"
"V_gene_freq_by_uniq_VDJ_IGHD..IGHV4.34" "9"
"V_gene_freq_by_uniq_VDJ_IGHD.IGHM_mutated..IGHV4.34" "9"
"V_gene_freq_by_uniq_VDJ_IGHM..IGHV4.34" "9"
"V_gene_freq_by_uniq_VDJ_unexpanded..IGHV4.34" "9"
"V_gene_freq_by_cluster_expanded..IGHV4.34" "9"

"V_gene_freq_by_cluster_IGHD..IGHV4.34" "9"
"V_gene_freq_by_cluster_IGHD.IGHM_mutated..IGHV4.34" "9"
"V_gene_freq_by_cluster_IGHM..IGHV4.34" "9"
"V_gene_freq_by_cluster_unexpanded..IGHV4.34" "9"
"V_gene_freq_by_uniq_VDJ_Class_switched..IGHV3.20" "10"
"V_gene_freq_by_uniq_VDJ_expanded..IGHV3.20" "10"
"V_gene_freq_by_uniq_VDJ_IGHA1..IGHV3.20" "10"
"V_gene_freq_by_uniq_VDJ_IGHD.IGHM_mutated..IGHV3.20" "10"
"V_gene_freq_by_uniq_VDJ_IGHG2..IGHV3.20" "10"
"V_gene_freq_by_uniq_VDJ_IGHG3..IGHV3.20" "10"
"V_gene_freq_by_uniq_VDJ_IGHM..IGHV3.20" "10"
"V_gene_freq_by_uniq_VDJ_unexpanded..IGHV3.20" "10"
"V_gene_freq_by_cluster_Class_switched..IGHV3.20" "10"
"V_gene_freq_by_cluster_expanded..IGHV3.20" "10"
"V_gene_freq_by_cluster_IGHA1..IGHV3.20" "10"
"V_gene_freq_by_cluster_IGHD..IGHV3.20" "10"
"V_gene_freq_by_cluster_IGHD.IGHM_mutated..IGHV3.20" "10"
"V_gene_freq_by_cluster_IGHG2..IGHV3.20" "10"
"V_gene_freq_by_cluster_IGHG3..IGHV3.20" "10"
"V_gene_freq_by_cluster_IGHM..IGHV3.20" "10"
"V_gene_freq_by_cluster_unexpanded..IGHV3.20" "10"
"V_gene_freq_by_uniq_VDJ_Class_switched..IGHV3.30" "11"
"V_gene_freq_by_uniq_VDJ_expanded..IGHV3.30" "11"
"V_gene_freq_by_uniq_VDJ_IGHA1..IGHV3.30" "11"
"V_gene_freq_by_uniq_VDJ_IGHD.IGHM_mutated..IGHV3.30" "11"
"V_gene_freq_by_uniq_VDJ_IGHG2..IGHV3.30" "11"
"V_gene_freq_by_uniq_VDJ_IGHG3..IGHV3.30" "11"
"V_gene_freq_by_uniq_VDJ_IGHM..IGHV3.30" "11"
"V_gene_freq_by_uniq_VDJ_unexpanded..IGHV3.30" "11"
"V_gene_freq_by_cluster_Class_switched..IGHV3.30" "11"
"V_gene_freq_by_cluster_expanded..IGHV3.30" "11"
"V_gene_freq_by_cluster_IGHA1..IGHV3.30" "11"
"V_gene_freq_by_cluster_IGHD..IGHV3.30" "11"
"V_gene_freq_by_cluster_IGHD.IGHM_mutated..IGHV3.30" "11"
"V_gene_freq_by_cluster_IGHG2..IGHV3.30" "11"
"V_gene_freq_by_cluster_IGHG3..IGHV3.30" "11"
"V_gene_freq_by_cluster_IGHM..IGHV3.30" "11"
"V_gene_freq_by_cluster_unexpanded..IGHV3.30" "11"
"J_gene_freq_by_uniq_VDJ_IGHG1..IGHJ2" "12"
"V_gene_freq_by_uniq_VDJ_IGHE..IGHV2.5" "12"
"V_gene_freq_by_uniq_VDJ_IGHE..IGHV2.70" "12"
"V_gene_freq_by_uniq_VDJ_IGHE..IGHV3.43D" "12"
"V_gene_freq_by_uniq_VDJ_IGHG3..IGHV1.24.1" "12"
"V_gene_freq_by_uniq_VDJ_IGHG3..IGHV3.43D.1" "12"
"V_gene_freq_by_cluster_IGHE..IGHV2.70" "12"
"V_gene_freq_by_cluster_IGHE..IGHV3.43D" "12"
"V_gene_freq_by_cluster_IGHG3..IGHV3.43D.1" "12"
"Mean_cluster_size_IGHE..IGHV2.5" "12"
"Mean_cluster_size_IGHE..IGHV2.70" "12"

"Mean_cluster_size_IGHE..IGHV3.43D" "12"
"Mean_cluster_size_IGHG3..IGHV1.24" "12"
"Mean_cluster_size_IGHG3..IGHV2.5" "12"
"Mean_cluster_size_IGHG3..IGHV2.70" "12"
"Mean_cluster_size_IGHG3..IGHV3.43D" "12"
"V_gene_freq_by_uniq_VDJ_Class_switched..IGHV3.74" "13"
"V_gene_freq_by_uniq_VDJ_expanded..IGHV3.74" "13"
"V_gene_freq_by_uniq_VDJ_IGHA1..IGHV3.74" "13"
"V_gene_freq_by_uniq_VDJ_IGHD..IGHV3.74" "13"
"V_gene_freq_by_uniq_VDJ_IGHG2..IGHV3.74" "13"
"V_gene_freq_by_uniq_VDJ_IGHG3..IGHV3.74" "13"
"V_gene_freq_by_uniq_VDJ_IGHM..IGHV3.74" "13"
"V_gene_freq_by_uniq_VDJ_unexpanded..IGHV3.74" "13"
"V_gene_freq_by_cluster_Class_switched..IGHV3.74" "13"
"V_gene_freq_by_cluster_expanded..IGHV3.74" "13"
"V_gene_freq_by_cluster_IGHA1..IGHV3.74" "13"
"V_gene_freq_by_cluster_IGHD..IGHV3.74" "13"
"V_gene_freq_by_cluster_IGHG2..IGHV3.74" "13"
"V_gene_freq_by_cluster_IGHG3..IGHV3.74" "13"
"V_gene_freq_by_cluster_IGHM..IGHV3.74" "13"
"V_gene_freq_by_cluster_unexpanded..IGHV3.74" "13"
"Mean_SHM_per_BCR..IGHM" "14"
"Mean_SHM_per_BCR..unexpanded" "14"
"FWR3_mm..IGHD" "14"
"FWR3_mm..IGHD_IGHM_mutated" "14"
"FWR3_mm..IGHM" "14"
"mean_CDR_FWR_ratio..IGHD" "14"
"mean_CDR_FWR_ratio..IGHD_IGHM_mutated" "14"
"mean_CDR_FWR_ratio..IGHM" "14"
"mean_CDR_mm_per_BCR..IGHD" "14"
"mean_CDR_mm_per_BCR..IGHD_IGHM_mutated" "14"
"mean_CDR_mm_per_BCR..IGHM" "14"
"mean_FWR_mm_per_BCR..IGHD" "14"
"mean_FWR_mm_per_BCR..IGHD_IGHM_mutated" "14"
"mean_FWR_mm_per_BCR..IGHM" "14"
"Mean_mutations_per_BCR..IGHD_IGHM_mutated" "14"
"Mean_mutations_per_BCR..IGHM" "14"
"V_gene_freq_by_uniq_VDJ_Class_switched..IGHV3.72" "15"
"V_gene_freq_by_uniq_VDJ_expanded..IGHV3.72" "15"
"V_gene_freq_by_uniq_VDJ_IGHA1..IGHV3.72" "15"
"V_gene_freq_by_uniq_VDJ_IGHD..IGHV3.72" "15"
"V_gene_freq_by_uniq_VDJ_IGHD.IGHM_mutated..IGHV3.72" "15"
"V_gene_freq_by_uniq_VDJ_IGHG2..IGHV3.72" "15"
"V_gene_freq_by_uniq_VDJ_IGHM..IGHV3.72" "15"
"V_gene_freq_by_uniq_VDJ_unexpanded..IGHV3.72" "15"
"V_gene_freq_by_cluster_Class_switched..IGHV3.72" "15"
"V_gene_freq_by_cluster_expanded..IGHV3.72" "15"
"V_gene_freq_by_cluster_IGHA1..IGHV3.72" "15"
"V_gene_freq_by_cluster_IGHD..IGHV3.72" "15"

"V_gene_freq_by_cluster_IGHD.IGHM_mutated..IGHV3.72" "15"
"V_gene_freq_by_cluster_IGHG2..IGHV3.72" "15"
"V_gene_freq_by_cluster_IGHM..IGHV3.72" "15"
"V_gene_freq_by_cluster_unexpanded..IGHV3.72" "15"
"Percentage_unique_BCRs_per_isotype..IGHD.IGHM_unmutated" "16"
"Percentage_unique_BCRs_per_isotype_group..IGHD" "16"
"Percentage_unique_BCRs_per_isotype_group..IGHM" "16"
"Mean_CDR3_lengths..IGHD" "16"
"Mean_CDR3_lengths..IGHM" "16"
"Percentage_unmutated..Class_switched" "16"
"Percentage_unmutated..expanded" "16"
"Percentage_unmutated..IGHA1" "16"
"Percentage_unmutated..IGHD" "16"
"Percentage_unmutated..IGHG2" "16"
"Percentage_unmutated..IGHM" "16"
"Percentage_unmutated..unexpanded" "16"
"J_gene_freq_by_uniq_VDJ_IGHD.IGHM_mutated..IGHJ6" "16"
"J_gene_freq_by_uniq_VDJ_IGHD.IGHM_unmutated..IGHJ4" "16"
"J_gene_freq_by_uniq_VDJ_IGHD.IGHM_unmutated..IGHJ5" "16"
"J_gene_freq_by_uniq_VDJ_IGHD.IGHM_unmutated..IGHJ6" "16"
"Percentage_unique_BCRs_per_isotype..Class_switched" "17"
"Percentage_unique_BCRs_per_isotype_group..IGHG2" "17"
"Mean_SHM_per_BCR..Class_switched" "17"
"Mean_SHM_per_BCR..expanded" "17"
"Mean_SHM_per_BCR..IGHA1" "17"
"Mean_SHM_per_BCR..IGHD" "17"
"Mean_SHM_per_BCR..IGHG2" "17"
"Mean_SHM_per_BCR..IGHG3" "17"
"FWR3_mm..IGHG2" "17"
"FWR3_mm..IGHG3" "17"
"mean_CDR_mm_per_BCR..IGHG2" "17"
"mean_CDR_mm_per_BCR..IGHG3" "17"
"mean_FWR_mm_per_BCR..IGHG2" "17"
"mean_FWR_mm_per_BCR..IGHG3" "17"
"Mean_mutations_per_BCR..IGHG2" "17"
"Mean_mutations_per_BCR..IGHG3" "17"
"V_gene_freq_by_uniq_VDJ_Class_switched..IGHV2.5" "18"
"V_gene_freq_by_uniq_VDJ_expanded..IGHV2.5" "18"
"V_gene_freq_by_uniq_VDJ_IGHA1..IGHV2.5" "18"
"V_gene_freq_by_uniq_VDJ_IGHD.IGHM_mutated..IGHV2.5" "18"
"V_gene_freq_by_uniq_VDJ_IGHG2..IGHV2.5" "18"
"V_gene_freq_by_uniq_VDJ_IGHG3..IGHV2.5" "18"
"V_gene_freq_by_uniq_VDJ_IGHM..IGHV2.5" "18"
"V_gene_freq_by_uniq_VDJ_unexpanded..IGHV2.5" "18"
"V_gene_freq_by_cluster_Class_switched..IGHV2.5" "18"
"V_gene_freq_by_cluster_expanded..IGHV2.5" "18"
"V_gene_freq_by_cluster_IGHA1..IGHV2.5" "18"
"V_gene_freq_by_cluster_IGHD.IGHM_mutated..IGHV2.5" "18"
"V_gene_freq_by_cluster_IGHG2..IGHV2.5" "18"

"V_gene_freq_by_cluster_IGHG3..IGHV2.5" "18"
"V_gene_freq_by_cluster_IGHM..IGHV2.5" "18"
"V_gene_freq_by_cluster_unexpanded..IGHV2.5" "18"
"Vertex_Gini_Index..IGHE" "19"
"Cluster_Gini_Index..IGHE" "19"
"mean_vertex_size..IGHE" "19"
"Percentage_max_cluster_size..IGHE.1" "19"
"Percentage_max_vertex_size..IGHE.1" "19"
"J_gene_freq_by_uniq_VDJ_IGHE..IGHJ5.1" "19"
"J_gene_freq_by_uniq_VDJ_IGHG3..IGHJ5.1" "19"
"V_gene_freq_by_uniq_VDJ_IGHE..IGHV3.23" "19"
"V_gene_freq_by_uniq_VDJ_IGHE..IGHV3.74" "19"
"V_gene_freq_by_uniq_VDJ_IGHG3..IGHV3.23.1" "19"
"V_gene_freq_by_uniq_VDJ_IGHG3..IGHV3.74.1" "19"
"Mean_cluster_size_IGHE..IGHV3.23" "19"
"Mean_cluster_size_IGHE..IGHV3.74" "19"
"Mean_cluster_size_IGHG3..IGHV3.23.1" "19"
"Mean_cluster_size_IGHG3..IGHV3.74" "19"
"Vertex_Gini_Index..IGHM" "20"
"Cluster_Gini_Index..IGHM" "20"
"mean_vertex_size..IGHM" "20"
"Percentage_max_cluster_size..IGHM" "20"
"Percentage_max_vertex_size..IGHM" "20"
"D5..IGHM.03" "20"
"D10..IGHM.03" "20"
"V_gene_freq_by_cluster_IGHD..IGHV3.49" "20"
"Mean_cluster_size_Class_switched..IGHV4.59" "20"
"Mean_cluster_size_expanded..IGHV4.59" "20"
"Mean_cluster_size_IGHA1..IGHV4.59" "20"
"Mean_cluster_size_IGHD..IGHV3.23" "20"
"Mean_cluster_size_IGHG2..IGHV4.59" "20"
"Mean_cluster_size_IGHM..IGHV3.21" "20"
"Mean_cluster_size_IGHM..IGHV4.59" "20"
"V_gene_freq_by_uniq_VDJ_IGHG2..IGHV6.1" "21"
"V_gene_freq_by_cluster_IGHG2..IGHV6.1" "21"
"Relative_class_switching_absolute..IGHD.01.IGHG3.17" "21"
"D50..IGHG3.17" "21"
"V_gene_freq_by_uniq_VDJ_IGHE..IGHV3.11" "21"
"V_gene_freq_by_uniq_VDJ_IGHE..IGHV3.20" "21"
"V_gene_freq_by_uniq_VDJ_IGHE..IGHV3.64" "21"
"V_gene_freq_by_uniq_VDJ_IGHE..IGHV3.64D" "21"
"V_gene_freq_by_uniq_VDJ_IGHG3..IGHV3.38" "21"
"V_gene_freq_by_cluster_IGHE..IGHV3.20" "21"
"V_gene_freq_by_cluster_IGHE..IGHV3.64D" "21"
"V_gene_freq_by_cluster_IGHG3..IGHV3.38" "21"
"Mean_cluster_size_IGHE..IGHV3.11" "21"
"Mean_cluster_size_IGHE..IGHV3.64" "21"
"Vertex_Gini_Index..all" "22"
"Cluster_Gini_Index..all" "22"

"mean_vertex_size..all" "22"
"Percentage_max_cluster_size..all" "22"
"Percentage_max_vertex_size..all" "22"
"D5..all" "22"
"D10..all" "22"
"V_gene_freq_by_cluster_IGHG3..IGHV3.23" "22"
"Mean_cluster_size_Class_switched..IGHV6.1" "22"
"Mean_cluster_size_expanded..IGHV3.7" "22"
"Mean_cluster_size_expanded..IGHV6.1" "22"
"Mean_cluster_size_IGHA1..IGHV6.1" "22"
"Mean_cluster_size_IGHD.IGHM_mutated..IGHV3.7" "22"
"Mean_cluster_size_IGHG2..IGHV3.23" "22"
"V_gene_freq_by_uniq_VDJ_Class_switched..IGHV1.46" "23"
"V_gene_freq_by_uniq_VDJ_expanded..IGHV1.46" "23"
"V_gene_freq_by_uniq_VDJ_IGHA1..IGHV1.46" "23"
"V_gene_freq_by_uniq_VDJ_IGHD..IGHV1.46" "23"
"V_gene_freq_by_uniq_VDJ_IGHG2..IGHV1.46" "23"
"V_gene_freq_by_uniq_VDJ_IGHG3..IGHV1.46" "23"
"V_gene_freq_by_uniq_VDJ_IGHM..IGHV1.46" "23"
"V_gene_freq_by_uniq_VDJ_unexpanded..IGHV1.46" "23"
"V_gene_freq_by_cluster_Class_switched..IGHV1.46" "23"
"V_gene_freq_by_cluster_expanded..IGHV1.46" "23"
"V_gene_freq_by_cluster_IGHA1..IGHV1.46" "23"
"V_gene_freq_by_cluster_IGHG2..IGHV1.46" "23"
"V_gene_freq_by_cluster_IGHG3..IGHV1.46" "23"
"V_gene_freq_by_cluster_unexpanded..IGHV1.46" "23"
"V_gene_freq_by_uniq_VDJ_Class_switched..IGHV3.73" "24"
"V_gene_freq_by_uniq_VDJ_expanded..IGHV3.73" "24"
"V_gene_freq_by_uniq_VDJ_IGHA1..IGHV3.73" "24"
"V_gene_freq_by_uniq_VDJ_IGHG2..IGHV3.73" "24"
"V_gene_freq_by_uniq_VDJ_IGHG3..IGHV3.73" "24"
"V_gene_freq_by_uniq_VDJ_IGHM..IGHV3.73" "24"
"V_gene_freq_by_uniq_VDJ_unexpanded..IGHV3.73" "24"
"V_gene_freq_by_cluster_Class_switched..IGHV3.73" "24"
"V_gene_freq_by_cluster_expanded..IGHV3.73" "24"
"V_gene_freq_by_cluster_IGHA1..IGHV3.73" "24"
"V_gene_freq_by_cluster_IGHG2..IGHV3.73" "24"
"V_gene_freq_by_cluster_IGHG3..IGHV3.73" "24"
"V_gene_freq_by_cluster_IGHM..IGHV3.73" "24"
"V_gene_freq_by_cluster_unexpanded..IGHV3.73" "24"
"J_gene_freq_by_uniq_VDJ_IGHE..IGHJ3" "25"
"Mean_cluster_size_Class_switched..IGHV3.15" "25"
"Mean_cluster_size_Class_switched..IGHV3.66" "25"
"Mean_cluster_size_Class_switched..IGHV4.34" "25"
"Mean_cluster_size_expanded..IGHV1.2" "25"
"Mean_cluster_size_expanded..IGHV3.43" "25"
"Mean_cluster_size_expanded..IGHV3.66" "25"
"Mean_cluster_size_expanded..IGHV4.34" "25"
"Mean_cluster_size_IGHA1..IGHV4.34" "25"

"Mean_cluster_size_IGHD..IGHV3.21" "25"
"Mean_cluster_size_IGHG2..IGHV1.2" "25"
"Mean_cluster_size_IGHG2..IGHV3.66" "25"
"Mean_cluster_size_IGHG2..IGHV4.34" "25"
"Mean_cluster_size_IGHM..IGHV1.2" "25"
"D5..IGHA1.01" "26"
"D5..IGHG2.03" "26"
"D10..IGHA1.01" "26"
"D10..IGHG2.03" "26"
"D50..IGHA1.01" "26"
"D50..IGHG2.03" "26"
"Mean_cluster_size_Class_switched..IGHV1.2" "26"
"Mean_cluster_size_Class_switched..IGHV3.13" "26"
"Mean_cluster_size_Class_switched..IGHV3.30" "26"
"Mean_cluster_size_Class_switched..IGHV3.53" "26"
"Mean_cluster_size_IGHA1..IGHV3.30" "26"
"Mean_cluster_size_IGHA1..IGHV5.51" "26"
"Mean_cluster_size_IGHG2..IGHV3.30" "26"
"Mean_cluster_size_IGHG2..IGHV3.53" "26"
"V_gene_freq_by_uniq_VDJ_Class_switched..IGHV4.30.4" "27"
"V_gene_freq_by_uniq_VDJ_expanded..IGHV4.30.4" "27"
"V_gene_freq_by_uniq_VDJ_IGHA1..IGHV4.30.2" "27"
"V_gene_freq_by_uniq_VDJ_IGHA1..IGHV4.30.4" "27"
"V_gene_freq_by_uniq_VDJ_IGHG2..IGHV4.30.4" "27"
"V_gene_freq_by_cluster_Class_switched..IGHV4.30.4" "27"
"V_gene_freq_by_cluster_expanded..IGHV4.30.4" "27"
"V_gene_freq_by_cluster_IGHA1..IGHV4.30.2" "27"
"V_gene_freq_by_cluster_IGHA1..IGHV4.30.4" "27"
"V_gene_freq_by_cluster_IGHD..IGHV4.30.2" "27"
"V_gene_freq_by_cluster_IGHG2..IGHV4.30.4" "27"
"V_gene_freq_by_cluster_IGHG3..IGHV4.30.2" "27"
"V_gene_freq_by_cluster_IGHG3..IGHV4.30.4" "27"
"V_gene_freq_by_uniq_VDJ_expanded..IGHV3.64D" "28"
"V_gene_freq_by_uniq_VDJ_IGHD..IGHV3.64D" "28"
"V_gene_freq_by_uniq_VDJ_IGHD.IGHM_mutated..IGHV3.64D" "28"
"V_gene_freq_by_uniq_VDJ_IGHM..IGHV3.64D" "28"
"V_gene_freq_by_uniq_VDJ_unexpanded..IGHV3.64D" "28"
"V_gene_freq_by_cluster_Class_switched..IGHV3.64D" "28"
"V_gene_freq_by_cluster_expanded..IGHV3.64D" "28"
"V_gene_freq_by_cluster_IGHA1..IGHV3.64D" "28"
"V_gene_freq_by_cluster_IGHD..IGHV3.64D" "28"
"V_gene_freq_by_cluster_IGHD.IGHM_mutated..IGHV3.64D" "28"
"V_gene_freq_by_cluster_IGHG2..IGHV3.64D" "28"
"V_gene_freq_by_cluster_IGHM..IGHV3.64D" "28"
"V_gene_freq_by_cluster_unexpanded..IGHV3.64D" "28"
"Mean_cluster_size_Class_switched..IGHV1.18" "29"
"Mean_cluster_size_Class_switched..IGHV3.72" "29"
"Mean_cluster_size_expanded..IGHV1.18" "29"
"Mean_cluster_size_expanded..IGHV3.13" "29"

"Mean_cluster_size_expanded..IGHV3.15" "29"
"Mean_cluster_size_expanded..IGHV3.72" "29"
"Mean_cluster_size_IGHA1..IGHV1.18" "29"
"Mean_cluster_size_IGHA1..IGHV3.15" "29"
"Mean_cluster_size_IGHG2..IGHV1.18" "29"
"Mean_cluster_size_IGHM..IGHV1.18" "29"
"Mean_cluster_size_IGHM..IGHV3.13" "29"
"Mean_cluster_size_IGHM..IGHV3.15" "29"
"Mean_cluster_size_IGHM..IGHV3.72" "29"
"V_gene_freq_by_uniq_VDJ_Class_switched..IGHV4.4" "30"
"V_gene_freq_by_uniq_VDJ_expanded..IGHV4.4" "30"
"V_gene_freq_by_uniq_VDJ_IGHA1..IGHV4.4" "30"
"V_gene_freq_by_uniq_VDJ_IGHD..IGHV4.4" "30"
"V_gene_freq_by_uniq_VDJ_IGHG2..IGHV4.4" "30"
"V_gene_freq_by_uniq_VDJ_IGHG3..IGHV4.4" "30"
"V_gene_freq_by_uniq_VDJ_unexpanded..IGHV4.4" "30"
"V_gene_freq_by_cluster_Class_switched..IGHV4.4" "30"
"V_gene_freq_by_cluster_expanded..IGHV4.4" "30"
"V_gene_freq_by_cluster_IGHA1..IGHV4.4" "30"
"V_gene_freq_by_cluster_IGHG2..IGHV4.4" "30"
"V_gene_freq_by_cluster_IGHG3..IGHV4.4" "30"
"V_gene_freq_by_cluster_unexpanded..IGHV4.4" "30"
"V_gene_freq_by_uniq_VDJ_IGHD..IGHV4.30.2" "31"
"V_gene_freq_by_uniq_VDJ_IGHD..IGHV4.30.4" "31"
"V_gene_freq_by_uniq_VDJ_IGHD.IGHM_mutated..IGHV4.30.2" "31"
"V_gene_freq_by_uniq_VDJ_IGHD.IGHM_mutated..IGHV4.30.4" "31"
"V_gene_freq_by_uniq_VDJ_IGHM..IGHV4.30.2" "31"
"V_gene_freq_by_uniq_VDJ_IGHM..IGHV4.30.4" "31"
"V_gene_freq_by_uniq_VDJ_unexpanded..IGHV4.30.4" "31"
"V_gene_freq_by_cluster_IGHD..IGHV4.30.4" "31"
"V_gene_freq_by_cluster_IGHD.IGHM_mutated..IGHV4.30.2" "31"
"V_gene_freq_by_cluster_IGHD.IGHM_mutated..IGHV4.30.4" "31"
"V_gene_freq_by_cluster_IGHM..IGHV4.30.2" "31"
"V_gene_freq_by_cluster_IGHM..IGHV4.30.4" "31"
"V_gene_freq_by_cluster_unexpanded..IGHV4.30.4" "31"
"V_gene_freq_by_uniq_VDJ_IGHE..IGHV1.46" "32"
"V_gene_freq_by_uniq_VDJ_IGHE..IGHV1.69" "32"
"V_gene_freq_by_uniq_VDJ_IGHG3..IGHV1.46.1" "32"
"V_gene_freq_by_uniq_VDJ_IGHG3..IGHV1.69.1" "32"
"V_gene_freq_by_uniq_VDJ_IGHG3..IGHV5.78" "32"
"V_gene_freq_by_cluster_IGHE..IGHV1.46" "32"
"V_gene_freq_by_cluster_IGHE..IGHV1.69" "32"
"V_gene_freq_by_cluster_IGHG3..IGHV1.46.1" "32"
"V_gene_freq_by_cluster_IGHG3..IGHV5.78" "32"
"Mean_cluster_size_IGHE..IGHV1.69" "32"
"Mean_cluster_size_IGHG3..IGHV1.46" "32"
"Mean_cluster_size_IGHG3..IGHV1.69" "32"
"Percentage_unique_BCRs_per_isotype_group..IGHG1" "33"
"Percentage_unique_BCRs_in_switched..IGHG1" "33"

"V_gene_freq_by_uniq_VDJ_Class_switched..IGHV4.31" "33"
"V_gene_freq_by_uniq_VDJ_expanded..IGHV4.31" "33"
"V_gene_freq_by_uniq_VDJ_IGHA1..IGHV4.31" "33"
"V_gene_freq_by_uniq_VDJ_IGHG2..IGHV4.31" "33"
"V_gene_freq_by_uniq_VDJ_IGHG3..IGHV4.31" "33"
"V_gene_freq_by_cluster_Class_switched..IGHV4.31" "33"
"V_gene_freq_by_cluster_expanded..IGHV4.31" "33"
"V_gene_freq_by_cluster_IGHA1..IGHV4.31" "33"
"V_gene_freq_by_cluster_IGHG2..IGHV4.31" "33"
"V_gene_freq_by_cluster_IGHG3..IGHV4.31" "33"
"V_gene_freq_by_uniq_VDJ_Class_switched..IGHV3.13" "34"
"V_gene_freq_by_uniq_VDJ_expanded..IGHV3.13" "34"
"V_gene_freq_by_uniq_VDJ_IGHG2..IGHV3.13" "34"
"V_gene_freq_by_uniq_VDJ_IGHG3..IGHV3.13" "34"
"V_gene_freq_by_uniq_VDJ_IGHM..IGHV3.13" "34"
"V_gene_freq_by_uniq_VDJ_unexpanded..IGHV3.13" "34"
"V_gene_freq_by_cluster_Class_switched..IGHV3.13" "34"
"V_gene_freq_by_cluster_expanded..IGHV3.13" "34"
"V_gene_freq_by_cluster_IGHA1..IGHV3.13" "34"
"V_gene_freq_by_cluster_IGHG2..IGHV3.13" "34"
"V_gene_freq_by_cluster_IGHM..IGHV3.13" "34"
"V_gene_freq_by_cluster_unexpanded..IGHV3.13" "34"
"Vertex_Reyni..IGHE" "35"
"Cluster_Reyni..IGHE" "35"
"mean_CDR3_charge..IGHE" "35"
"V_gene_freq_by_cluster_IGHE..IGHV3.21" "35"
"V_gene_freq_by_cluster_IGHE..IGHV3.23" "35"
"V_gene_freq_by_cluster_IGHE..IGHV3.30" "35"
"V_gene_freq_by_cluster_IGHE..IGHV3.33" "35"
"V_gene_freq_by_cluster_IGHE..IGHV3.48" "35"
"V_gene_freq_by_cluster_IGHE..IGHV3.64" "35"
"V_gene_freq_by_cluster_IGHE..IGHV3.66" "35"
"V_gene_freq_by_cluster_IGHE..IGHV3.7" "35"
"V_gene_freq_by_cluster_IGHE..IGHV3.74" "35"
"V_gene_freq_by_uniq_VDJ_Class_switched..IGHV3.NL1" "36"
"V_gene_freq_by_uniq_VDJ_expanded..IGHV3.NL1" "36"
"V_gene_freq_by_uniq_VDJ_IGHA1..IGHV3.NL1" "36"
"V_gene_freq_by_uniq_VDJ_IGHG2..IGHV3.NL1" "36"
"V_gene_freq_by_uniq_VDJ_IGHM..IGHV3.NL1" "36"
"V_gene_freq_by_uniq_VDJ_unexpanded..IGHV3.NL1" "36"
"V_gene_freq_by_cluster_Class_switched..IGHV3.NL1" "36"
"V_gene_freq_by_cluster_expanded..IGHV3.NL1" "36"
"V_gene_freq_by_cluster_IGHA1..IGHV3.NL1" "36"
"V_gene_freq_by_cluster_IGHG2..IGHV3.NL1" "36"
"V_gene_freq_by_cluster_IGHM..IGHV3.NL1" "36"
"V_gene_freq_by_cluster_unexpanded..IGHV3.NL1" "36"
"Mean_cluster_size_Class_switched..IGHV2.5" "37"
"Mean_cluster_size_Class_switched..IGHV3.74" "37"
"Mean_cluster_size_expanded..IGHV2.5" "37"

"Mean_cluster_size_expanded..IGHV3.74" "37"
"Mean_cluster_size_IGHA1..IGHV2.5" "37"
"Mean_cluster_size_IGHA1..IGHV3.74" "37"
"Mean_cluster_size_IGHG2..IGHV2.5" "37"
"Mean_cluster_size_IGHG2..IGHV3.15" "37"
"Mean_cluster_size_IGHG2..IGHV3.74" "37"
"Mean_cluster_size_IGHM..IGHV2.5" "37"
"Mean_cluster_size_IGHM..IGHV3.66" "37"
"Mean_cluster_size_IGHM..IGHV6.1" "37"
"Mean_cluster_size_unexpanded..IGHV2.5" "38"
"Mean_cluster_size_unexpanded..IGHV3.11" "38"
"Mean_cluster_size_unexpanded..IGHV3.21" "38"
"Mean_cluster_size_unexpanded..IGHV3.23" "38"
"Mean_cluster_size_unexpanded..IGHV3.30" "38"
"Mean_cluster_size_unexpanded..IGHV3.33" "38"
"Mean_cluster_size_unexpanded..IGHV3.48" "38"
"Mean_cluster_size_unexpanded..IGHV3.53" "38"
"Mean_cluster_size_unexpanded..IGHV3.7" "38"
"Mean_cluster_size_unexpanded..IGHV3.74" "38"
"Mean_cluster_size_unexpanded..IGHV4.34" "38"
"Mean_cluster_size_unexpanded..IGHV4.59" "38"
"J_gene_freq_by_uniq_VDJ_Class_switched..IGHJ4" "39"
"J_gene_freq_by_uniq_VDJ_expanded..IGHJ1" "39"
"J_gene_freq_by_uniq_VDJ_expanded..IGHJ4" "39"
"J_gene_freq_by_uniq_VDJ_IGHA1..IGHJ4" "39"
"J_gene_freq_by_uniq_VDJ_IGHD.IGHM_mutated..IGHJ4" "39"
"J_gene_freq_by_uniq_VDJ_IGHG2..IGHJ1" "39"
"J_gene_freq_by_uniq_VDJ_IGHG2..IGHJ4" "39"
"J_gene_freq_by_uniq_VDJ_IGHG3..IGHJ4" "39"
"J_gene_freq_by_uniq_VDJ_IGHM..IGHJ1" "39"
"J_gene_freq_by_uniq_VDJ_IGHM..IGHJ4" "39"
"J_gene_freq_by_uniq_VDJ_unexpanded..IGHJ1" "39"
"J_gene_freq_by_uniq_VDJ_unexpanded..IGHJ4" "39"
"V4_34_NHS_unmut..IGHG3" "40"
"V4_34_AVY_NHS_unmut..IGHG3" "40"
"V_gene_freq_by_uniq_VDJ_IGHD..IGHV4.28" "40"
"V_gene_freq_by_uniq_VDJ_IGHD..IGHV4.61" "40"
"V_gene_freq_by_uniq_VDJ_IGHD.IGHM_mutated..IGHV4.61" "40"
"V_gene_freq_by_uniq_VDJ_IGHG3..IGHV4.34" "40"
"V_gene_freq_by_cluster_IGHD..IGHV4.28" "40"
"V_gene_freq_by_cluster_IGHD..IGHV4.61" "40"
"V_gene_freq_by_cluster_IGHD.IGHM_mutated..IGHV4.61" "40"
"V_gene_freq_by_cluster_IGHG3..IGHV4.34" "40"
"V_gene_freq_by_uniq_VDJ_IGHE..IGHV3.21" "40"
"Mean_cluster_size_IGHE..IGHV3.21" "40"
"Relative_class_switching_absolute..IGHG2.03.IGHG3.17" "41"
"V_gene_freq_by_uniq_VDJ_IGHD.IGHM_mutated..IGHV1.46" "41"
"V_gene_freq_by_cluster_IGHD.IGHM_mutated..IGHV1.46" "41"
"V_gene_freq_by_cluster_IGHM..IGHV1.46" "41"

"V4_34_AVY_unmut..IGHE" "41"
"V4_34_NHS_unmut..IGHE" "41"
"V4_34_AVY_NHS_unmut..IGHE" "41"
"J_gene_freq_by_uniq_VDJ_IGHE..IGHJ1.1" "41"
"V_gene_freq_by_uniq_VDJ_IGHE..IGHV3.15" "41"
"V_gene_freq_by_uniq_VDJ_IGHG3..IGHV2.26" "41"
"V_gene_freq_by_cluster_IGHG3..IGHV2.26" "41"
"V_gene_freq_by_uniq_VDJ_Class_switched..IGHV3.48" "42"
"V_gene_freq_by_uniq_VDJ_expanded..IGHV3.48" "42"
"V_gene_freq_by_uniq_VDJ_IGHD..IGHV3.48" "42"
"V_gene_freq_by_uniq_VDJ_IGHD.IGHM_mutated..IGHV3.48" "42"
"V_gene_freq_by_uniq_VDJ_IGHM..IGHV3.48" "42"
"V_gene_freq_by_uniq_VDJ_unexpanded..IGHV3.48" "42"
"V_gene_freq_by_cluster_expanded..IGHV3.48" "42"
"V_gene_freq_by_cluster_IGHD..IGHV3.48" "42"
"V_gene_freq_by_cluster_IGHD.IGHM_mutated..IGHV3.48" "42"
"V_gene_freq_by_cluster_IGHM..IGHV3.48" "42"
"V_gene_freq_by_cluster_unexpanded..IGHV3.48" "42"
"V_gene_freq_by_uniq_VDJ_Class_switched..IGHV3.69.1" "43"
"V_gene_freq_by_uniq_VDJ_Class_switched..IGHV5.10.1" "43"
"V_gene_freq_by_uniq_VDJ_expanded..IGHV5.10.1" "43"
"V_gene_freq_by_uniq_VDJ_IGHG2..IGHV5.10.1" "43"
"V_gene_freq_by_uniq_VDJ_IGHG3..IGHV5.10.1" "43"
"V_gene_freq_by_cluster_Class_switched..IGHV3.69.1" "43"
"V_gene_freq_by_cluster_Class_switched..IGHV5.10.1" "43"
"V_gene_freq_by_cluster_expanded..IGHV5.10.1" "43"
"V_gene_freq_by_cluster_IGHA1..IGHV5.10.1" "43"
"V_gene_freq_by_cluster_IGHG2..IGHV5.10.1" "43"
"V_gene_freq_by_cluster_IGHG3..IGHV5.10.1" "43"
"V_gene_freq_by_uniq_VDJ_IGHE..IGHV4.31" "44"
"V_gene_freq_by_uniq_VDJ_IGHE..IGHV4.39" "44"
"V_gene_freq_by_cluster_IGHE..IGHV2.5" "44"
"V_gene_freq_by_cluster_IGHE..IGHV4.30.2" "44"
"V_gene_freq_by_cluster_IGHE..IGHV4.31" "44"
"V_gene_freq_by_cluster_IGHE..IGHV4.34" "44"
"V_gene_freq_by_cluster_IGHE..IGHV4.39" "44"
"V_gene_freq_by_cluster_IGHE..IGHV4.4" "44"
"V_gene_freq_by_cluster_IGHE..IGHV4.59" "44"
"V_gene_freq_by_cluster_IGHE..IGHV4.61" "44"
"Mean_cluster_size_IGHE..IGHV4.39" "44"
"V_gene_freq_by_uniq_VDJ_expanded..IGHV1.69" "45"
"V_gene_freq_by_uniq_VDJ_IGHD..IGHV1.69" "45"
"V_gene_freq_by_uniq_VDJ_IGHD.IGHM_mutated..IGHV1.69" "45"
"V_gene_freq_by_uniq_VDJ_IGHM..IGHV1.69" "45"
"V_gene_freq_by_uniq_VDJ_unexpanded..IGHV1.69" "45"
"V_gene_freq_by_cluster_expanded..IGHV1.24" "45"
"V_gene_freq_by_cluster_IGHD..IGHV1.69" "45"
"V_gene_freq_by_cluster_IGHD.IGHM_mutated..IGHV1.69" "45"
"V_gene_freq_by_cluster_IGHM..IGHV1.24" "45"

"V_gene_freq_by_cluster_IGHM..IGHV1.69" "45"
"V_gene_freq_by_cluster_unexpanded..IGHV1.69" "45"
"V4_34_AVY_unmut..IGHG3.1" "46"
"V_gene_freq_by_uniq_VDJ_IGHE..IGHV3.69.1" "46"
"V_gene_freq_by_uniq_VDJ_IGHE..IGHV5.10.1" "46"
"V_gene_freq_by_uniq_VDJ_IGHG3..IGHV3.69.1.1" "46"
"V_gene_freq_by_uniq_VDJ_IGHG3..IGHV5.10.1.1" "46"
"V_gene_freq_by_cluster_IGHG3..IGHV3.69.1.1" "46"
"V_gene_freq_by_cluster_IGHG3..IGHV5.10.1.1" "46"
"Mean_cluster_size_IGHE..IGHV5.10.1" "46"
"Mean_cluster_size_IGHG3..IGHV3.69.1" "46"
"Mean_cluster_size_IGHG3..IGHV5.10.1" "46"
"J_gene_freq_by_uniq_VDJ_IGHD..IGHJ1" "47"
"V_gene_freq_by_uniq_VDJ_Class_switched..IGHV3.71" "47"
"V_gene_freq_by_uniq_VDJ_IGHG2..IGHV3.71" "47"
"V_gene_freq_by_uniq_VDJ_IGHM..IGHV3.71" "47"
"V_gene_freq_by_cluster_Class_switched..IGHV3.71" "47"
"V_gene_freq_by_cluster_expanded..IGHV3.71" "47"
"V_gene_freq_by_cluster_IGHG2..IGHV3.71" "47"
"V_gene_freq_by_cluster_IGHM..IGHV3.71" "47"
"V_gene_freq_by_uniq_VDJ_IGHG3..IGHV3.35" "47"
"V_gene_freq_by_cluster_IGHG3..IGHV3.35" "47"
"Mean_CDR3_lengths..IGHE" "48"
"FWR3_mm..IGHE" "48"
"mean_CDR_FWR_ratio..IGHE" "48"
"mean_CDR_mm_per_BCR..IGHE" "48"
"mean_FWR_mm_per_BCR..IGHE" "48"
"Mean_mutations_per_BCR..IGHE.1" "48"
"D5..IGHE.02" "48"
"D10..IGHE.02" "48"
"V_gene_freq_by_cluster_IGHE..IGHV3.11" "48"
"V_gene_freq_by_cluster_IGHE..IGHV3.53" "48"
"V_gene_freq_by_uniq_VDJ_expanded..IGHV3.33" "49"
"V_gene_freq_by_uniq_VDJ_IGHD..IGHV3.33" "49"
"V_gene_freq_by_uniq_VDJ_IGHD.IGHM_mutated..IGHV3.33" "49"
"V_gene_freq_by_uniq_VDJ_IGHM..IGHV3.33" "49"
"V_gene_freq_by_uniq_VDJ_unexpanded..IGHV3.33" "49"
"V_gene_freq_by_cluster_expanded..IGHV3.33" "49"
"V_gene_freq_by_cluster_IGHD..IGHV3.33" "49"
"V_gene_freq_by_cluster_IGHD.IGHM_mutated..IGHV3.33" "49"
"V_gene_freq_by_cluster_IGHM..IGHV3.33" "49"
"V_gene_freq_by_cluster_unexpanded..IGHV3.33" "49"
"V_gene_freq_by_uniq_VDJ_expanded..IGHV4.38.2" "50"
"V_gene_freq_by_uniq_VDJ_IGHD..IGHV4.38.2" "50"
"V_gene_freq_by_uniq_VDJ_IGHD.IGHM_mutated..IGHV4.38.2" "50"
"V_gene_freq_by_uniq_VDJ_IGHM..IGHV4.38.2" "50"
"V_gene_freq_by_uniq_VDJ_unexpanded..IGHV4.38.2" "50"
"V_gene_freq_by_cluster_expanded..IGHV4.38.2" "50"
"V_gene_freq_by_cluster_IGHD..IGHV4.38.2" "50"

"V_gene_freq_by_cluster_IGHD.IGHM_mutated..IGHV4.38.2" "50"
"V_gene_freq_by_cluster_IGHM..IGHV4.38.2" "50"
"V_gene_freq_by_cluster_unexpanded..IGHV4.38.2" "50"
"Vertex_Gini_Index..IGHD" "51"
"Cluster_Gini_Index..IGHD" "51"
"mean_vertex_size..IGHD" "51"
"D5..IGHD.01" "51"
"D10..IGHD.01" "51"
"D50..all" "51"
"D50..IGHM.03" "51"
"Mean_cluster_size_IGHA1..IGHV1.2" "51"
"Mean_cluster_size_IGHD..IGHV3.48" "51"
"Mean_cluster_size_IGHD..IGHV3.7" "51"
"V_gene_freq_by_uniq_VDJ_expanded..IGHV3.30.3" "52"
"V_gene_freq_by_uniq_VDJ_IGHD..IGHV3.30.3" "52"
"V_gene_freq_by_uniq_VDJ_IGHD.IGHM_mutated..IGHV3.30.3" "52"
"V_gene_freq_by_uniq_VDJ_IGHM..IGHV3.30.3" "52"
"V_gene_freq_by_uniq_VDJ_unexpanded..IGHV3.30.3" "52"
"V_gene_freq_by_cluster_expanded..IGHV3.30.3" "52"
"V_gene_freq_by_cluster_IGHD..IGHV3.30.3" "52"
"V_gene_freq_by_cluster_IGHD.IGHM_mutated..IGHV3.30.3" "52"
"V_gene_freq_by_cluster_IGHM..IGHV3.30.3" "52"
"V_gene_freq_by_cluster_unexpanded..IGHV3.30.3" "52"
"V_gene_freq_by_uniq_VDJ_Class_switched..IGHV3.7" "53"
"V_gene_freq_by_uniq_VDJ_expanded..IGHV3.7" "53"
"V_gene_freq_by_uniq_VDJ_IGHA1..IGHV3.7" "53"
"V_gene_freq_by_uniq_VDJ_IGHG2..IGHV3.7" "53"
"V_gene_freq_by_uniq_VDJ_IGHG3..IGHV3.7" "53"
"V_gene_freq_by_uniq_VDJ_unexpanded..IGHV3.7" "53"
"V_gene_freq_by_cluster_Class_switched..IGHV3.7" "53"
"V_gene_freq_by_cluster_IGHA1..IGHV3.7" "53"
"V_gene_freq_by_cluster_IGHG2..IGHV3.7" "53"
"V_gene_freq_by_cluster_unexpanded..IGHV3.7" "53"
"V_gene_freq_by_uniq_VDJ_Class_switched..IGHV4.55" "54"
"V_gene_freq_by_uniq_VDJ_expanded..IGHV4.55" "54"
"V_gene_freq_by_uniq_VDJ_IGHA1..IGHV4.55" "54"
"V_gene_freq_by_uniq_VDJ_IGHG2..IGHV4.55" "54"
"V_gene_freq_by_uniq_VDJ_IGHM..IGHV4.55" "54"
"V_gene_freq_by_cluster_Class_switched..IGHV4.55" "54"
"V_gene_freq_by_cluster_expanded..IGHV4.55" "54"
"V_gene_freq_by_cluster_IGHA1..IGHV4.55" "54"
"V_gene_freq_by_cluster_IGHG2..IGHV4.55" "54"
"V_gene_freq_by_cluster_IGHM..IGHV4.55" "54"
"V_gene_freq_by_uniq_VDJ_expanded..IGHV3.43D" "55"
"V_gene_freq_by_uniq_VDJ_IGHD..IGHV3.43D" "55"
"V_gene_freq_by_uniq_VDJ_IGHD.IGHM_mutated..IGHV3.43D" "55"
"V_gene_freq_by_uniq_VDJ_IGHM..IGHV3.43D" "55"
"V_gene_freq_by_uniq_VDJ_unexpanded..IGHV3.43D" "55"
"V_gene_freq_by_cluster_expanded..IGHV3.43D" "55"

"V_gene_freq_by_cluster_IGHD..IGHV3.43D" "55"
"V_gene_freq_by_cluster_IGHD.IGHM_mutated..IGHV3.43D" "55"
"V_gene_freq_by_cluster_IGHM..IGHV3.43D" "55"
"V_gene_freq_by_cluster_unexpanded..IGHV3.43D" "55"
"V_gene_freq_by_uniq_VDJ_expanded..IGHV1.18" "56"
"V_gene_freq_by_uniq_VDJ_IGHD..IGHV1.18" "56"
"V_gene_freq_by_uniq_VDJ_IGHD.IGHM_mutated..IGHV1.18" "56"
"V_gene_freq_by_uniq_VDJ_IGHM..IGHV1.18" "56"
"V_gene_freq_by_uniq_VDJ_unexpanded..IGHV1.18" "56"
"V_gene_freq_by_cluster_expanded..IGHV1.18" "56"
"V_gene_freq_by_cluster_IGHD..IGHV1.18" "56"
"V_gene_freq_by_cluster_IGHD.IGHM_mutated..IGHV1.18" "56"
"V_gene_freq_by_cluster_IGHM..IGHV1.18" "56"
"V_gene_freq_by_cluster_unexpanded..IGHV1.18" "56"
"V_gene_freq_by_uniq_VDJ_expanded..IGHV1.8" "57"
"V_gene_freq_by_uniq_VDJ_IGHD..IGHV1.8" "57"
"V_gene_freq_by_uniq_VDJ_IGHD.IGHM_mutated..IGHV1.8" "57"
"V_gene_freq_by_uniq_VDJ_IGHM..IGHV1.8" "57"
"V_gene_freq_by_uniq_VDJ_unexpanded..IGHV1.8" "57"
"V_gene_freq_by_cluster_expanded..IGHV1.8" "57"
"V_gene_freq_by_cluster_IGHD..IGHV1.8" "57"
"V_gene_freq_by_cluster_IGHD.IGHM_mutated..IGHV1.8" "57"
"V_gene_freq_by_cluster_IGHM..IGHV1.8" "57"
"V_gene_freq_by_cluster_unexpanded..IGHV1.8" "57"
"V_gene_freq_by_uniq_VDJ_expanded..IGHV1.2" "58"
"V_gene_freq_by_uniq_VDJ_IGHD..IGHV1.2" "58"
"V_gene_freq_by_uniq_VDJ_IGHD.IGHM_mutated..IGHV1.2" "58"
"V_gene_freq_by_uniq_VDJ_IGHM..IGHV1.2" "58"
"V_gene_freq_by_uniq_VDJ_unexpanded..IGHV1.2" "58"
"V_gene_freq_by_cluster_expanded..IGHV1.2" "58"
"V_gene_freq_by_cluster_IGHD..IGHV1.2" "58"
"V_gene_freq_by_cluster_IGHD.IGHM_mutated..IGHV1.2" "58"
"V_gene_freq_by_cluster_IGHM..IGHV1.2" "58"
"V_gene_freq_by_cluster_unexpanded..IGHV1.2" "58"
"V_gene_freq_by_uniq_VDJ_IGHD..IGHV3.30" "59"
"V_gene_freq_by_uniq_VDJ_IGHD..IGHV3.NL1" "59"
"Mean_cluster_size_Class_switched..IGHV4.39" "59"
"Mean_cluster_size_Class_switched..IGHV4.4" "59"
"Mean_cluster_size_expanded..IGHV4.39" "59"
"Mean_cluster_size_expanded..IGHV4.4" "59"
"Mean_cluster_size_IGHA1..IGHV4.39" "59"
"Mean_cluster_size_IGHG2..IGHV4.39" "59"
"Mean_cluster_size_IGHM..IGHV3.30" "59"
"Mean_cluster_size_IGHM..IGHV4.39" "59"
"V_gene_freq_by_uniq_VDJ_IGHM..IGHV3.7" "60"
"Mean_cluster_size_Class_switched..IGHV3.7" "60"
"Mean_cluster_size_Class_switched..IGHV4.61" "60"
"Mean_cluster_size_expanded..IGHV4.61" "60"
"Mean_cluster_size_IGHA1..IGHV4.61" "60"

"Mean_cluster_size_IGHG2..IGHV3.7" "60"
"Mean_cluster_size_IGHG2..IGHV4.61" "60"
"Mean_cluster_size_IGHM..IGHV3.7" "60"
"Mean_cluster_size_IGHM..IGHV3.74" "60"
"Mean_cluster_size_IGHM..IGHV4.61" "60"
"Mean_CDR3_lengths..IGHA1" "61"
"Mean_CDR3_lengths..IGHG2" "61"
"FWR3_mm..IGHA1" "61"
"mean_CDR_FWR_ratio..IGHA1" "61"
"mean_CDR_FWR_ratio..IGHG2" "61"
"mean_CDR_mm_per_BCR..IGHA1" "61"
"mean_FWR_mm_per_BCR..IGHA1" "61"
"Mean_mutations_per_BCR..IGHA1" "61"
"mean_CDR3_charge..IGHA1" "61"
"mean_CDR3_charge..IGHG2" "61"
"V_gene_freq_by_uniq_VDJ_expanded..IGHV5.51" "62"
"V_gene_freq_by_uniq_VDJ_IGHD..IGHV5.51" "62"
"V_gene_freq_by_uniq_VDJ_IGHD.IGHM_mutated..IGHV5.51" "62"
"V_gene_freq_by_uniq_VDJ_IGHM..IGHV5.51" "62"
"V_gene_freq_by_uniq_VDJ_unexpanded..IGHV5.51" "62"
"V_gene_freq_by_cluster_expanded..IGHV5.51" "62"
"V_gene_freq_by_cluster_IGHD..IGHV5.51" "62"
"V_gene_freq_by_cluster_IGHD.IGHM_mutated..IGHV5.51" "62"
"V_gene_freq_by_cluster_IGHM..IGHV5.51" "62"
"V_gene_freq_by_cluster_unexpanded..IGHV5.51" "62"
"V_gene_freq_by_uniq_VDJ_expanded..IGHV2.26" "63"
"V_gene_freq_by_uniq_VDJ_IGHD..IGHV2.26" "63"
"V_gene_freq_by_uniq_VDJ_IGHD.IGHM_mutated..IGHV2.26" "63"
"V_gene_freq_by_uniq_VDJ_IGHM..IGHV2.26" "63"
"V_gene_freq_by_uniq_VDJ_unexpanded..IGHV2.26" "63"
"V_gene_freq_by_cluster_expanded..IGHV2.26" "63"
"V_gene_freq_by_cluster_IGHD..IGHV2.26" "63"
"V_gene_freq_by_cluster_IGHD.IGHM_mutated..IGHV2.26" "63"
"V_gene_freq_by_cluster_IGHM..IGHV2.26" "63"
"V_gene_freq_by_cluster_unexpanded..IGHV2.26" "63"
"V_gene_freq_by_uniq_VDJ_expanded..IGHV3.23" "64"
"V_gene_freq_by_uniq_VDJ_IGHD..IGHV3.23" "64"
"V_gene_freq_by_uniq_VDJ_IGHD.IGHM_mutated..IGHV3.23" "64"
"V_gene_freq_by_uniq_VDJ_IGHM..IGHV3.23" "64"
"V_gene_freq_by_uniq_VDJ_unexpanded..IGHV3.23" "64"
"V_gene_freq_by_cluster_expanded..IGHV3.23" "64"
"V_gene_freq_by_cluster_IGHD..IGHV3.23" "64"
"V_gene_freq_by_cluster_IGHD.IGHM_mutated..IGHV3.23" "64"
"V_gene_freq_by_cluster_IGHM..IGHV3.23" "64"
"V_gene_freq_by_cluster_unexpanded..IGHV3.23" "64"
"V_gene_freq_by_uniq_VDJ_IGHD.IGHM_mutated..IGHV3.52" "65"
"V_gene_freq_by_uniq_VDJ_IGHM..IGHV3.52" "65"
"V_gene_freq_by_uniq_VDJ_unexpanded..IGHV3.52" "65"
"V_gene_freq_by_cluster_IGHD.IGHM_mutated..IGHV3.52" "65"

"V_gene_freq_by_cluster_IGHM..IGHV3.52" "65"
"V_gene_freq_by_cluster_unexpanded..IGHV3.52" "65"
"V_gene_freq_by_uniq_VDJ_IGHE..IGHV3.48" "65"
"V_gene_freq_by_uniq_VDJ_IGHG3..IGHV3.38.3" "65"
"V_gene_freq_by_cluster_IGHG3..IGHV3.38.3" "65"
"Mean_cluster_size_IGHE..IGHV3.48" "65"
"V_gene_freq_by_uniq_VDJ_Class_switched..IGHV3.15" "66"
"V_gene_freq_by_uniq_VDJ_expanded..IGHV3.15" "66"
"V_gene_freq_by_uniq_VDJ_IGHA1..IGHV3.15" "66"
"V_gene_freq_by_uniq_VDJ_IGHG2..IGHV3.15" "66"
"V_gene_freq_by_uniq_VDJ_IGHG3..IGHV3.15" "66"
"V_gene_freq_by_uniq_VDJ_IGHM..IGHV3.15" "66"
"V_gene_freq_by_cluster_Class_switched..IGHV3.15" "66"
"V_gene_freq_by_cluster_IGHA1..IGHV3.15" "66"
"V_gene_freq_by_cluster_IGHG2..IGHV3.15" "66"
"V_gene_freq_by_cluster_IGHG3..IGHV3.15" "66"
"V_gene_freq_by_uniq_VDJ_Class_switched..IGHV5.51" "67"
"V_gene_freq_by_uniq_VDJ_IGHG2..IGHV5.51" "67"
"Mean_cluster_size_Class_switched..IGHV5.51" "67"
"Mean_cluster_size_IGHD.IGHM_mutated..IGHV3.23" "67"
"Mean_cluster_size_IGHD.IGHM_mutated..IGHV3.30" "67"
"Mean_cluster_size_IGHD.IGHM_mutated..IGHV3.33" "67"
"Mean_cluster_size_IGHD.IGHM_mutated..IGHV3.48" "67"
"Mean_cluster_size_IGHD.IGHM_mutated..IGHV4.39" "67"
"Mean_cluster_size_IGHD.IGHM_mutated..IGHV4.59" "67"
"Mean_cluster_size_IGHG2..IGHV5.51" "67"
"V_gene_freq_by_uniq_VDJ_IGHE..IGHV3.7" "68"
"V_gene_freq_by_uniq_VDJ_IGHE..IGHV4.61" "68"
"V_gene_freq_by_uniq_VDJ_IGHG3..IGHV3.7.1" "68"
"V_gene_freq_by_uniq_VDJ_IGHG3..IGHV4.61.1" "68"
"V_gene_freq_by_cluster_IGHE..IGHV1.8" "68"
"V_gene_freq_by_cluster_IGHE..IGHV3.15" "68"
"Mean_cluster_size_IGHE..IGHV3.7" "68"
"Mean_cluster_size_IGHE..IGHV4.61" "68"
"Mean_cluster_size_IGHG3..IGHV3.7.1" "68"
"Mean_cluster_size_IGHG3..IGHV4.61" "68"
"V_gene_freq_by_uniq_VDJ_IGHD.IGHM_mutated..IGHV3.43" "69"
"V_gene_freq_by_uniq_VDJ_unexpanded..IGHV3.43" "69"
"V_gene_freq_by_cluster_Class_switched..IGHV3.43" "69"
"V_gene_freq_by_cluster_expanded..IGHV3.43" "69"
"V_gene_freq_by_cluster_IGHA1..IGHV3.43" "69"
"V_gene_freq_by_cluster_IGHD.IGHM_mutated..IGHV3.43" "69"
"V_gene_freq_by_cluster_IGHG2..IGHV3.43" "69"
"V_gene_freq_by_cluster_IGHM..IGHV3.43" "69"
"V_gene_freq_by_cluster_unexpanded..IGHV3.43" "69"
"V_gene_freq_by_uniq_VDJ_Class_switched..IGHV3.43" "70"
"V_gene_freq_by_uniq_VDJ_expanded..IGHV3.43" "70"
"V_gene_freq_by_uniq_VDJ_IGHA1..IGHV3.43" "70"
"V_gene_freq_by_uniq_VDJ_IGHG2..IGHV3.43" "70"

"V_gene_freq_by_uniq_VDJ_IGHG3..IGHV3.43" "70"
"V_gene_freq_by_uniq_VDJ_IGHM..IGHV3.43" "70"
"V_gene_freq_by_cluster_IGHG3..IGHV3.43" "70"
"V_gene_freq_by_uniq_VDJ_IGHE..IGHV3.30" "70"
"Mean_cluster_size_IGHE..IGHV3.30" "70"
"Mean_cluster_size_Class_switched..IGHV3.23" "71"
"Mean_cluster_size_Class_switched..IGHV3.33" "71"
"Mean_cluster_size_expanded..IGHV3.23" "71"
"Mean_cluster_size_expanded..IGHV3.33" "71"
"Mean_cluster_size_IGHA1..IGHV3.23" "71"
"Mean_cluster_size_IGHA1..IGHV3.33" "71"
"Mean_cluster_size_IGHG2..IGHV3.33" "71"
"Mean_cluster_size_IGHG3..IGHV3.23" "71"
"Mean_cluster_size_IGHM..IGHV3.33" "71"
"V_gene_freq_by_uniq_VDJ_expanded..IGHV3.11" "72"
"V_gene_freq_by_uniq_VDJ_IGHD.IGHM_mutated..IGHV3.11" "72"
"V_gene_freq_by_uniq_VDJ_IGHM..IGHV3.11" "72"
"V_gene_freq_by_uniq_VDJ_unexpanded..IGHV3.11" "72"
"V_gene_freq_by_cluster_expanded..IGHV3.11" "72"
"V_gene_freq_by_cluster_IGHD..IGHV3.11" "72"
"V_gene_freq_by_cluster_IGHD.IGHM_mutated..IGHV3.11" "72"
"V_gene_freq_by_cluster_IGHM..IGHV3.11" "72"
"V_gene_freq_by_cluster_unexpanded..IGHV3.11" "72"
"Percentage_max_cluster_size..IGHE" "73"
"Percentage_max_vertex_size..IGHE" "73"
"Percentage_unique_BCRs_per_isotype_group..IGHE" "73"
"Relative_class_switching_absolute..IGHA1.01.IGHE.02" "73"
"Relative_class_switching_absolute..IGHE.02.IGHG2.03" "73"
"Mean_mutations_per_BCR..IGHE" "73"
"Relative_class_switching_normalised..IGHA1.IGHE" "73"
"Relative_class_switching_normalised..IGHD.M.IGHE" "73"
"Relative_class_switching_normalised..IGHE.IGHG2" "73"
"J_gene_freq_by_uniq_VDJ_IGHE..IGHJ3.1" "74"
"V_gene_freq_by_uniq_VDJ_IGHE..IGHV1.2" "74"
"V_gene_freq_by_uniq_VDJ_IGHG3..IGHV1.2.1" "74"
"V_gene_freq_by_uniq_VDJ_IGHG3..IGHV3.43.1" "74"
"V_gene_freq_by_cluster_IGHG3..IGHV1.2.1" "74"
"V_gene_freq_by_cluster_IGHG3..IGHV3.43.1" "74"
"Mean_cluster_size_IGHE..IGHV1.2" "74"
"Mean_cluster_size_IGHG3..IGHV1.2" "74"
"Mean_cluster_size_IGHG3..IGHV3.43" "74"
"V_gene_freq_by_uniq_VDJ_IGHE..IGHV1.69.2" "75"
"V_gene_freq_by_uniq_VDJ_IGHE..IGHV3.73" "75"
"V_gene_freq_by_uniq_VDJ_IGHE..IGHV3.9" "75"
"V_gene_freq_by_uniq_VDJ_IGHG3..IGHV3.11.1" "75"
"V_gene_freq_by_cluster_IGHE..IGHV1.69.2" "75"
"V_gene_freq_by_cluster_IGHE..IGHV3.72" "75"
"V_gene_freq_by_cluster_IGHE..IGHV3.73" "75"
"Mean_cluster_size_IGHE..IGHV3.9" "75"

"Mean_cluster_size_IGHG3..IGHV3.11" "75"
"V_gene_freq_by_uniq_VDJ_expanded..IGHV3.9" "76"
"V_gene_freq_by_uniq_VDJ_IGHD.IGHM_mutated..IGHV3.9" "76"
"V_gene_freq_by_uniq_VDJ_IGHM..IGHV3.9" "76"
"V_gene_freq_by_uniq_VDJ_unexpanded..IGHV3.9" "76"
"V_gene_freq_by_cluster_expanded..IGHV3.9" "76"
"V_gene_freq_by_cluster_IGHD..IGHV3.9" "76"
"V_gene_freq_by_cluster_IGHD.IGHM_mutated..IGHV3.9" "76"
"V_gene_freq_by_cluster_IGHM..IGHV3.9" "76"
"V_gene_freq_by_cluster_unexpanded..IGHV3.9" "76"
"V_gene_freq_by_uniq_VDJ_expanded..IGHV3.21" "77"
"V_gene_freq_by_uniq_VDJ_IGHD.IGHM_mutated..IGHV3.21" "77"
"V_gene_freq_by_uniq_VDJ_IGHM..IGHV3.21" "77"
"V_gene_freq_by_uniq_VDJ_unexpanded..IGHV3.21" "77"
"V_gene_freq_by_cluster_expanded..IGHV3.21" "77"
"V_gene_freq_by_cluster_IGHD..IGHV3.21" "77"
"V_gene_freq_by_cluster_IGHD.IGHM_mutated..IGHV3.21" "77"
"V_gene_freq_by_cluster_IGHM..IGHV3.21" "77"
"V_gene_freq_by_cluster_unexpanded..IGHV3.21" "77"
"Mean_cluster_size_Class_switched..IGHV3.64" "78"
"Mean_cluster_size_expanded..IGHV3.49" "78"
"Mean_cluster_size_expanded..IGHV3.64" "78"
"Mean_cluster_size_IGHD.IGHM_mutated..IGHV3.21" "78"
"Mean_cluster_size_IGHG2..IGHV3.21" "78"
"Mean_cluster_size_IGHM..IGHV3.49" "78"
"Mean_cluster_size_IGHM..IGHV3.64" "78"
"Mean_cluster_size_IGHM..IGHV4.34" "78"
"Mean_cluster_size_IGHM..IGHV4.4" "78"
"Mean_cluster_size_Class_switched..IGHV3.21" "79"
"Mean_cluster_size_Class_switched..IGHV3.48" "79"
"Mean_cluster_size_expanded..IGHV3.21" "79"
"Mean_cluster_size_expanded..IGHV3.48" "79"
"Mean_cluster_size_IGHA1..IGHV3.21" "79"
"Mean_cluster_size_IGHA1..IGHV3.48" "79"
"Mean_cluster_size_IGHD.IGHM_mutated..IGHV5.51" "79"
"Mean_cluster_size_IGHG2..IGHV3.48" "79"
"Mean_cluster_size_IGHM..IGHV3.48" "79"
"V_gene_freq_by_uniq_VDJ_expanded..IGHV4.39" "80"
"V_gene_freq_by_uniq_VDJ_IGHD.IGHM_mutated..IGHV4.39" "80"
"V_gene_freq_by_uniq_VDJ_IGHM..IGHV4.39" "80"
"V_gene_freq_by_uniq_VDJ_unexpanded..IGHV4.39" "80"
"V_gene_freq_by_cluster_expanded..IGHV4.39" "80"
"V_gene_freq_by_cluster_IGHD..IGHV4.39" "80"
"V_gene_freq_by_cluster_IGHD.IGHM_mutated..IGHV4.39" "80"
"V_gene_freq_by_cluster_IGHM..IGHV4.39" "80"
"V_gene_freq_by_cluster_unexpanded..IGHV4.39" "80"
"V_gene_freq_by_uniq_VDJ_Class_switched..IGHV6.1" "81"
"V_gene_freq_by_uniq_VDJ_expanded..IGHV6.1" "81"
"V_gene_freq_by_uniq_VDJ_IGHA1..IGHV6.1" "81"

"V_gene_freq_by_uniq_VDJ_IGHG3..IGHV6.1" "81"
"V_gene_freq_by_uniq_VDJ_unexpanded..IGHV6.1" "81"
"V_gene_freq_by_cluster_Class_switched..IGHV6.1" "81"
"V_gene_freq_by_cluster_IGHA1..IGHV6.1" "81"
"V_gene_freq_by_cluster_IGHG3..IGHV6.1" "81"
"V_gene_freq_by_cluster_unexpanded..IGHV6.1" "81"
"V4_34_AVY_unmut..IGHA1" "82"
"V4_34_AVY_unmut..IGHG2" "82"
"V4_34_NHS_unmut..IGHA1" "82"
"V4_34_NHS_unmut..IGHG2" "82"
"V4_34_AVY_NHS_unmut..IGHA1" "82"
"V4_34_AVY_NHS_unmut..IGHG2" "82"
"V4_34_AVY_NHS_unmut..class_switched" "82"
"V_gene_freq_by_uniq_VDJ_IGHA1..IGHV4.34" "82"
"V_gene_freq_by_cluster_IGHA1..IGHV4.34" "82"
"V_gene_freq_by_uniq_VDJ_IGHG3..IGHV3.49.1" "83"
"V_gene_freq_by_uniq_VDJ_IGHG3..IGHV3.64.1" "83"
"V_gene_freq_by_uniq_VDJ_IGHG3..IGHV4.4.1" "83"
"V_gene_freq_by_uniq_VDJ_IGHG3..IGHV4.55" "83"
"V_gene_freq_by_cluster_IGHG3..IGHV3.49.1" "83"
"V_gene_freq_by_cluster_IGHG3..IGHV4.55" "83"
"Mean_cluster_size_IGHG3..IGHV3.49" "83"
"Mean_cluster_size_IGHG3..IGHV3.64" "83"
"Mean_cluster_size_IGHG3..IGHV4.4" "83"
"V_gene_freq_by_uniq_VDJ_IGHD.IGHM_mutated..IGHV3.49" "84"
"V_gene_freq_by_uniq_VDJ_unexpanded..IGHV3.49" "84"
"V_gene_freq_by_cluster_Class_switched..IGHV3.49" "84"
"V_gene_freq_by_cluster_expanded..IGHV3.49" "84"
"V_gene_freq_by_cluster_IGHA1..IGHV3.49" "84"
"V_gene_freq_by_cluster_IGHD.IGHM_mutated..IGHV3.49" "84"
"V_gene_freq_by_cluster_IGHG2..IGHV3.49" "84"
"V_gene_freq_by_cluster_IGHM..IGHV3.49" "84"
"V_gene_freq_by_cluster_unexpanded..IGHV3.49" "84"
"Percentage_unique_BCRs_in_switched..IGHG3" "85"
"Percentage_unmutated..IGHG3" "85"
"V_gene_freq_by_uniq_VDJ_Class_switched..IGHV1.18" "85"
"V_gene_freq_by_uniq_VDJ_IGHG3..IGHV1.18" "85"
"J_gene_freq_by_uniq_VDJ_IGHE..IGHJ6.1" "85"
"V_gene_freq_by_uniq_VDJ_IGHE..IGHV3.53" "85"
"V_gene_freq_by_uniq_VDJ_IGHG3..IGHV3.53.1" "85"
"Mean_cluster_size_IGHE..IGHV3.53" "85"
"Mean_cluster_size_IGHG3..IGHV3.53" "85"
"Percentage_unique_BCRs_per_isotype_group..IGHA1" "86"
"Percentage_unique_BCRs_per_isotype_group..IGHA2" "86"
"Percentage_unique_BCRs_in_switched..IGHA1" "86"
"Percentage_unique_BCRs_in_switched..IGHA2" "86"
"Relative_class_switching_absolute..IGHA1.01.IGHG2.03" "86"
"Relative_class_switching_normalised..IGHA1.IGHD.M" "86"
"Relative_class_switching_normalised..IGHA1.IGHG2" "86"

"Relative_class_switching_normalised..IGHD.M.IGHG2" "86"
"Mean_cluster_size_IGHD..IGHV3.74" "86"
"V_gene_freq_by_uniq_VDJ_IGHG3..IGHV1.69" "87"
"V_gene_freq_by_cluster_IGHG3..IGHV1.69" "87"
"V4_34_NHS_unmut..IGHG3.1" "87"
"V4_34_AVY_NHS_unmut..IGHG3.1" "87"
"V_gene_freq_by_uniq_VDJ_IGHG3..IGHV3.NL1.1" "87"
"V_gene_freq_by_uniq_VDJ_IGHG3..IGHV4.28.1" "87"
"V_gene_freq_by_cluster_IGHG3..IGHV3.NL1.1" "87"
"V_gene_freq_by_cluster_IGHG3..IGHV4.28.1" "87"
"Mean_cluster_size_IGHG3..IGHV3.NL1" "87"
"V_gene_freq_by_uniq_VDJ_IGHD..IGHV4.31" "88"
"V_gene_freq_by_uniq_VDJ_IGHD.IGHM_mutated..IGHV4.31" "88"
"V_gene_freq_by_uniq_VDJ_IGHM..IGHV4.31" "88"
"V_gene_freq_by_uniq_VDJ_unexpanded..IGHV4.31" "88"
"V_gene_freq_by_cluster_IGHD..IGHV4.31" "88"
"V_gene_freq_by_cluster_IGHD.IGHM_mutated..IGHV4.31" "88"
"V_gene_freq_by_cluster_IGHM..IGHV4.31" "88"
"V_gene_freq_by_cluster_unexpanded..IGHV4.31" "88"
"V_gene_freq_by_uniq_VDJ_Class_switched..IGHV3.30.3" "89"
"V_gene_freq_by_uniq_VDJ_IGHA1..IGHV3.30.3" "89"
"V_gene_freq_by_uniq_VDJ_IGHG2..IGHV3.30.3" "89"
"V_gene_freq_by_uniq_VDJ_IGHG3..IGHV3.30.3" "89"
"V_gene_freq_by_cluster_Class_switched..IGHV3.30.3" "89"
"V_gene_freq_by_cluster_IGHA1..IGHV3.30.3" "89"
"V_gene_freq_by_cluster_IGHG2..IGHV3.30.3" "89"
"V_gene_freq_by_cluster_IGHG3..IGHV3.30.3" "89"
"V_gene_freq_by_uniq_VDJ_expanded..IGHV1.24" "90"
"V_gene_freq_by_uniq_VDJ_IGHD..IGHV1.24" "90"
"V_gene_freq_by_uniq_VDJ_IGHD.IGHM_mutated..IGHV1.24" "90"
"V_gene_freq_by_uniq_VDJ_IGHM..IGHV1.24" "90"
"V_gene_freq_by_uniq_VDJ_unexpanded..IGHV1.24" "90"
"V_gene_freq_by_cluster_IGHD..IGHV1.24" "90"
"V_gene_freq_by_cluster_IGHD.IGHM_mutated..IGHV1.24" "90"
"V_gene_freq_by_cluster_unexpanded..IGHV1.24" "90"
"V_gene_freq_by_uniq_VDJ_expanded..IGHV2.70" "91"
"V_gene_freq_by_uniq_VDJ_IGHD.IGHM_mutated..IGHV2.70" "91"
"V_gene_freq_by_uniq_VDJ_IGHM..IGHV2.70" "91"
"V_gene_freq_by_uniq_VDJ_unexpanded..IGHV2.70" "91"
"V_gene_freq_by_cluster_expanded..IGHV2.70" "91"
"V_gene_freq_by_cluster_IGHD.IGHM_mutated..IGHV2.70" "91"
"V_gene_freq_by_cluster_IGHM..IGHV2.70" "91"
"V_gene_freq_by_cluster_unexpanded..IGHV2.70" "91"
"Vertex_Gini_Index..IGHG3.1" "92"
"Cluster_Gini_Index..IGHG3.1" "92"
"mean_vertex_size..IGHG3.1" "92"
"Percentage_max_cluster_size..IGHG3.1" "92"
"Percentage_max_vertex_size..IGHG3.1" "92"
"D5..IGHG3.18.1" "92"

"V_gene_freq_by_uniq_VDJ_IGHG3..IGHV3.66.1" "92"
"Mean_cluster_size_IGHG3..IGHV3.66" "92"
"V_gene_freq_by_uniq_VDJ_Class_switched..IGHV4.28" "93"
"V_gene_freq_by_uniq_VDJ_IGHA1..IGHV4.28" "93"
"V_gene_freq_by_uniq_VDJ_IGHG2..IGHV4.28" "93"
"V_gene_freq_by_uniq_VDJ_IGHG3..IGHV4.28" "93"
"V_gene_freq_by_cluster_Class_switched..IGHV4.28" "93"
"V_gene_freq_by_cluster_IGHA1..IGHV4.28" "93"
"V_gene_freq_by_cluster_IGHG2..IGHV4.28" "93"
"V_gene_freq_by_cluster_IGHG3..IGHV4.28" "93"
"V_gene_freq_by_uniq_VDJ_IGHD..IGHV1.58" "94"
"V_gene_freq_by_uniq_VDJ_IGHD.IGHM_mutated..IGHV1.58" "94"
"V_gene_freq_by_uniq_VDJ_IGHM..IGHV1.58" "94"
"V_gene_freq_by_uniq_VDJ_unexpanded..IGHV1.58" "94"
"V_gene_freq_by_cluster_IGHD..IGHV1.58" "94"
"V_gene_freq_by_cluster_IGHD.IGHM_mutated..IGHV1.58" "94"
"V_gene_freq_by_cluster_IGHM..IGHV1.58" "94"
"V_gene_freq_by_cluster_unexpanded..IGHV1.58" "94"
"V_gene_freq_by_uniq_VDJ_Class_switched..IGHV1.58" "95"
"V_gene_freq_by_uniq_VDJ_expanded..IGHV1.58" "95"
"V_gene_freq_by_uniq_VDJ_IGHG2..IGHV1.58" "95"
"V_gene_freq_by_uniq_VDJ_IGHG3..IGHV1.58" "95"
"V_gene_freq_by_cluster_Class_switched..IGHV1.58" "95"
"V_gene_freq_by_cluster_expanded..IGHV1.58" "95"
"V_gene_freq_by_cluster_IGHG2..IGHV1.58" "95"
"V_gene_freq_by_cluster_IGHG3..IGHV1.58" "95"
"V_gene_freq_by_uniq_VDJ_Class_switched..IGHV4.38.2" "96"
"V_gene_freq_by_uniq_VDJ_IGHA1..IGHV4.38.2" "96"
"V_gene_freq_by_uniq_VDJ_IGHG2..IGHV4.38.2" "96"
"V_gene_freq_by_uniq_VDJ_IGHG3..IGHV4.38.2" "96"
"V_gene_freq_by_cluster_Class_switched..IGHV4.38.2" "96"
"V_gene_freq_by_cluster_IGHA1..IGHV4.38.2" "96"
"V_gene_freq_by_cluster_IGHG2..IGHV4.38.2" "96"
"V_gene_freq_by_cluster_IGHG3..IGHV4.38.2" "96"
"V_gene_freq_by_uniq_VDJ_Class_switched..IGHV3.33" "97"
"V_gene_freq_by_uniq_VDJ_IGHA1..IGHV3.33" "97"
"V_gene_freq_by_uniq_VDJ_IGHG2..IGHV3.33" "97"
"V_gene_freq_by_uniq_VDJ_IGHG3..IGHV3.33" "97"
"V_gene_freq_by_cluster_Class_switched..IGHV3.33" "97"
"V_gene_freq_by_cluster_IGHA1..IGHV3.33" "97"
"V_gene_freq_by_cluster_IGHG2..IGHV3.33" "97"
"V_gene_freq_by_cluster_IGHG3..IGHV3.33" "97"
"J_gene_freq_by_uniq_VDJ_Class_switched..IGHJ3" "98"
"J_gene_freq_by_uniq_VDJ_expanded..IGHJ3" "98"
"J_gene_freq_by_uniq_VDJ_IGHA1..IGHJ3" "98"
"J_gene_freq_by_uniq_VDJ_unexpanded..IGHJ3" "98"
"V_gene_freq_by_uniq_VDJ_IGHE..IGHV3.30.3" "98"
"V_gene_freq_by_uniq_VDJ_IGHG3..IGHV3.48.1" "98"
"Mean_cluster_size_IGHE..IGHV3.30.3" "98"

"Mean_cluster_size_IGHG3..IGHV3.48.1" "98"
"V_gene_freq_by_uniq_VDJ_IGHD.IGHM_mutated..IGHV4.4" "99"
"V_gene_freq_by_uniq_VDJ_IGHM..IGHV4.4" "99"
"V_gene_freq_by_cluster_IGHD..IGHV4.4" "99"
"V_gene_freq_by_cluster_IGHD.IGHM_mutated..IGHV4.4" "99"
"V_gene_freq_by_cluster_IGHM..IGHV4.4" "99"
"V_gene_freq_by_uniq_VDJ_IGHE..IGHV4.30.2" "99"
"V_gene_freq_by_uniq_VDJ_IGHE..IGHV4.4" "99"
"Mean_cluster_size_IGHE..IGHV4.4" "99"
"Mean_cluster_size_Class_switched..IGHV3.9" "100"
"Mean_cluster_size_expanded..IGHV3.9" "100"
"Mean_cluster_size_expanded..IGHV5.51" "100"
"Mean_cluster_size_IGHA1..IGHV3.9" "100"
"Mean_cluster_size_IGHG3..IGHV3.48" "100"
"Mean_cluster_size_IGHM..IGHV3.23" "100"
"Mean_cluster_size_IGHM..IGHV3.9" "100"
"Mean_cluster_size_IGHM..IGHV5.51" "100"
"V_gene_freq_by_uniq_VDJ_IGHE..IGHV1.24" "101"
"V_gene_freq_by_uniq_VDJ_IGHE..IGHV4.59" "101"
"V_gene_freq_by_uniq_VDJ_IGHG3..IGHV4.30.4.1" "101"
"V_gene_freq_by_cluster_IGHE..IGHV1.24" "101"
"V_gene_freq_by_cluster_IGHE..IGHV4.30.4" "101"
"Mean_cluster_size_IGHE..IGHV1.24" "101"
"Mean_cluster_size_IGHE..IGHV4.59" "101"
"Mean_cluster_size_IGHG3..IGHV4.30.4" "101"
"V_gene_freq_by_uniq_VDJ_IGHA1..IGHV1.18" "102"
"V_gene_freq_by_uniq_VDJ_IGHA1..IGHV5.51" "102"
"V_gene_freq_by_uniq_VDJ_IGHG2..IGHV1.18" "102"
"V_gene_freq_by_cluster_Class_switched..IGHV1.18" "102"
"V_gene_freq_by_cluster_Class_switched..IGHV5.51" "102"
"V_gene_freq_by_cluster_IGHA1..IGHV1.18" "102"
"V_gene_freq_by_cluster_IGHG2..IGHV1.18" "102"
"V_gene_freq_by_cluster_IGHG2..IGHV5.51" "102"
"V_gene_freq_by_uniq_VDJ_IGHG3..IGHV4.34.1" "103"
"V_gene_freq_by_uniq_VDJ_IGHG3..IGHV4.59.1" "103"
"V_gene_freq_by_cluster_IGHG3..IGHV4.34.1" "103"
"V_gene_freq_by_cluster_IGHG3..IGHV4.39.1" "103"
"V_gene_freq_by_cluster_IGHG3..IGHV4.4.1" "103"
"V_gene_freq_by_cluster_IGHG3..IGHV4.59.1" "103"
"V_gene_freq_by_cluster_IGHG3..IGHV4.61.1" "103"
"Mean_cluster_size_IGHG3..IGHV4.59" "103"
"J_gene_freq_by_uniq_VDJ_Class_switched..IGHJ5" "104"
"J_gene_freq_by_uniq_VDJ_expanded..IGHJ5" "104"
"J_gene_freq_by_uniq_VDJ_IGHA1..IGHJ5" "104"
"J_gene_freq_by_uniq_VDJ_IGHE..IGHJ5" "104"
"J_gene_freq_by_uniq_VDJ_IGHG2..IGHJ5" "104"
"J_gene_freq_by_uniq_VDJ_IGHG3..IGHJ5" "104"
"J_gene_freq_by_uniq_VDJ_IGHM..IGHJ5" "104"
"J_gene_freq_by_uniq_VDJ_unexpanded..IGHJ5" "104"

"V_gene_freq_by_uniq_VDJ_IGHD.IGHM_mutated..IGHV3.13" "105"
"V_gene_freq_by_uniq_VDJ_unexpanded..IGHV4.28" "105"
"V_gene_freq_by_cluster_IGHD.IGHM_mutated..IGHV3.13" "105"
"V_gene_freq_by_cluster_unexpanded..IGHV4.28" "105"
"V_gene_freq_by_uniq_VDJ_IGHG3..IGHV3.71" "105"
"V_gene_freq_by_uniq_VDJ_IGHG3..IGHV4.30.2.1" "105"
"V_gene_freq_by_cluster_IGHG3..IGHV3.71" "105"
"Mean_cluster_size_IGHG3..IGHV4.30.2" "105"
"D5..IGHG3.17" "106"
"D10..IGHG3.17" "106"
"V_gene_freq_by_uniq_VDJ_IGHG3..IGHV2.70D.1" "106"
"V_gene_freq_by_uniq_VDJ_IGHG3..IGHV3.73.1" "106"
"V_gene_freq_by_cluster_IGHG3..IGHV2.70D.1" "106"
"V_gene_freq_by_cluster_IGHG3..IGHV3.73.1" "106"
"Mean_cluster_size_IGHG3..IGHV2.70D" "106"
"Mean_cluster_size_IGHG3..IGHV3.73" "106"
"V_gene_freq_by_uniq_VDJ_Class_switched..IGHV3.43D" "107"
"V_gene_freq_by_uniq_VDJ_IGHA1..IGHV3.43D" "107"
"V_gene_freq_by_uniq_VDJ_IGHG2..IGHV3.43D" "107"
"V_gene_freq_by_uniq_VDJ_IGHG3..IGHV3.43D" "107"
"V_gene_freq_by_cluster_Class_switched..IGHV3.43D" "107"
"V_gene_freq_by_cluster_IGHA1..IGHV3.43D" "107"
"V_gene_freq_by_cluster_IGHG2..IGHV3.43D" "107"
"V_gene_freq_by_cluster_IGHG3..IGHV3.43D" "107"
"V_gene_freq_by_uniq_VDJ_Class_switched..IGHV1.2" "108"
"V_gene_freq_by_uniq_VDJ_IGHA1..IGHV1.2" "108"
"V_gene_freq_by_uniq_VDJ_IGHG2..IGHV1.2" "108"
"V_gene_freq_by_uniq_VDJ_IGHG3..IGHV1.2" "108"
"V_gene_freq_by_cluster_Class_switched..IGHV1.2" "108"
"V_gene_freq_by_cluster_IGHA1..IGHV1.2" "108"
"V_gene_freq_by_cluster_IGHG2..IGHV1.2" "108"
"V_gene_freq_by_cluster_IGHG3..IGHV1.2" "108"
"Relative_class_switching_absolute..IGHE.02.IGHG2.03.1" "109"
"Relative_class_switching_normalised..IGHE.IGHG2.1" "109"
"V_gene_freq_by_uniq_VDJ_IGHE..IGHV3.33" "109"
"V_gene_freq_by_uniq_VDJ_IGHE..IGHV3.43" "109"
"V_gene_freq_by_cluster_IGHE..IGHV3.13" "109"
"V_gene_freq_by_cluster_IGHE..IGHV3.30.3" "109"
"V_gene_freq_by_cluster_IGHE..IGHV3.43" "109"
"Mean_cluster_size_IGHE..IGHV3.33" "109"
"V_gene_freq_by_uniq_VDJ_expanded..IGHV4.59" "110"
"V_gene_freq_by_uniq_VDJ_IGHM..IGHV4.59" "110"
"V_gene_freq_by_uniq_VDJ_unexpanded..IGHV4.59" "110"
"V_gene_freq_by_cluster_expanded..IGHV4.59" "110"
"V_gene_freq_by_cluster_IGHD..IGHV4.59" "110"
"V_gene_freq_by_cluster_IGHM..IGHV4.59" "110"
"V_gene_freq_by_cluster_unexpanded..IGHV4.59" "110"
"V_gene_freq_by_uniq_VDJ_IGHE..IGHV3.72" "111"
"V_gene_freq_by_uniq_VDJ_IGHE..IGHV4.34" "111"

"V_gene_freq_by_uniq_VDJ_IGHE..IGHV4.55" "111"
"V_gene_freq_by_cluster_IGHE..IGHV4.55" "111"
"Mean_cluster_size_IGHE..IGHV3.72" "111"
"Mean_cluster_size_IGHE..IGHV4.34" "111"
"Mean_cluster_size_IGHG3..IGHV4.34" "111"
"V_gene_freq_by_uniq_VDJ_Class_switched..IGHV3.49" "112"
"V_gene_freq_by_uniq_VDJ_expanded..IGHV3.49" "112"
"V_gene_freq_by_uniq_VDJ_IGHA1..IGHV3.49" "112"
"V_gene_freq_by_uniq_VDJ_IGHD..IGHV3.49" "112"
"V_gene_freq_by_uniq_VDJ_IGHG2..IGHV3.49" "112"
"V_gene_freq_by_uniq_VDJ_IGHG3..IGHV3.49" "112"
"V_gene_freq_by_uniq_VDJ_IGHM..IGHV3.49" "112"
"Vertex_Reyni..all" "113"
"Vertex_Reyni..IGHD" "113"
"Vertex_Reyni..IGHM" "113"
"Cluster_Reyni..all" "113"
"Cluster_Reyni..IGHD" "113"
"Cluster_Reyni..IGHM" "113"
"J_gene_freq_by_uniq_VDJ_IGHD.IGHM_unmutated..IGHJ3" "113"
"Vertex_Reyni..IGHG3" "114"
"Cluster_Reyni..IGHG3" "114"
"Mean_CDR3_lengths..IGHG3" "114"
"mean_CDR_FWR_ratio..IGHG3" "114"
"mean_CDR3_charge..IGHG3" "114"
"V_gene_freq_by_cluster_IGHG3..IGHV3.11" "114"
"V_gene_freq_by_cluster_IGHG3..IGHV3.48" "114"
"V_gene_freq_by_uniq_VDJ_Class_switched..IGHV4.30.2" "115"
"V_gene_freq_by_uniq_VDJ_expanded..IGHV4.30.2" "115"
"V_gene_freq_by_uniq_VDJ_IGHG2..IGHV4.30.2" "115"
"V_gene_freq_by_uniq_VDJ_unexpanded..IGHV4.30.2" "115"
"V_gene_freq_by_cluster_Class_switched..IGHV4.30.2" "115"
"V_gene_freq_by_cluster_IGHG2..IGHV4.30.2" "115"
"V_gene_freq_by_cluster_unexpanded..IGHV4.30.2" "115"
"V_gene_freq_by_uniq_VDJ_Class_switched..IGHV2.70" "116"
"V_gene_freq_by_uniq_VDJ_IGHA1..IGHV2.70" "116"
"V_gene_freq_by_uniq_VDJ_IGHG2..IGHV2.70" "116"
"V_gene_freq_by_uniq_VDJ_IGHG3..IGHV2.70" "116"
"V_gene_freq_by_cluster_Class_switched..IGHV2.70" "116"
"V_gene_freq_by_cluster_IGHA1..IGHV2.70" "116"
"V_gene_freq_by_cluster_IGHG2..IGHV2.70" "116"
"Vertex_Gini_Index..IGHG3" "117"
"Cluster_Gini_Index..IGHG3" "117"
"mean_vertex_size..IGHG3" "117"
"Percentage_max_cluster_size..IGHG3" "117"
"Percentage_max_vertex_size..IGHG3" "117"
"D5..IGHG3.18" "117"
"D10..IGHG3.18" "117"
"V_gene_freq_by_uniq_VDJ_expanded..IGHV3.71" "118"
"V_gene_freq_by_uniq_VDJ_IGHA1..IGHV3.71" "118"

"V_gene_freq_by_uniq_VDJ_IGHD.IGHM_mutated..IGHV3.71" "118"
"V_gene_freq_by_cluster_IGHA1..IGHV3.71" "118"
"V_gene_freq_by_cluster_IGHD.IGHM_mutated..IGHV3.71" "118"
"V_gene_freq_by_uniq_VDJ_IGHG3..IGHV3.30.1" "118"
"Mean_cluster_size_IGHG3..IGHV3.30.1" "118"
"V_gene_freq_by_uniq_VDJ_Class_switched..IGHV1.69" "119"
"V_gene_freq_by_uniq_VDJ_IGHA1..IGHV1.69" "119"
"V_gene_freq_by_uniq_VDJ_IGHG2..IGHV1.69" "119"
"V_gene_freq_by_cluster_Class_switched..IGHV1.69" "119"
"V_gene_freq_by_cluster_expanded..IGHV1.69" "119"
"V_gene_freq_by_cluster_IGHA1..IGHV1.69" "119"
"V_gene_freq_by_cluster_IGHG2..IGHV1.69" "119"
"V_gene_freq_by_uniq_VDJ_Class_switched..IGHV3.11" "120"
"V_gene_freq_by_uniq_VDJ_IGHA1..IGHV3.11" "120"
"V_gene_freq_by_uniq_VDJ_IGHD..IGHV3.11" "120"
"V_gene_freq_by_uniq_VDJ_IGHG2..IGHV3.11" "120"
"V_gene_freq_by_cluster_Class_switched..IGHV3.11" "120"
"V_gene_freq_by_cluster_IGHA1..IGHV3.11" "120"
"V_gene_freq_by_cluster_IGHG2..IGHV3.11" "120"
"V_gene_freq_by_uniq_VDJ_Class_switched..IGHV7.4.1" "121"
"V_gene_freq_by_uniq_VDJ_IGHA1..IGHV7.4.1" "121"
"V_gene_freq_by_uniq_VDJ_IGHG2..IGHV7.4.1" "121"
"V_gene_freq_by_cluster_Class_switched..IGHV7.4.1" "121"
"V_gene_freq_by_cluster_expanded..IGHV7.4.1" "121"
"V_gene_freq_by_cluster_IGHA1..IGHV7.4.1" "121"
"V_gene_freq_by_cluster_IGHG2..IGHV7.4.1" "121"
"V_gene_freq_by_uniq_VDJ_IGHD.IGHM_mutated..IGHV3.15" "122"
"V_gene_freq_by_uniq_VDJ_unexpanded..IGHV3.15" "122"
"V_gene_freq_by_cluster_expanded..IGHV3.15" "122"
"V_gene_freq_by_cluster_IGHD..IGHV3.15" "122"
"V_gene_freq_by_cluster_IGHD.IGHM_mutated..IGHV3.15" "122"
"V_gene_freq_by_cluster_IGHM..IGHV3.15" "122"
"V_gene_freq_by_cluster_unexpanded..IGHV3.15" "122"
"V_gene_freq_by_uniq_VDJ_Class_switched..IGHV1.8" "123"
"V_gene_freq_by_uniq_VDJ_IGHG2..IGHV1.8" "123"
"V_gene_freq_by_uniq_VDJ_IGHG3..IGHV1.8" "123"
"V_gene_freq_by_cluster_Class_switched..IGHV1.8" "123"
"V_gene_freq_by_cluster_IGHA1..IGHV1.8" "123"
"V_gene_freq_by_cluster_IGHG2..IGHV1.8" "123"
"V_gene_freq_by_cluster_IGHG3..IGHV1.8" "123"
"V_gene_freq_by_uniq_VDJ_IGHA1..IGHV1.8" "124"
"V_gene_freq_by_uniq_VDJ_IGHE..IGHV3.66" "124"
"V_gene_freq_by_uniq_VDJ_IGHE..IGHV3.NL1" "124"
"V_gene_freq_by_cluster_IGHE..IGHV1.58" "124"
"V_gene_freq_by_cluster_IGHE..IGHV3.69.1" "124"
"V_gene_freq_by_cluster_IGHE..IGHV3.NL1" "124"
"Mean_cluster_size_IGHE..IGHV3.66" "124"
"J_gene_freq_by_uniq_VDJ_Class_switched..IGHJ1" "125"
"J_gene_freq_by_uniq_VDJ_IGHA1..IGHJ1" "125"

"V_gene_freq_by_uniq_VDJ_IGHE..IGHV3.49" "125"
"V_gene_freq_by_uniq_VDJ_IGHG3..IGHV1.3.1" "125"
"V_gene_freq_by_cluster_IGHG3..IGHV1.3.1" "125"
"Mean_cluster_size_IGHE..IGHV3.49" "125"
"Mean_cluster_size_IGHG3..IGHV1.3" "125"
"V_gene_freq_by_uniq_VDJ_Class_switched..IGHV3.23" "126"
"V_gene_freq_by_uniq_VDJ_IGHA1..IGHV3.23" "126"
"V_gene_freq_by_uniq_VDJ_IGHG2..IGHV3.23" "126"
"V_gene_freq_by_uniq_VDJ_IGHG3..IGHV3.23" "126"
"V_gene_freq_by_cluster_Class_switched..IGHV3.23" "126"
"V_gene_freq_by_cluster_IGHA1..IGHV3.23" "126"
"V_gene_freq_by_cluster_IGHG2..IGHV3.23" "126"
"V_gene_freq_by_uniq_VDJ_Class_switched..IGHV3.21" "127"
"V_gene_freq_by_uniq_VDJ_IGHA1..IGHV3.21" "127"
"V_gene_freq_by_uniq_VDJ_IGHG2..IGHV3.21" "127"
"V_gene_freq_by_cluster_Class_switched..IGHV3.21" "127"
"V_gene_freq_by_cluster_IGHA1..IGHV3.21" "127"
"V_gene_freq_by_cluster_IGHG2..IGHV3.21" "127"
"V_gene_freq_by_cluster_IGHG3..IGHV3.21" "127"
"V_gene_freq_by_uniq_VDJ_IGHD.IGHM_mutated..IGHV4.59" "128"
"V_gene_freq_by_cluster_IGHD.IGHM_mutated..IGHV4.59" "128"
"Mean_cluster_size_expanded..IGHV3.30" "128"
"Mean_cluster_size_IGHG3..IGHV3.30" "128"
"V_gene_freq_by_uniq_VDJ_IGHG3..IGHV1.NL1" "128"
"V_gene_freq_by_cluster_IGHG3..IGHV1.NL1" "128"
"V_gene_freq_by_uniq_VDJ_IGHD.IGHM_mutated..IGHV5.10.1" "129"
"V_gene_freq_by_uniq_VDJ_IGHM..IGHV5.10.1" "129"
"V_gene_freq_by_uniq_VDJ_unexpanded..IGHV5.10.1" "129"
"V_gene_freq_by_cluster_IGHD.IGHM_mutated..IGHV5.10.1" "129"
"V_gene_freq_by_cluster_IGHM..IGHV5.10.1" "129"
"V_gene_freq_by_cluster_unexpanded..IGHV5.10.1" "129"
"V_gene_freq_by_uniq_VDJ_Class_switched..IGHV1.3" "130"
"V_gene_freq_by_uniq_VDJ_IGHG2..IGHV1.3" "130"
"V_gene_freq_by_uniq_VDJ_unexpanded..IGHV1.3" "130"
"V_gene_freq_by_cluster_Class_switched..IGHV1.3" "130"
"V_gene_freq_by_cluster_IGHA1..IGHV1.3" "130"
"V_gene_freq_by_cluster_unexpanded..IGHV1.3" "130"
"V_gene_freq_by_uniq_VDJ_IGHD.IGHM_mutated..IGHV7.4.1" "131"
"V_gene_freq_by_uniq_VDJ_IGHM..IGHV7.4.1" "131"
"V_gene_freq_by_uniq_VDJ_unexpanded..IGHV7.4.1" "131"
"V_gene_freq_by_cluster_IGHD.IGHM_mutated..IGHV7.4.1" "131"
"V_gene_freq_by_cluster_IGHM..IGHV7.4.1" "131"
"V_gene_freq_by_cluster_unexpanded..IGHV7.4.1" "131"
"V_gene_freq_by_uniq_VDJ_Class_switched..IGHV4.34" "132"
"V_gene_freq_by_uniq_VDJ_IGHG2..IGHV4.34" "132"
"V_gene_freq_by_cluster_Class_switched..IGHV4.34" "132"
"V_gene_freq_by_cluster_IGHG2..IGHV4.34" "132"
"V_gene_freq_by_uniq_VDJ_IGHE..IGHV1.8" "132"
"Mean_cluster_size_IGHE..IGHV1.8" "132"

"mean_CDR3_charge..IGHM" "133"
"V_gene_freq_by_uniq_VDJ_IGHD.IGHM_mutated..IGHV3.74" "133"
"V_gene_freq_by_cluster_IGHD.IGHM_mutated..IGHV3.74" "133"
"V_gene_freq_by_uniq_VDJ_IGHG3..IGHV3.64D.1" "133"
"V_gene_freq_by_cluster_IGHG3..IGHV3.64D.1" "133"
"Mean_cluster_size_IGHG3..IGHV3.64D" "133"
"J_gene_freq_by_uniq_VDJ_Class_switched..IGHJ2" "134"
"J_gene_freq_by_uniq_VDJ_expanded..IGHJ2" "134"
"J_gene_freq_by_uniq_VDJ_IGHA1..IGHJ2" "134"
"J_gene_freq_by_uniq_VDJ_IGHG2..IGHJ2" "134"
"J_gene_freq_by_uniq_VDJ_IGHM..IGHJ2" "134"
"J_gene_freq_by_uniq_VDJ_unexpanded..IGHJ2" "134"
"V_gene_freq_by_uniq_VDJ_Class_switched..IGHV3.9" "135"
"V_gene_freq_by_uniq_VDJ_IGHA1..IGHV3.9" "135"
"V_gene_freq_by_uniq_VDJ_IGHG2..IGHV3.9" "135"
"V_gene_freq_by_cluster_Class_switched..IGHV3.9" "135"
"V_gene_freq_by_cluster_IGHA1..IGHV3.9" "135"
"V_gene_freq_by_cluster_IGHG2..IGHV3.9" "135"
"V_gene_freq_by_uniq_VDJ_Class_switched..IGHV1.24" "136"
"V_gene_freq_by_uniq_VDJ_IGHA1..IGHV1.24" "136"
"V_gene_freq_by_uniq_VDJ_IGHG2..IGHV1.24" "136"
"V_gene_freq_by_cluster_Class_switched..IGHV1.24" "136"
"V_gene_freq_by_cluster_IGHA1..IGHV1.24" "136"
"V_gene_freq_by_cluster_IGHG2..IGHV1.24" "136"
"V_gene_freq_by_uniq_VDJ_IGHD.IGHM_mutated..IGHV3.7" "137"
"V_gene_freq_by_cluster_expanded..IGHV3.7" "137"
"V_gene_freq_by_cluster_IGHD..IGHV3.7" "137"
"V_gene_freq_by_cluster_IGHD.IGHM_mutated..IGHV3.7" "137"
"V_gene_freq_by_cluster_IGHG3..IGHV3.7" "137"
"V_gene_freq_by_cluster_IGHM..IGHV3.7" "137"
"V_gene_freq_by_uniq_VDJ_Class_switched..IGHV4.59" "138"
"V_gene_freq_by_uniq_VDJ_IGHA1..IGHV4.59" "138"
"V_gene_freq_by_uniq_VDJ_IGHG2..IGHV4.59" "138"
"V_gene_freq_by_cluster_Class_switched..IGHV4.59" "138"
"V_gene_freq_by_cluster_IGHA1..IGHV4.59" "138"
"V_gene_freq_by_cluster_IGHG2..IGHV4.59" "138"
"V_gene_freq_by_uniq_VDJ_Class_switched..IGHV2.26" "139"
"V_gene_freq_by_uniq_VDJ_IGHA1..IGHV2.26" "139"
"V_gene_freq_by_uniq_VDJ_IGHG2..IGHV2.26" "139"
"V_gene_freq_by_cluster_Class_switched..IGHV2.26" "139"
"V_gene_freq_by_cluster_IGHA1..IGHV2.26" "139"
"V_gene_freq_by_cluster_IGHG2..IGHV2.26" "139"
"V_gene_freq_by_uniq_VDJ_expanded..IGHV4.28" "140"
"V_gene_freq_by_uniq_VDJ_IGHD.IGHM_mutated..IGHV4.28" "140"
"V_gene_freq_by_uniq_VDJ_IGHM..IGHV4.28" "140"
"V_gene_freq_by_cluster_expanded..IGHV4.28" "140"
"V_gene_freq_by_cluster_IGHD.IGHM_mutated..IGHV4.28" "140"
"V_gene_freq_by_cluster_IGHM..IGHV4.28" "140"
"V_gene_freq_by_uniq_VDJ_Class_switched..IGHV4.39" "141"

"V_gene_freq_by_uniq_VDJ_IGHA1..IGHV4.39" "141"
"V_gene_freq_by_uniq_VDJ_IGHG2..IGHV4.39" "141"
"V_gene_freq_by_cluster_Class_switched..IGHV4.39" "141"
"V_gene_freq_by_cluster_IGHA1..IGHV4.39" "141"
"V_gene_freq_by_cluster_IGHG2..IGHV4.39" "141"
"V_gene_freq_by_uniq_VDJ_Class_switched..IGHV1.NL1" "142"
"V_gene_freq_by_uniq_VDJ_IGHA1..IGHV1.58" "142"
"V_gene_freq_by_uniq_VDJ_IGHD.IGHM_mutated..IGHV3.73" "142"
"V_gene_freq_by_cluster_Class_switched..IGHV1.NL1" "142"
"V_gene_freq_by_cluster_IGHA1..IGHV1.58" "142"
"V_gene_freq_by_cluster_IGHD.IGHM_mutated..IGHV3.73" "142"
"Percentage_unique_BCRs_in_switched..IGHG3.1" "143"
"Percentage_unmutated..IGHG3.1" "143"
"V_gene_freq_by_uniq_VDJ_IGHG3..IGHV3.15.1" "143"
"V_gene_freq_by_uniq_VDJ_IGHG3..IGHV3.33.1" "143"
"V_gene_freq_by_cluster_IGHG3..IGHV3.15.1" "143"
"V_gene_freq_by_cluster_IGHG3..IGHV3.33.1" "143"
"V_gene_freq_by_uniq_VDJ_IGHG3..IGHV1.8.1" "144"
"V_gene_freq_by_uniq_VDJ_IGHG3..IGHV3.30.3.1" "144"
"V_gene_freq_by_cluster_IGHG3..IGHV1.8.1" "144"
"V_gene_freq_by_cluster_IGHG3..IGHV3.30.3.1" "144"
"Mean_cluster_size_IGHG3..IGHV1.8" "144"
"Mean_cluster_size_IGHG3..IGHV3.30.3" "144"
"V_gene_freq_by_cluster_expanded..IGHV4.30.2" "145"
"Mean_cluster_size_expanded..IGHV3.53" "145"
"Mean_cluster_size_IGHA1..IGHV3.53" "145"
"Mean_cluster_size_IGHA1..IGHV3.7" "145"
"Mean_cluster_size_IGHG3..IGHV3.7" "145"
"Mean_cluster_size_IGHM..IGHV3.53" "145"
"V_gene_freq_by_uniq_VDJ_expanded..IGHV1.3" "146"
"V_gene_freq_by_uniq_VDJ_IGHA1..IGHV1.3" "146"
"V_gene_freq_by_uniq_VDJ_IGHG3..IGHV1.3" "146"
"V_gene_freq_by_uniq_VDJ_IGHM..IGHV1.3" "146"
"V_gene_freq_by_cluster_expanded..IGHV1.3" "146"
"V_gene_freq_by_cluster_IGHG3..IGHV1.3" "146"
"V_gene_freq_by_uniq_VDJ_unexpanded..IGHV3.71" "147"
"V_gene_freq_by_cluster_unexpanded..IGHV3.71" "147"
"V_gene_freq_by_uniq_VDJ_IGHG3..IGHV3.52" "147"
"V_gene_freq_by_cluster_IGHG3..IGHV3.52" "147"
"Mean_cluster_size_IGHG3..IGHV1.18" "147"
"Mean_cluster_size_IGHG3..IGHV1.58" "147"
"V_gene_freq_by_uniq_VDJ_Class_switched..IGHV3.64D" "148"
"V_gene_freq_by_uniq_VDJ_IGHA1..IGHV3.64D" "148"
"V_gene_freq_by_uniq_VDJ_IGHG2..IGHV3.64D" "148"
"V_gene_freq_by_uniq_VDJ_IGHG3..IGHV3.64D" "148"
"V_gene_freq_by_cluster_IGHG3..IGHV3.64D" "148"
"V_gene_freq_by_uniq_VDJ_IGHE..IGHV6.1" "149"
"V_gene_freq_by_uniq_VDJ_IGHG3..IGHV6.1.1" "149"
"V_gene_freq_by_cluster_IGHG3..IGHV6.1.1" "149"

"Mean_cluster_size_IGHE..IGHV6.1" "149"
"Mean_cluster_size_IGHG3..IGHV6.1" "149"
"J_gene_freq_by_uniq_VDJ_Class_switched..IGHJ6" "150"
"J_gene_freq_by_uniq_VDJ_expanded..IGHJ6" "150"
"J_gene_freq_by_uniq_VDJ_IGHG2..IGHJ6" "150"
"J_gene_freq_by_uniq_VDJ_IGHM..IGHJ6" "150"
"J_gene_freq_by_uniq_VDJ_unexpanded..IGHJ6" "150"
"Percentage_max_cluster_size..IGHD" "151"
"Percentage_max_vertex_size..IGHD" "151"
"Percentage_unique_BCRs_in_switched..IGHG2" "151"
"V_gene_freq_by_uniq_VDJ_IGHM..IGHV3.47" "151"
"V_gene_freq_by_cluster_IGHM..IGHV3.47" "151"
"V_gene_freq_by_uniq_VDJ_IGHD..IGHV1.3" "152"
"V_gene_freq_by_uniq_VDJ_IGHD.IGHM_mutated..IGHV1.3" "152"
"V_gene_freq_by_cluster_IGHD..IGHV1.3" "152"
"V_gene_freq_by_cluster_IGHD.IGHM_mutated..IGHV1.3" "152"
"V_gene_freq_by_cluster_IGHM..IGHV1.3" "152"
"V_gene_freq_by_uniq_VDJ_IGHG2..IGHV3.38.3" "153"
"V_gene_freq_by_uniq_VDJ_IGHM..IGHV3.38.3" "153"
"V_gene_freq_by_cluster_expanded..IGHV3.38.3" "153"
"V_gene_freq_by_cluster_IGHG2..IGHV3.38.3" "153"
"V_gene_freq_by_cluster_IGHM..IGHV3.38.3" "153"
"Mean_cluster_size_Class_switched..IGHV3.11" "154"
"Mean_cluster_size_expanded..IGHV3.11" "154"
"Mean_cluster_size_IGHA1..IGHV3.11" "154"
"Mean_cluster_size_IGHG2..IGHV3.11" "154"
"Mean_cluster_size_IGHM..IGHV3.11" "154"
"V_gene_freq_by_uniq_VDJ_IGHE..IGHV1.18" "155"
"V_gene_freq_by_uniq_VDJ_IGHE..IGHV1.3" "155"
"V_gene_freq_by_uniq_VDJ_IGHG3..IGHV3.72.1" "155"
"Mean_cluster_size_IGHE..IGHV1.18" "155"
"Mean_cluster_size_IGHG3..IGHV3.72" "155"
"V_gene_freq_by_uniq_VDJ_IGHA1..IGHV3.48" "156"
"V_gene_freq_by_uniq_VDJ_IGHG2..IGHV3.48" "156"
"V_gene_freq_by_cluster_Class_switched..IGHV3.48" "156"
"V_gene_freq_by_cluster_IGHA1..IGHV3.48" "156"
"V_gene_freq_by_cluster_IGHG2..IGHV3.48" "156"
"V_gene_freq_by_uniq_VDJ_IGHG3..IGHV3.20.1" "157"
"V_gene_freq_by_uniq_VDJ_IGHG3..IGHV3.62" "157"
"V_gene_freq_by_cluster_IGHG3..IGHV3.20.1" "157"
"V_gene_freq_by_cluster_IGHG3..IGHV3.62" "157"
"Mean_cluster_size_IGHG3..IGHV3.20" "157"
"V_gene_freq_by_uniq_VDJ_IGHD.IGHM_mutated..IGHV6.1" "158"
"V_gene_freq_by_uniq_VDJ_IGHM..IGHV6.1" "158"
"V_gene_freq_by_cluster_expanded..IGHV6.1" "158"
"V_gene_freq_by_cluster_IGHD.IGHM_mutated..IGHV6.1" "158"
"V_gene_freq_by_cluster_IGHM..IGHV6.1" "158"
"V_gene_freq_by_uniq_VDJ_IGHG3..IGHV2.5.1" "159"
"V_gene_freq_by_uniq_VDJ_IGHG3..IGHV4.31.1" "159"

"V_gene_freq_by_cluster_IGHG3..IGHV2.5.1" "159"
"V_gene_freq_by_cluster_IGHG3..IGHV4.31.1" "159"
"Mean_cluster_size_IGHG3..IGHV4.31" "159"
"V_gene_freq_by_cluster_IGHE..IGHV1.18" "160"
"V_gene_freq_by_cluster_IGHE..IGHV1.2" "160"
"V_gene_freq_by_cluster_IGHE..IGHV3.49" "160"
"V_gene_freq_by_cluster_IGHE..IGHV3.9" "160"
"V_gene_freq_by_cluster_IGHE..IGHV5.51" "160"
"V_gene_freq_by_uniq_VDJ_IGHG3..IGHV3.13.1" "161"
"V_gene_freq_by_cluster_IGHG3..IGHV4.30.2.1" "161"
"V_gene_freq_by_cluster_IGHG3..IGHV4.30.4.1" "161"
"V_gene_freq_by_cluster_IGHG3..IGHV4.38.2.1" "161"
"Mean_cluster_size_IGHG3..IGHV3.13" "161"
"Relative_class_switching_absolute..IGHE.02.IGHG3.18.1" "162"
"V_gene_freq_by_uniq_VDJ_IGHE..IGHV1.58" "162"
"V_gene_freq_by_uniq_VDJ_IGHG3..IGHV1.58.1" "162"
"V_gene_freq_by_cluster_IGHG3..IGHV1.58.1" "162"
"V_gene_freq_by_cluster_IGHG3..IGHV1.69.1" "162"

Table 4 – Module and features associated with PDAC adaptive vs myeloid-enriched samples

Feature	Cluster
BCR nduplicate_clones_intra__B.cell.memory	6
BCR nduplicate_clones_intra__B.cell.MZ	12
BCR nduplicate_clones_intra__PB.PC	25
BCR nduplicate_clones_intra__non.naive	6
BCR nduplicate_clones_intra__all	6
BCR nduplicate_clones_inter__B.cell.GC	23
BCR nduplicate_clones_inter__B.cell.memory	21
BCR nduplicate_clones_inter__B.cell.MZ	12
BCR nduplicate_clones_inter__B.cell.naive	24
BCR nduplicate_clones_inter__PB.PC	10
BCR gini__B.cell.memory	6
BCR gini__B.cell.naive	26
BCR gini__all	6
BCR d5__B.cell.memory	6
BCR d5__B.cell.naive	26
BCR d5__all	29
TCR TCR_CD4_nduplicate_clones_intra__T.cell.CD4.Naive	10
TCR TCR_CD4_nduplicate_clones_intra__T.cell.CD4.Tfh	14
TCR TCR_CD4_nduplicate_clones_intra__T.cell.CD4.Th17	4
TCR TCR_CD4_nduplicate_clones_intra__T.cell.CD4.Th2	4
TCR TCR_CD4_nduplicate_clones_intra__T.cell.CD4.Treg	17
TCR TCR_CD4_nduplicate_clones_intra__non.naive	10
TCR TCR_CD4_nduplicate_clones_intra__all	10
TCR TCR_CD8_nduplicate_clones_intra__T.cell.CD8.EM	9
TCR TCR_CD8_nduplicate_clones_intra__non.naive	9
TCR TCR_CD8_nduplicate_clones_intra__all	9

TCR TCR_CD4_nduplicate_clones_inter__T.cell.CD4.CM	7
TCR TCR_CD4_nduplicate_clones_inter__T.cell.CD4.CM.Activated	15
TCR TCR_CD4_nduplicate_clones_inter__T.cell.CD4.EM	4
TCR TCR_CD4_nduplicate_clones_inter__T.cell.CD4.Exhausted	26
TCR TCR_CD4_nduplicate_clones_inter__T.cell.CD4.Naive	7
TCR TCR_CD4_nduplicate_clones_inter__T.cell.CD4.Senescent	22
TCR TCR_CD4_nduplicate_clones_inter__T.cell.CD4.Tfh	1
TCR TCR_CD4_nduplicate_clones_inter__T.cell.CD4.Tfh.Activated	26
TCR TCR_CD4_nduplicate_clones_inter__T.cell.CD4.Th1	22
TCR TCR_CD4_nduplicate_clones_inter__T.cell.CD4.Th17	4
TCR TCR_CD4_nduplicate_clones_inter__T.cell.CD4.Th17.Activated	23
TCR TCR_CD4_nduplicate_clones_inter__T.cell.CD4.Th2	4
TCR TCR_CD4_nduplicate_clones_inter__T.cell.CD4.Treg	24
TCR TCR_CD4_nduplicate_clones_inter__T.cell.CD4.Treg.Activated	24
TCR TCR_CD8_nduplicate_clones_inter__T.cell.CD8.CM.Activated	10
TCR TCR_CD8_nduplicate_clones_inter__T.cell.CD8.EM	5
TCR TCR_CD8_nduplicate_clones_inter__T.cell.CD8.EM.Activated	1
TCR TCR_CD8_nduplicate_clones_inter__T.cell.CD8.EMRA	22
TCR TCR_CD8_nduplicate_clones_inter__T.cell.CD8.Exhausted	28
TCR TCR_CD8_nduplicate_clones_inter__T.cell.CD8.Naive	12
TCR TCR_CD8_nduplicate_clones_inter__T.cell.CD8.Senescent	22
TCR TCR_CD8_nduplicate_clones_inter__T.cell.MAIT	7
BCR isotype usage|B cell memory:broad|IGHA1	24
BCR isotype usage|B cell memory:broad|IGHA2	14
BCR isotype usage|B cell memory:broad|IGHD	10
BCR isotype usage|B cell memory:broad|IGHG1	4
BCR isotype usage|B cell memory:broad|IGHG2	12
BCR isotype usage|B cell memory:broad|IGHG3	12
BCR isotype usage|B cell memory:broad|IGHG4	14
BCR isotype usage|B cell memory:broad|IGHM	9
BCR isotype usage|B cell MZ:broad|IGHA1	7
BCR isotype usage|B cell MZ:broad|IGHD	7
BCR isotype usage|B cell MZ:broad|IGHG1	19
BCR isotype usage|B cell MZ:broad|IGHG3	12
BCR isotype usage|B cell MZ:broad|IGHM	30
BCR isotype usage|B cell naive:broad|IGHD	10
BCR isotype usage|B cell naive:broad|IGHM	25
BCR isotype usage|PB/PC:broad|IGHA1	18
BCR isotype usage|PB/PC:broad|IGHA2	18
BCR isotype usage|PB/PC:broad|IGHG1	26
BCR isotype usage|PB/PC:broad|IGHG2	12
BCR isotype usage|PB/PC:broad|IGHG3	4
BCR isotype usage|PB/PC:broad|IGHM	23
BCR isotype usage|B cell plasma cell:detailed|IGHA1	18
BCR isotype usage|B cell plasma cell:detailed|IGHA2	18
BCR isotype usage|B cell plasma cell:detailed|IGHG1	26
BCR isotype usage|B cell plasma cell:detailed|IGHG2	12
BCR isotype usage|B cell plasma cell:detailed|IGHM	23
BCR isotype usage|B cell activated pre-memory:detailed|IGHA1	24

BCR isotype usage|B cell activated pre-memory:detailed|IGHD 10
BCR isotype usage|B cell activated pre-memory:detailed|IGHG1 11
BCR isotype usage|B cell activated pre-memory:detailed|IGHM 20
BCR isotype usage|B cell memory:detailed|IGHA1 24
BCR isotype usage|B cell memory:detailed|IGHA2 14
BCR isotype usage|B cell memory:detailed|IGHD 10
BCR isotype usage|B cell memory:detailed|IGHG1 4
BCR isotype usage|B cell memory:detailed|IGHG2 3
BCR isotype usage|B cell memory:detailed|IGHG3 12
BCR isotype usage|B cell memory:detailed|IGHG4 14
BCR isotype usage|B cell memory:detailed|IGHM 9
BCR isotype usage|B cell memory activated:detailed|IGHA1 24
BCR isotype usage|B cell memory activated:detailed|IGHA2 14
BCR isotype usage|B cell memory activated:detailed|IGHD 10
BCR isotype usage|B cell memory activated:detailed|IGHG1 4
BCR isotype usage|B cell memory activated:detailed|IGHG2 12
BCR isotype usage|B cell memory activated:detailed|IGHG3 7
BCR isotype usage|B cell memory activated:detailed|IGHM9
BCR isotype usage|B cell MZ:detailed|IGHA1 7
BCR isotype usage|B cell MZ:detailed|IGHD 7
BCR isotype usage|B cell MZ:detailed|IGHG1 19
BCR isotype usage|B cell MZ:detailed|IGHG3 12
BCR isotype usage|B cell MZ:detailed|IGHM30
BCR isotype usage|B cell naive:detailed|IGHD 10
BCR isotype usage|B cell naive:detailed|IGHM 25
BCR isotype usage|All|IGHA124
BCR isotype usage|All|IGHA224
BCR isotype usage|All|IGHD 10
BCR isotype usage|All|IGHG14
BCR isotype usage|All|IGHG212
BCR isotype usage|All|IGHG312
BCR isotype usage|All|IGHG414
BCR isotype usage|All|IGHM 9
BCR IGK/L usage|B cell memory:broad|IGK 18
BCR IGK/L usage|B cell memory:broad|IGL 23
BCR IGK/L usage|B cell MZ:broad|IGK 4
BCR IGK/L usage|B cell MZ:broad|IGL 9
BCR IGK/L usage|B cell naive:broad|IGK 11
BCR IGK/L usage|B cell naive:broad|IGL 20
BCR IGK/L usage|PB/PC:broad|IGK 13
BCR IGK/L usage|PB/PC:broad|IGL 4
BCR IGK/L usage|B cell plasma cell:detailed|IGK 13
BCR IGK/L usage|B cell plasma cell:detailed|IGL 4
BCR IGK/L usage|B cell activated pre-memory:detailed|IGK18
BCR IGK/L usage|B cell activated pre-memory:detailed|IGL 23
BCR IGK/L usage|B cell memory:detailed|IGK 18
BCR IGK/L usage|B cell memory:detailed|IGL 23
BCR IGK/L usage|B cell memory activated:detailed|IGK 18
BCR IGK/L usage|B cell memory activated:detailed|IGL 23

BCR IGK/L usage|B cell MZ:detailed|IGK 4
BCR IGK/L usage|B cell MZ:detailed|IGL 9
BCR IGK/L usage|B cell naive:detailed|IGK 11
BCR IGK/L usage|B cell naive:detailed|IGL 20
BCR IGK/L usage|All|IGK 18
BCR IGK/L usage|All|IGL 23
BCR mean SHM IGH|B cell memory:broad|IGHA1 14
BCR mean SHM IGH|B cell memory:broad|IGHA2 7
BCR mean SHM IGH|B cell memory:broad|IGHG1 4
BCR mean SHM IGH|B cell memory:broad|IGHM 6
BCR mean SHM IGH|B cell naive:broad|IGHM 26
BCR mean SHM IGH|B cell activated pre-memory:detailed|IGHM 6
BCR mean SHM IGH|B cell memory:detailed|IGHA1 14
BCR mean SHM IGH|B cell memory:detailed|IGHG1 4
BCR mean SHM IGH|B cell memory:detailed|IGHM 6
BCR mean SHM IGH|B cell memory activated:detailed|IGHA1 17
BCR mean SHM IGH|B cell memory activated:detailed|IGHM 6
BCR mean SHM IGH|B cell naive:detailed|IGHM 26
BCR mean SHM IGH|All|IGHA1 27
BCR mean SHM IGH|All|IGHA2 7
BCR mean SHM IGH|All|IGHD 14
BCR mean SHM IGH|All|IGHG1 4
BCR mean SHM IGH|All|IGHM 6
BCR mean SHM IGK/L|B cell memory:broad|IGHA1 14
BCR mean SHM IGK/L|B cell memory:broad|IGHA2 7
BCR mean SHM IGK/L|B cell memory:broad|IGHG1 4
BCR mean SHM IGK/L|B cell memory:broad|IGHM 6
BCR mean SHM IGK/L|B cell naive:broad|IGHM 30
BCR mean SHM IGK/L|B cell activated pre-memory:detailed|IGHM 6
BCR mean SHM IGK/L|B cell memory:detailed|IGHA1 14
BCR mean SHM IGK/L|B cell memory:detailed|IGHG1 4
BCR mean SHM IGK/L|B cell memory:detailed|IGHM 6
BCR mean SHM IGK/L|B cell memory activated:detailed|IGHA1 17
BCR mean SHM IGK/L|B cell memory activated:detailed|IGHM 6
BCR mean SHM IGK/L|B cell naive:detailed|IGHM 30
BCR mean SHM IGK/L|All|IGHA1 27
BCR mean SHM IGK/L|All|IGHA2 7
BCR mean SHM IGK/L|All|IGHD 14
BCR mean SHM IGK/L|All|IGHG1 4
BCR mean SHM IGK/L|All|IGHM 6
BCR mean SHM IGH+K/L|B cell memory:broad|IGHA1 14
BCR mean SHM IGH+K/L|B cell memory:broad|IGHA2 7
BCR mean SHM IGH+K/L|B cell memory:broad|IGHG1 4
BCR mean SHM IGH+K/L|B cell memory:broad|IGHM 6
BCR mean SHM IGH+K/L|B cell naive:broad|IGHM 30
BCR mean SHM IGH+K/L|B cell activated pre-memory:detailed|IGHM 6
BCR mean SHM IGH+K/L|B cell memory:detailed|IGHA1 14
BCR mean SHM IGH+K/L|B cell memory:detailed|IGHG1 4
BCR mean SHM IGH+K/L|B cell memory:detailed|IGHM 6

BCR mean SHM IGH+K/L|B cell memory activated:detailed|IGHA1 17
BCR mean SHM IGH+K/L|B cell memory activated:detailed|IGHM 6
BCR mean SHM IGH+K/L|B cell naive:detailed|IGHM 30
BCR mean SHM IGH+K/L|All|IGHA1 27
BCR mean SHM IGH+K/L|All|IGHA2 7
BCR mean SHM IGH+K/L|All|IGHD 14
BCR mean SHM IGH+K/L|All|IGHG1 4
BCR mean SHM IGH+K/L|All|IGHM 6
KEGG_APOPTOSIS|B cell plasma cell 13
KEGG_APOPTOSIS|B cell activated pre-memory 11
KEGG_APOPTOSIS|B cell memory 11
KEGG_APOPTOSIS|B cell memory activated 11
KEGG_APOPTOSIS|B cell MZ 20
KEGG_APOPTOSIS|B cell naive 10
KEGG_APOPTOSIS|NK cell NK active 24
KEGG_APOPTOSIS|NK cell NK CD56bright 7
KEGG_APOPTOSIS|NK cell NK like T cell 1
KEGG_APOPTOSIS|NK cell NK mature 3
KEGG_APOPTOSIS|NK cell NK transitional 21
KEGG_APOPTOSIS|T cell CD4 Activated Tfh 1
KEGG_APOPTOSIS|T cell CD4 Activated Th17 1
KEGG_APOPTOSIS|T cell CD4 Activated Treg 1
KEGG_APOPTOSIS|T cell CD4 CM 1
KEGG_APOPTOSIS|T cell CD4 EM 1
KEGG_APOPTOSIS|T cell CD4 Naive 16
KEGG_APOPTOSIS|T cell CD4 Tfh 1
KEGG_APOPTOSIS|T cell CD4 Th1 26
KEGG_APOPTOSIS|T cell CD4 Th17 1
KEGG_APOPTOSIS|T cell CD4 Th2 1
KEGG_APOPTOSIS|T cell CD4 Treg 1
KEGG_APOPTOSIS|T cell CD8 Activated EM 21
KEGG_APOPTOSIS|T cell CD8 Activated Exhausted 1
KEGG_APOPTOSIS|T cell CD8 EM 1
KEGG_APOPTOSIS|T cell CD8 EMRA 1
KEGG_APOPTOSIS|T cell CD8 Naive 30
KEGG_APOPTOSIS|T cell CD8 Senescent 1
KEGG_APOPTOSIS|T cell ILC1 30
KEGG_APOPTOSIS|T cell ILC3 21
KEGG_APOPTOSIS|T cell MAIT 13
KEGG_T_CELL_RECEPTOR_SIGNALING_PATHWAY|NK cell NK active 16
KEGG_T_CELL_RECEPTOR_SIGNALING_PATHWAY|NK cell NK CD56bright 13
KEGG_T_CELL_RECEPTOR_SIGNALING_PATHWAY|NK cell NK like T cell 1
KEGG_T_CELL_RECEPTOR_SIGNALING_PATHWAY|NK cell NK mature 21
KEGG_T_CELL_RECEPTOR_SIGNALING_PATHWAY|NK cell NK transitional 21
KEGG_T_CELL_RECEPTOR_SIGNALING_PATHWAY|T cell CD4 Activated Tfh 1
KEGG_T_CELL_RECEPTOR_SIGNALING_PATHWAY|T cell CD4 Activated Th17 1
KEGG_T_CELL_RECEPTOR_SIGNALING_PATHWAY|T cell CD4 Activated Treg 1
KEGG_T_CELL_RECEPTOR_SIGNALING_PATHWAY|T cell CD4 CM 1
KEGG_T_CELL_RECEPTOR_SIGNALING_PATHWAY|T cell CD4 EM 1

KEGG_T_CELL_RECEPTOR_SIGNALING_PATHWAY|T cell CD4 Naive 1
KEGG_T_CELL_RECEPTOR_SIGNALING_PATHWAY|T cell CD4 Tfh 1
KEGG_T_CELL_RECEPTOR_SIGNALING_PATHWAY|T cell CD4 Th1 26
KEGG_T_CELL_RECEPTOR_SIGNALING_PATHWAY|T cell CD4 Th17 1
KEGG_T_CELL_RECEPTOR_SIGNALING_PATHWAY|T cell CD4 Th2 1
KEGG_T_CELL_RECEPTOR_SIGNALING_PATHWAY|T cell CD4 Treg 1
KEGG_T_CELL_RECEPTOR_SIGNALING_PATHWAY|T cell CD8 Activated EM 21
KEGG_T_CELL_RECEPTOR_SIGNALING_PATHWAY|T cell CD8 Activated Exhausted 1
KEGG_T_CELL_RECEPTOR_SIGNALING_PATHWAY|T cell CD8 EM 1
KEGG_T_CELL_RECEPTOR_SIGNALING_PATHWAY|T cell CD8 EMRA 1
KEGG_T_CELL_RECEPTOR_SIGNALING_PATHWAY|T cell CD8 Naive 1
KEGG_T_CELL_RECEPTOR_SIGNALING_PATHWAY|T cell CD8 Senescent 1
KEGG_T_CELL_RECEPTOR_SIGNALING_PATHWAY|T cell ILC1 21
KEGG_T_CELL_RECEPTOR_SIGNALING_PATHWAY|T cell ILC3 21
KEGG_T_CELL_RECEPTOR_SIGNALING_PATHWAY|T cell MAIT 21
KEGG_B_CELL_RECEPTOR_SIGNALING_PATHWAY|B cell plasma cell 21
KEGG_B_CELL_RECEPTOR_SIGNALING_PATHWAY|B cell activated pre-memory 20
KEGG_B_CELL_RECEPTOR_SIGNALING_PATHWAY|B cell memory 20
KEGG_B_CELL_RECEPTOR_SIGNALING_PATHWAY|B cell memory activated20
KEGG_B_CELL_RECEPTOR_SIGNALING_PATHWAY|B cell MZ20
KEGG_B_CELL_RECEPTOR_SIGNALING_PATHWAY|B cell naive 20
HALLMARK_HYPOXIA|B cell plasma cell 5
HALLMARK_HYPOXIA|B cell activated pre-memory 5
HALLMARK_HYPOXIA|B cell memory5
HALLMARK_HYPOXIA|B cell memory activated 5
HALLMARK_HYPOXIA|B cell MZ 5
HALLMARK_HYPOXIA|B cell naive 5
HALLMARK_HYPOXIA|NK cell NK active 16
HALLMARK_HYPOXIA|NK cell NK CD56bright 16
HALLMARK_HYPOXIA|NK cell NK like T cell 16
HALLMARK_HYPOXIA|NK cell NK mature 16
HALLMARK_HYPOXIA|NK cell NK transitional 16
HALLMARK_HYPOXIA|T cell CD4 Activated Tfh 5
HALLMARK_HYPOXIA|T cell CD4 Activated Th17 5
HALLMARK_HYPOXIA|T cell CD4 Activated Treg 5
HALLMARK_HYPOXIA|T cell CD4 CM 5
HALLMARK_HYPOXIA|T cell CD4 EM 5
HALLMARK_HYPOXIA|T cell CD4 Naive 5
HALLMARK_HYPOXIA|T cell CD4 Tfh 5
HALLMARK_HYPOXIA|T cell CD4 Th1 16
HALLMARK_HYPOXIA|T cell CD4 Th17 5
HALLMARK_HYPOXIA|T cell CD4 Th2 5
HALLMARK_HYPOXIA|T cell CD4 Treg 5
HALLMARK_HYPOXIA|T cell CD8 Activated EM 16
HALLMARK_HYPOXIA|T cell CD8 Activated Exhausted 5
HALLMARK_HYPOXIA|T cell CD8 EM 5
HALLMARK_HYPOXIA|T cell CD8 EMRA 16
HALLMARK_HYPOXIA|T cell CD8 Naive 16
HALLMARK_HYPOXIA|T cell CD8 Senescent 16

HALLMARK_HYPOXIA|T cell ILC1 5
HALLMARK_HYPOXIA|T cell ILC3 5
HALLMARK_HYPOXIA|T cell MAIT 16
GO_RESPONSE_TO_TYPE_I_INTERFERON|B cell plasma cell 8
GO_RESPONSE_TO_TYPE_I_INTERFERON|B cell activated pre-memory 8
GO_RESPONSE_TO_TYPE_I_INTERFERON|B cell memory 8
GO_RESPONSE_TO_TYPE_I_INTERFERON|B cell memory activated 8
GO_RESPONSE_TO_TYPE_I_INTERFERON|B cell MZ 11
GO_RESPONSE_TO_TYPE_I_INTERFERON|B cell naive 8
GO_RESPONSE_TO_TYPE_I_INTERFERON|NK cell NK active 2
GO_RESPONSE_TO_TYPE_I_INTERFERON|NK cell NK CD56bright 2
GO_RESPONSE_TO_TYPE_I_INTERFERON|NK cell NK like T cell 2
GO_RESPONSE_TO_TYPE_I_INTERFERON|NK cell NK mature 15
GO_RESPONSE_TO_TYPE_I_INTERFERON|NK cell NK transitional 2
GO_RESPONSE_TO_TYPE_I_INTERFERON|T cell CD4 Activated Tfh 8
GO_RESPONSE_TO_TYPE_I_INTERFERON|T cell CD4 Activated Th17 8
GO_RESPONSE_TO_TYPE_I_INTERFERON|T cell CD4 Activated Treg 8
GO_RESPONSE_TO_TYPE_I_INTERFERON|T cell CD4 CM 19
GO_RESPONSE_TO_TYPE_I_INTERFERON|T cell CD4 EM 8
GO_RESPONSE_TO_TYPE_I_INTERFERON|T cell CD4 Naive 19
GO_RESPONSE_TO_TYPE_I_INTERFERON|T cell CD4 Tfh 15
GO_RESPONSE_TO_TYPE_I_INTERFERON|T cell CD4 Th1 19
GO_RESPONSE_TO_TYPE_I_INTERFERON|T cell CD4 Th17 15
GO_RESPONSE_TO_TYPE_I_INTERFERON|T cell CD4 Th2 15
GO_RESPONSE_TO_TYPE_I_INTERFERON|T cell CD4 Treg 8
GO_RESPONSE_TO_TYPE_I_INTERFERON|T cell CD8 Activated EM 15
GO_RESPONSE_TO_TYPE_I_INTERFERON|T cell CD8 Activated Exhausted 8
GO_RESPONSE_TO_TYPE_I_INTERFERON|T cell CD8 EM 15
GO_RESPONSE_TO_TYPE_I_INTERFERON|T cell CD8 EMRA 19
GO_RESPONSE_TO_TYPE_I_INTERFERON|T cell CD8 Naive 19
GO_RESPONSE_TO_TYPE_I_INTERFERON|T cell CD8 Senescent 19
GO_RESPONSE_TO_TYPE_I_INTERFERON|T cell ILC1 2
GO_RESPONSE_TO_TYPE_I_INTERFERON|T cell ILC3 2
GO_RESPONSE_TO_TYPE_I_INTERFERON|T cell MAIT 7
GO_T_CELL_PROLIFERATION|NK cell NK active 15
GO_T_CELL_PROLIFERATION|NK cell NK CD56bright25
GO_T_CELL_PROLIFERATION|NK cell NK like T cell 3
GO_T_CELL_PROLIFERATION|NK cell NK mature 21
GO_T_CELL_PROLIFERATION|NK cell NK transitional 29
GO_T_CELL_PROLIFERATION|T cell CD4 Activated Tfh 1
GO_T_CELL_PROLIFERATION|T cell CD4 Activated Th17 29
GO_T_CELL_PROLIFERATION|T cell CD4 Activated Treg 5
GO_T_CELL_PROLIFERATION|T cell CD4 CM 3
GO_T_CELL_PROLIFERATION|T cell CD4 EM 10
GO_T_CELL_PROLIFERATION|T cell CD4 Naive 29
GO_T_CELL_PROLIFERATION|T cell CD4 Tfh 3
GO_T_CELL_PROLIFERATION|T cell CD4 Th1 11
GO_T_CELL_PROLIFERATION|T cell CD4 Th17 29
GO_T_CELL_PROLIFERATION|T cell CD4 Th2 29

GO_T_CELL_PROLIFERATION|T cell CD4 Treg 3
GO_T_CELL_PROLIFERATION|T cell CD8 Activated EM 29
GO_T_CELL_PROLIFERATION|T cell CD8 Activated Exhausted 13
GO_T_CELL_PROLIFERATION|T cell CD8 EM 29
GO_T_CELL_PROLIFERATION|T cell CD8 EMRA 29
GO_T_CELL_PROLIFERATION|T cell CD8 Naive 3
GO_T_CELL_PROLIFERATION|T cell CD8 Senescent 3
GO_T_CELL_PROLIFERATION|T cell ILC1 23
GO_T_CELL_PROLIFERATION|T cell ILC3 16
GO_T_CELL_PROLIFERATION|T cell MAIT 7
GO_B_CELL_PROLIFERATION|B cell plasma cell 23
GO_B_CELL_PROLIFERATION|B cell activated pre-memory 20
GO_B_CELL_PROLIFERATION|B cell memory 20
GO_B_CELL_PROLIFERATION|B cell memory activated 20
GO_B_CELL_PROLIFERATION|B cell MZ 20
GO_B_CELL_PROLIFERATION|B cell naive 20
GO_RESPONSE_TO_INTERFERON_BETA|B cell plasma cell 24
GO_RESPONSE_TO_INTERFERON_BETA|B cell activated pre-memory 11
GO_RESPONSE_TO_INTERFERON_BETA|B cell memory 11
GO_RESPONSE_TO_INTERFERON_BETA|B cell memory activated 11
GO_RESPONSE_TO_INTERFERON_BETA|B cell MZ 11
GO_RESPONSE_TO_INTERFERON_BETA|B cell naive 11
GO_RESPONSE_TO_INTERFERON_BETA|NK cell NK active 2
GO_RESPONSE_TO_INTERFERON_BETA|NK cell NK CD56bright 2
GO_RESPONSE_TO_INTERFERON_BETA|NK cell NK like T cell 2
GO_RESPONSE_TO_INTERFERON_BETA|NK cell NK mature 10
GO_RESPONSE_TO_INTERFERON_BETA|NK cell NK transitional 2
GO_RESPONSE_TO_INTERFERON_BETA|T cell CD4 Activated Tfh 17
GO_RESPONSE_TO_INTERFERON_BETA|T cell CD4 Activated Th17 17
GO_RESPONSE_TO_INTERFERON_BETA|T cell CD4 Activated Treg 17
GO_RESPONSE_TO_INTERFERON_BETA|T cell CD4 CM 17
GO_RESPONSE_TO_INTERFERON_BETA|T cell CD4 EM 22
GO_RESPONSE_TO_INTERFERON_BETA|T cell CD4 Naive 17
GO_RESPONSE_TO_INTERFERON_BETA|T cell CD4 Tfh 17
GO_RESPONSE_TO_INTERFERON_BETA|T cell CD4 Th1 2
GO_RESPONSE_TO_INTERFERON_BETA|T cell CD4 Th17 22
GO_RESPONSE_TO_INTERFERON_BETA|T cell CD4 Th2 17
GO_RESPONSE_TO_INTERFERON_BETA|T cell CD4 Treg 17
GO_RESPONSE_TO_INTERFERON_BETA|T cell CD8 Activated EM 22
GO_RESPONSE_TO_INTERFERON_BETA|T cell CD8 Activated Exhausted 28
GO_RESPONSE_TO_INTERFERON_BETA|T cell CD8 EM 22
GO_RESPONSE_TO_INTERFERON_BETA|T cell CD8 EMRA 22
GO_RESPONSE_TO_INTERFERON_BETA|T cell CD8 Naive 22
GO_RESPONSE_TO_INTERFERON_BETA|T cell CD8 Senescent 2
GO_RESPONSE_TO_INTERFERON_BETA|T cell ILC1 10
GO_RESPONSE_TO_INTERFERON_BETA|T cell ILC3 2
GO_RESPONSE_TO_INTERFERON_BETA|T cell MAIT 7
GO_RESPONSE_TO_INTERFERON_ALPHA|B cell plasma cell 24
GO_RESPONSE_TO_INTERFERON_ALPHA|B cell activated pre-memory 11

GO_RESPONSE_TO_INTERFERON_ALPHA|B cell memory	11
GO_RESPONSE_TO_INTERFERON_ALPHA|B cell memory activated	25
GO_RESPONSE_TO_INTERFERON_ALPHA|B cell MZ	11
GO_RESPONSE_TO_INTERFERON_ALPHA|B cell naive	8
GO_RESPONSE_TO_INTERFERON_ALPHA|NK cell NK active	2
GO_RESPONSE_TO_INTERFERON_ALPHA|NK cell NK CD56bright	2
GO_RESPONSE_TO_INTERFERON_ALPHA|NK cell NK like T cell	2
GO_RESPONSE_TO_INTERFERON_ALPHA|NK cell NK mature	10
GO_RESPONSE_TO_INTERFERON_ALPHA|NK cell NK transitional	2
GO_RESPONSE_TO_INTERFERON_ALPHA|T cell CD4 Activated Tfh	8
GO_RESPONSE_TO_INTERFERON_ALPHA|T cell CD4 Activated Th17	8
GO_RESPONSE_TO_INTERFERON_ALPHA|T cell CD4 Activated Treg	25
GO_RESPONSE_TO_INTERFERON_ALPHA|T cell CD4 CM	2
GO_RESPONSE_TO_INTERFERON_ALPHA|T cell CD4 EM	22
GO_RESPONSE_TO_INTERFERON_ALPHA|T cell CD4 Naive	17
GO_RESPONSE_TO_INTERFERON_ALPHA|T cell CD4 Tfh	2
GO_RESPONSE_TO_INTERFERON_ALPHA|T cell CD4 Th1	2
GO_RESPONSE_TO_INTERFERON_ALPHA|T cell CD4 Th17	2
GO_RESPONSE_TO_INTERFERON_ALPHA|T cell CD4 Th2	2
GO_RESPONSE_TO_INTERFERON_ALPHA|T cell CD4 Treg	8
GO_RESPONSE_TO_INTERFERON_ALPHA|T cell CD8 Activated EM	2
GO_RESPONSE_TO_INTERFERON_ALPHA|T cell CD8 Activated Exhausted	11
GO_RESPONSE_TO_INTERFERON_ALPHA|T cell CD8 EM	2
GO_RESPONSE_TO_INTERFERON_ALPHA|T cell CD8 EMRA	22
GO_RESPONSE_TO_INTERFERON_ALPHA|T cell CD8 Naive	22
GO_RESPONSE_TO_INTERFERON_ALPHA|T cell CD8 Senescent	2
GO_RESPONSE_TO_INTERFERON_ALPHA|T cell ILC1	10
GO_RESPONSE_TO_INTERFERON_ALPHA|T cell ILC3	2
GO_RESPONSE_TO_INTERFERON_ALPHA|T cell MAIT	7
GO_INTERFERON_GAMMA_PRODUCTION|B cell plasma cell	18
GO_INTERFERON_GAMMA_PRODUCTION|B cell activated pre-memory	18
GO_INTERFERON_GAMMA_PRODUCTION|B cell memory	18
GO_INTERFERON_GAMMA_PRODUCTION|B cell memory activated	18
GO_INTERFERON_GAMMA_PRODUCTION|B cell MZ	11
GO_INTERFERON_GAMMA_PRODUCTION|B cell naive	18
GO_INTERFERON_GAMMA_PRODUCTION|NK cell NK active	8
GO_INTERFERON_GAMMA_PRODUCTION|NK cell NK CD56bright	8
GO_INTERFERON_GAMMA_PRODUCTION|NK cell NK like T cell	8
GO_INTERFERON_GAMMA_PRODUCTION|NK cell NK mature	21
GO_INTERFERON_GAMMA_PRODUCTION|NK cell NK transitional	3
GO_INTERFERON_GAMMA_PRODUCTION|T cell CD4 Activated Tfh	3
GO_INTERFERON_GAMMA_PRODUCTION|T cell CD4 Activated Th17	3
GO_INTERFERON_GAMMA_PRODUCTION|T cell CD4 Activated Treg	3
GO_INTERFERON_GAMMA_PRODUCTION|T cell CD4 CM	3
GO_INTERFERON_GAMMA_PRODUCTION|T cell CD4 EM	3
GO_INTERFERON_GAMMA_PRODUCTION|T cell CD4 Naive	3
GO_INTERFERON_GAMMA_PRODUCTION|T cell CD4 Tfh	3
GO_INTERFERON_GAMMA_PRODUCTION|T cell CD4 Th1	8
GO_INTERFERON_GAMMA_PRODUCTION|T cell CD4 Th17	3

GO_INTERFERON_GAMMA_PRODUCTION|T cell CD4 Th2	3
GO_INTERFERON_GAMMA_PRODUCTION|T cell CD4 Treg	3
GO_INTERFERON_GAMMA_PRODUCTION|T cell CD8 Activated EM	3
GO_INTERFERON_GAMMA_PRODUCTION|T cell CD8 Activated Exhausted	3
GO_INTERFERON_GAMMA_PRODUCTION|T cell CD8 EM	3
GO_INTERFERON_GAMMA_PRODUCTION|T cell CD8 EMRA	3
GO_INTERFERON_GAMMA_PRODUCTION|T cell CD8 Naive	3
GO_INTERFERON_GAMMA_PRODUCTION|T cell CD8 Senescent	3
GO_INTERFERON_GAMMA_PRODUCTION|T cell ILC1	29
GO_INTERFERON_GAMMA_PRODUCTION|T cell ILC3	15
GO_INTERFERON_GAMMA_PRODUCTION|T cell MAIT	21
GO_RESPONSE_TO_INTERFERON_GAMMA|B cell plasma cell	23
GO_RESPONSE_TO_INTERFERON_GAMMA|B cell activated pre-memory	25
GO_RESPONSE_TO_INTERFERON_GAMMA|B cell memory	25
GO_RESPONSE_TO_INTERFERON_GAMMA|B cell memory activated	25
GO_RESPONSE_TO_INTERFERON_GAMMA|B cell MZ	12
GO_RESPONSE_TO_INTERFERON_GAMMA|B cell naive	25
GO_RESPONSE_TO_INTERFERON_GAMMA|NK cell NK active	28
GO_RESPONSE_TO_INTERFERON_GAMMA|NK cell NK CD56bright	19
GO_RESPONSE_TO_INTERFERON_GAMMA|NK cell NK like T cell	28
GO_RESPONSE_TO_INTERFERON_GAMMA|NK cell NK mature	30
GO_RESPONSE_TO_INTERFERON_GAMMA|NK cell NK transitional	19
GO_RESPONSE_TO_INTERFERON_GAMMA|T cell CD4 Activated Tfh	27
GO_RESPONSE_TO_INTERFERON_GAMMA|T cell CD4 Activated Th17	26
GO_RESPONSE_TO_INTERFERON_GAMMA|T cell CD4 Activated Treg	25
GO_RESPONSE_TO_INTERFERON_GAMMA|T cell CD4 CM	27
GO_RESPONSE_TO_INTERFERON_GAMMA|T cell CD4 EM	17
GO_RESPONSE_TO_INTERFERON_GAMMA|T cell CD4 Naive	27
GO_RESPONSE_TO_INTERFERON_GAMMA|T cell CD4 Tfh	27
GO_RESPONSE_TO_INTERFERON_GAMMA|T cell CD4 Th1	28
GO_RESPONSE_TO_INTERFERON_GAMMA|T cell CD4 Th17	27
GO_RESPONSE_TO_INTERFERON_GAMMA|T cell CD4 Th2	27
GO_RESPONSE_TO_INTERFERON_GAMMA|T cell CD4 Treg	25
GO_RESPONSE_TO_INTERFERON_GAMMA|T cell CD8 Activated EM	28
GO_RESPONSE_TO_INTERFERON_GAMMA|T cell CD8 Activated Exhausted	28
GO_RESPONSE_TO_INTERFERON_GAMMA|T cell CD8 EM	28
GO_RESPONSE_TO_INTERFERON_GAMMA|T cell CD8 EMRA	28
GO_RESPONSE_TO_INTERFERON_GAMMA|T cell CD8 Naive	27
GO_RESPONSE_TO_INTERFERON_GAMMA|T cell CD8 Senescent	28
GO_RESPONSE_TO_INTERFERON_GAMMA|T cell ILC1	30
GO_RESPONSE_TO_INTERFERON_GAMMA|T cell ILC3	15
GO_RESPONSE_TO_INTERFERON_GAMMA|T cell MAIT	16
GO_B_CELL_RECEPTOR_SIGNALING_PATHWAY|B cell plasma cell	9
GO_B_CELL_RECEPTOR_SIGNALING_PATHWAY|B cell activated pre-memory	15
GO_B_CELL_RECEPTOR_SIGNALING_PATHWAY|B cell memory	15
GO_B_CELL_RECEPTOR_SIGNALING_PATHWAY|B cell memory activated	15
GO_B_CELL_RECEPTOR_SIGNALING_PATHWAY|B cell MZ	15
GO_B_CELL_RECEPTOR_SIGNALING_PATHWAY|B cell naive	15
GO_CELLULAR_RESPONSE_TO_INTERFERON_BETA|B cell plasma cell	15

GO_CELLULAR_RESPONSE_TO_INTERFERON_BETA|B cell activated pre-memory	12
GO_CELLULAR_RESPONSE_TO_INTERFERON_BETA|B cell memory	12
GO_CELLULAR_RESPONSE_TO_INTERFERON_BETA|B cell memory activated	19
GO_CELLULAR_RESPONSE_TO_INTERFERON_BETA|B cell MZ	19
GO_CELLULAR_RESPONSE_TO_INTERFERON_BETA|B cell naive	9
GO_CELLULAR_RESPONSE_TO_INTERFERON_BETA|NK cell NK active	9
GO_CELLULAR_RESPONSE_TO_INTERFERON_BETA|NK cell NK CD56bright	9
GO_CELLULAR_RESPONSE_TO_INTERFERON_BETA|NK cell NK like T cell	2
GO_CELLULAR_RESPONSE_TO_INTERFERON_BETA|NK cell NK mature	9
GO_CELLULAR_RESPONSE_TO_INTERFERON_BETA|NK cell NK transitional	9
GO_CELLULAR_RESPONSE_TO_INTERFERON_BETA|T cell CD4 Activated Tfh	13
GO_CELLULAR_RESPONSE_TO_INTERFERON_BETA|T cell CD4 Activated Th17	13
GO_CELLULAR_RESPONSE_TO_INTERFERON_BETA|T cell CD4 Activated Treg	19
GO_CELLULAR_RESPONSE_TO_INTERFERON_BETA|T cell CD4 CM	13
GO_CELLULAR_RESPONSE_TO_INTERFERON_BETA|T cell CD4 EM	12
GO_CELLULAR_RESPONSE_TO_INTERFERON_BETA|T cell CD4 Naive	13
GO_CELLULAR_RESPONSE_TO_INTERFERON_BETA|T cell CD4 Tfh	13
GO_CELLULAR_RESPONSE_TO_INTERFERON_BETA|T cell CD4 Th1	13
GO_CELLULAR_RESPONSE_TO_INTERFERON_BETA|T cell CD4 Th17	13
GO_CELLULAR_RESPONSE_TO_INTERFERON_BETA|T cell CD4 Th2	13
GO_CELLULAR_RESPONSE_TO_INTERFERON_BETA|T cell CD4 Treg	13
GO_CELLULAR_RESPONSE_TO_INTERFERON_BETA|T cell CD8 Activated EM	19
GO_CELLULAR_RESPONSE_TO_INTERFERON_BETA|T cell CD8 Activated Exhausted	13
GO_CELLULAR_RESPONSE_TO_INTERFERON_BETA|T cell CD8 EM	9
GO_CELLULAR_RESPONSE_TO_INTERFERON_BETA|T cell CD8 EMRA	13
GO_CELLULAR_RESPONSE_TO_INTERFERON_BETA|T cell CD8 Naive	9
GO_CELLULAR_RESPONSE_TO_INTERFERON_BETA|T cell CD8 Senescent	9
GO_CELLULAR_RESPONSE_TO_INTERFERON_BETA|T cell ILC1	9
GO_CELLULAR_RESPONSE_TO_INTERFERON_BETA|T cell ILC3	2
GO_CELLULAR_RESPONSE_TO_INTERFERON_BETA|T cell MAIT	17
