# Supplementary figures and images for: VDJ-REMIX: REpertoire Module Identification and eXploration
Source: Bioinformatics. 2026 Jul 7;42(Suppl 1):btag326. doi: 10.1093/bioinformatics/btag326 (PMC13340219; doi:10.1093/bioinformatics/btag326)

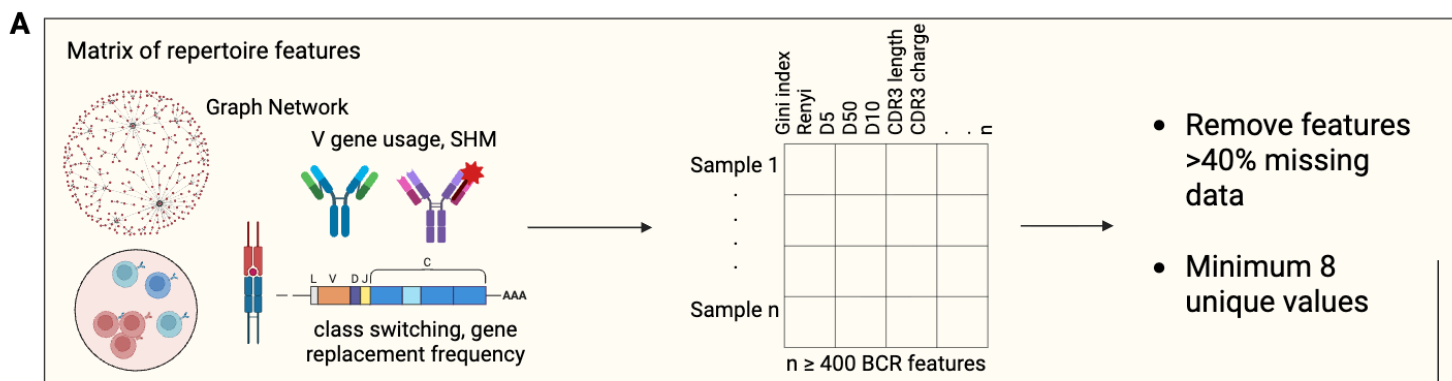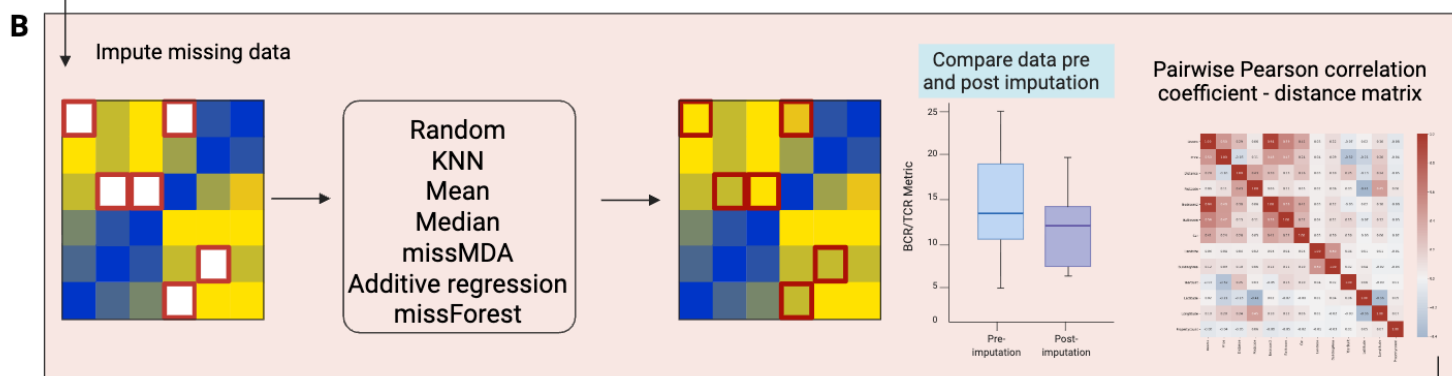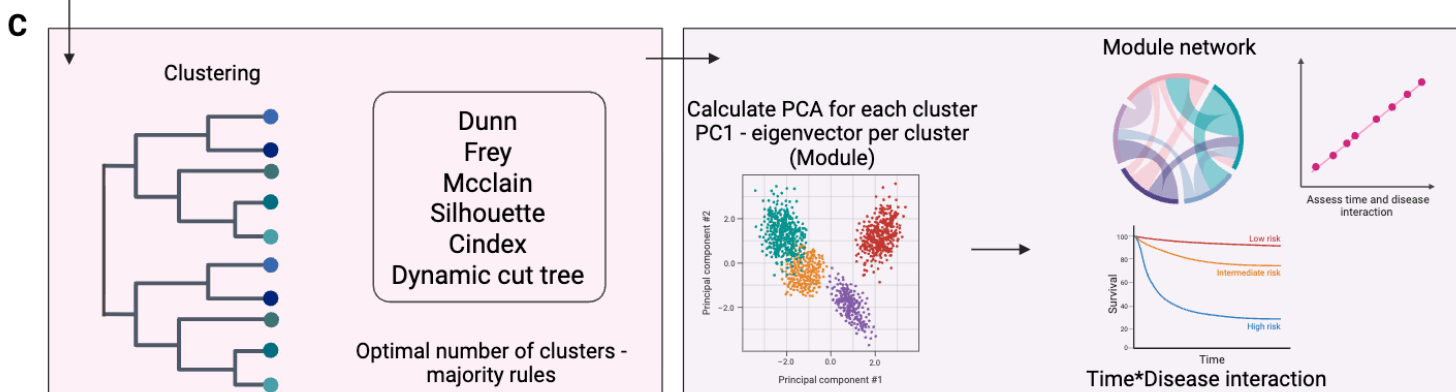

Supplement: btag326_Supplementary_Data [file btag326_supplementary_data.zip › Amin.276.Suppl.fig.1.pdf]
